# NKG2D knockdown improves hypoxic-ischemic brain damage by inhibiting neuroinflammation in neonatal mice

**DOI:** 10.1038/s41598-024-52780-3

**Published:** 2024-01-28

**Authors:** Lin Liu, Yuxin Yang, Ting Wu, Junrong Du, Fangyi Long

**Affiliations:** 1 Department of Pharmacology, Key Laboratory of Drug-Targeting and Drug Delivery System of the Education Ministry and Sichuan Province, Sichuan Engineering Laboratory for Plant-Sourced Drug and Sichuan Research Center for Drug Precision Industrial Technology, West China School of Pharmacy, Sichuan University, Chengdu, Sichuan China; 2 Department of Epidemiology and Health Statistics, West China School of Public Health and West China Fourth Hospital, Sichuan University, Chengdu, Sichuan China; 3 Laboratory Medicine Center, Sichuan Provincial Maternity and Child Health Care Hospital, Affiliated Women’s and Children’s Hospital of Chengdu Medical College, Chengdu Medical College, Chengdu, Sichuan China

**Keywords:** Diseases of the nervous system, Translational research, Brain injuries

## Abstract

Hypoxic-ischemic brain damage (HIBD) is a leading cause of neonatal death and neurological dysfunction. Neuroinflammation is identified as one of the crucial pathological mechanisms after HIBD, and natural killer group 2 member D (NKG2D) is reported to be implicated in the pathogenesis of immunoinflammatory diseases. However, the role of NKG2D in neonatal HIBD is seldomly investigated. In this study, a neonatal mice model of HIBD was induced, and the role of the NKG2D in neuroinflammation and brain injury was explored by intracerebroventricular injection of lentivirus to knockdown NKG2D in neonatal mice with HIBD. The results showed that a significant increase in NKG2D protein level in the brain of neonatal mice with HIBD. The NKG2D knockdown in the brain significantly alleviated cerebral infarction, neurobehavioral deficits, and neuronal loss in neuronal HIBD. Moreover, the neuroprotective effect of NKG2D knockdown was associated with inhibition of the activation of microglia and astrocytes, expression of NKG2D ligands (NKG2DLs) and DAP10, and the nuclear translocation of NF-κB p65. Our findings reveal NKG2D knockdown may exert anti-inflammatory and neuroprotective effects in the neonatal mice with HIBD through downregulation of NKG2D/NKG2DLs/DAP10/NF-κB pathway. These results suggest that NKG2D may be a potential target for the treatment of neonatal HIBD.

## Introduction

Neonatal hypoxic-ischemic brain damage (HIBD) refers to brain injury that is caused by lower cerebral blood flow and hypoxia in fetuses and newborns through various factors^1^. HIBD is one of the most common causes of neonatal mortality, accounting for 23% of neonatal deaths worldwide. Up to 25% of survivors develop long-term neurological deficits, such as behavioral, social, attentional, cognitive, and functional motor disorders, which increase economic and social burdens^2^. Mild hypothermia therapy is currently the most common method for treating neonatal HIBD, which needs to be performed within 6 h of birth^3^. Unfortunately, this treatment time window is often missed because of the absence of apparent symptoms in early stages of neonatal HIBD. Moreover, hypothermia has certain serious limitations for HIBD treatment, such as resulting in the physiological dysfunction of the body and even death of neonates^4^. Therefore, accurate identification of the crucial targets involved in HIBD would benefit for the development of effective therapeutic approaches for perinatal HIBD.

Numerous studies have indicated that HIBD is implicated in the complicated pathogenesis, including oxidative stress, excitotoxicity, autophagy, apoptosis, and inflammation^5–8^. HIBD mainly consists of three stages. During the first few hours of hypoxia-ischemia, cerebral blood flow, oxygen, and energy substrate levels decline rapidly, immediately triggering excitotoxicity, free radical production, and cerebral edema^9^. Secondary injury occurs in the following hours to days, and hypoxia-ischemia leads to neuroinflammation, mitochondrial dysfunction, and the loss of brain autoregulation^10^. Subsequent sustained inflammatory responses exacerbate brain damage^11^. Hypoxia-ischemia insults trigger activation of brain glial cells and blood-derived leukocytes, leading to neuroinflammatory reaction in perinatal brain^8^. Notably, the residential microglial cells rather than blood-derived macrophages are reported to contribute to the earlier and more pronounced neuroinflammatory reaction in immature brain after hypoxia-ischemia^12^. Upon hypoxic-ischemia, microglia are overactivated and produce various proinflammatory mediators, which subsequently activate astrocytes and enhance neuroinflammation, resulting in neurotoxicity and brain damage^8,12,13^. Mechanically, the transcription factor nuclear factor kappa-B (NF-κB) is identified as a key regulator for neuroinflammation in HIBD^14^. The continuous activation of NF-κB can aggravate HIBD in neonatal mice by promoting production of proinflammatory factors and neuroinflammation, whereas the inhibition of NF-κB activity significantly attenuates HIBD^14,15^. Additionally, some anti-inflammatory drugs, such as minocycline, vancomycin and erythromycin, have been reported to effectively prevent HIBD in preclinical and clinic although these antibiotics are limited to applying for the treatment of HIBD due to their adverse reactions^8^. Therefore, elucidating the immunoregulatory mechanisms of NF-κB activation in HIBD may provide the novel intervening strategy for the experimental translational studies.

Natural killer group 2 member D (NKG2D) is a C-type lectin-like transmembrane receptor that is expressed on the surface of natural killer (NK) cells, natural killer T (NKT) cell, T cells, activated CD8+T cells, subsets of CD4+T cells, and macrophages^16^. NKG2D may recognize a variety of ligands, such as human MHC class-I-related chains A or B (MICA/B) and UL16-binding proteins 1–6 (ULBP1-6), and mouse retinoic acid early transcript 1 (Rae-1) and histocompatibility antigen 60 (H60)^16^. Because of its short intracellular tail, NKG2D itself does not have a signaling ability and must combine with the adaptor molecule DNAX-activating protein 10 (DAP10) to form a hexameric compound for signal propagation^17^. On the surface of normal cells there are hardly expressed NKG2D ligands (NKG2DLs), which can be significantly upregulated under immunopathological conditions such as malignant or infected cells^18^. The NKG2D-NKG2DLs axis is involved in the development of autoimmune and inflammatory diseases (rheumatoid arthritis, colitis, celiac disease, and autoimmune diabetes) through induction of activation of the NF-κB signaling pathway^18–20^.To data, there are few reports to describe the expression of NKG2D in the central nervous system (CNS) and the potential role of the NKG2D-NKG2DLs axis in neonatal HIBD.

Therefore, the present study explored the role of NKG2D in neuroinflammation and HIBD in a neonatal mice model of cerebral hypoxia-ischemia induced by the classic Rice-Vannucci method^21^. We observed a significant increase in NKG2D expression in the brain of HIBD neonatal mice. Moreover, the NKG2D knockdown in brain by using lentivirus attenuated the infarcted volume, glial activation and neuroinflammatory response in HIBD, accompanied by inhibition of the NKG2D/NKG2DLs/DAP10/NF-κB signaling pathway. These results suggest that NKG2D may be a potential target for the treatment of neonatal HIBD.

## Materials and methods

### Animals and HIBD model

Pregnant C57BL/6 mice were purchased from Chengdu Dossy Experimental Animals Co., Ltd, and housed in a temperature (24 ± 1 °C) and relative humidity (50–60%) controlled animal facility with 12 h light/dark cycle, and freely fed and watered. All the animal procedures were approved by Chinese Animal Welfare Legislation and the Guidelines of Laboratory Animal Care and Use of Sichuan University.

Under isoflurane anesthesia (3% for induction and 2% for maintenance), neonatal mice within 24 h (no restriction in sex) were given a unilateral intracerebroventricular (i.c.v.) injection of lentiviruses that knockdown NKG2D (3 µL, 1 × 10^9^ TU/mL), control lentiviruses (3 µL, 4 × 10^8^ TU/mL), or saline (3 µL), respectively. The injection rate was 0.5 μL/min, and the stereotaxic coordinates to the bregma were 0.8 mm anterior, + 1.5 mm lateral, and 2 mm ventral^22,23^. Seven days later, the HIBD model was performed using the classic Rice-Vannucci method^21^. Under isoflurane anesthesia, an incision (about 0.5 cm) was made to the midline of anesthetized mouse neck under a dissecting microscope, after which the left common carotid artery was isolated, followed by double ligation at distal and proximal with 8–0 suture. One hour after surgery, the mice were transferred to an anoxic container with 8% O_2_ and 92% N_2_ for 2 h, and the temperature was maintained at 37 °C. The sham group mice underwent the same anesthesia and exposure as HIBD group without ligation and hypoxia treatment.

In this study, we established a neonatal mouse model of HIBD, and performed TTC staining (n = 5/group) and western blot analysis (n = 6/group) to determine the level of NKG2D in the brain tissue of HIBD mice. Then, we downregulated NKG2D expression in brain in mice by using a stereotaxic technique and lentiviral system, and the knockdown efficiency was subsequently confirmed with western blot (n = 5/group). Finally, the neonatal mice were subjected to HIBD after lentivirus administration, and TTC staining (n = 6/group), immunofluorescence experiment (n = 6/group), and western blot analysis (n = 6/group) were performed to explore the effects and molecular mechanisms of NKG2D knockdown on HIBD and neuroinflammation in neonatal mice.

### TTC staining

After 24 h of HIBD modeling, the mice were anesthetized. The brain tissue was taken out and frozen at  − 20 °C for 15 min. Coronal sections of the brain with a thickness of 1–2 mm were cut and placed in 1% TTC staining solution and stained for 30 min at 37 °C, avoiding lights. Then, the sections were fixed in 4% formaldehyde solution at 4 °C. After 24 h of fixation, the brain sections were photographed.

### Zea-Longa score

The Zea-Longa scores were used to evaluate the neurological function of mice at 24 h after HIBD. Two observers assessed the neurobehavior of mice by a blind method according to the Zea-Longa score^24^. A score of 0: no neurological deficit; a score of 1: the mouse failed to extend the right forepaw fully; a score of 2: the mouse circled while crawling; a score of 3: the mouse tumbled while walking; a score of 4: the mouse was unconscious and unable to walk spontaneously.

### Quantitative real-time polymerase chain reaction (qPCR)

Total RNA in brain tissues was extracted using Trizol (Thermo Fisher Scientific), and then the isolated RNA was reverse-transcribed into cDNA using RevertAid Fist Strand cDNA Synthesis Kit (Thermo Fisher Scientific). After that, Ssofast EvaGreen SuperMix (Bio-Rad) was used to perform the qPCR reaction on LightCycler96 real-time fluorescence quantitative PCR instrument (Roche, Basel, Switzerland). The qPCR reaction conditions were 95°C for 30 s, followed by 45 cycles of 95°C for 5 s, 60°C for 10 s and 72°C for 10 s. Primer sequences used to amplify NKG2D, H60, Rea-1, and GAPDH were as follows: F: 5’-ACCGAAAGTACTGTGGCCC-3’ and R: 5’-AGCCTGGCTCTCATACCAGT-3’ for NKG2D; F: 5’-GATGAACAGCATAGCATCTACT-3’ and R: 5’-CCTCATATCTTTCTCTAGGTTCT-3’ for H60; F: 5’-ATGGCCAAGGCAGCAGTGACCAAG-3’ and R: 5’- CACATCG-CAAATGCAAATGCAAATAAT-3’ for Rae-1 ; F: 5’-AGCGAGACCCCACTAACA TC-3’ and R: 5’-GGTTCACACCCATCACAAAC-3’ for GAPDH. The levels of H60, Rae-1 and NKG2D were normalized to GAPDH, and the data were analyzed by 2^−ΔΔCt^ method.

### Immunofluorescence staining

Brain coronal sections were fixed with 4% paraformaldehyde for 30 min and then permeabilized with 0.3% Triton X-100 (Amresco, Washington, USA) for 30 min at room temperature. Later on, they were blocked with 5% bovine serum albumin (BSA, Amresco) solution for 1 h at 37 °C. They were then incubated with anti-NeuN (1:250, HUABIO, Hangzhou, Zhejiang, China), anti-Iba-1 (1:500, Wako, Tokyo, Japan) or anti-GFAP (1:200, HUABIO) antibodies overnight at 4 °C. After washing in PBS, they were incubated with secondary antibodies of Alexa Fluor 555 labeled donkey anti-mouse (1:1000, Thermo Fisher) or Alexa Fluor 488 labelled goat anti-rabbit IgG (1:500, Thermo Fisher) for 1 h at 37 °C, respectively. Nuclei were stained with DAPI (BOSTER, Wuhan, Hebei, China) for 1 min. Pictures were subsequently taken on a fluorescence microscope.

### Western blot analysis

The protein concentrations of brain tissues were determined by a BCA kit (Beyotime, Shanghai, China). Different samples with an equal amount of protein were separated on 10% or 12% sodium dodecyl sulfate–polyacrylamide gel electrophoresis (SDS-PAGE) and then transferred to polyvinylidene fluoride (PVDF) membranes. After blocking with 5% skim milk for 1 h at room temperature, the membranes were incubated with the primary antibodies overnight at 4 °C. To reduce the amount of primary antibodies used, we cut the membranes to the appropriate size based on the marker corresponding to the molecular weight of the protein before incubating the membranes with the following primary antibody. Primary antibodies: a anti-NKG2D (1:1000, Santa Cruz Biotechnology, Santa Cruz, CA, USA), anti-H60 (1:500, R&D systems), anti-Rae-1 (1:1000, R&D systems), anti-DAP10 (1:200, SantaCruz), anti-β-actin (1:5000; ZSGB-BIO, Beijing, China), anti-NF-κB p65 (1:200, HUABIO) and anti-Histone H3 (1:5000, HUABIO). After that, the membranes were incubated with corresponding secondary antibodies for 1 h at 37°C. The blots were visualized by ECL reagent and then captured using the Chemi-Doc™XRS + imaging system (Bio-Rad, Hercules, California, USA) (Supplementary information).

### Enzyme-linked immunosorbent assay (ELISA)

The levels of TNF-α and IL-6 in brain tissues were determined with ELISA kits (Dakewe Biotech, Beijing, China) in accordance with the manufacturer’s instructions.

### Statistical analysis

All data were expressed as the mean ± standard error of mean (SEM) and were analyzed by SPSS 26.0. The comparisons between the two groups were analyzed by a two-tailed Student’s t-test. The remaining data were made with a one-way analysis of variance (ANOVA). *P* < 0.05 was considered statistically significant.

### Ethics approval and consent to participate

All methods were carried out in accordance with relevant guidelines and regulations. The animal experiments were carried out in compliance with the ARRIVE guidelines, and approved by the Animal Experimentation Ethics Committee of Sichuan University (K2022008).

## Results

### NKG2D expression was increased in neonatal mice after hypoxia and ischemia

**Figure 1:**
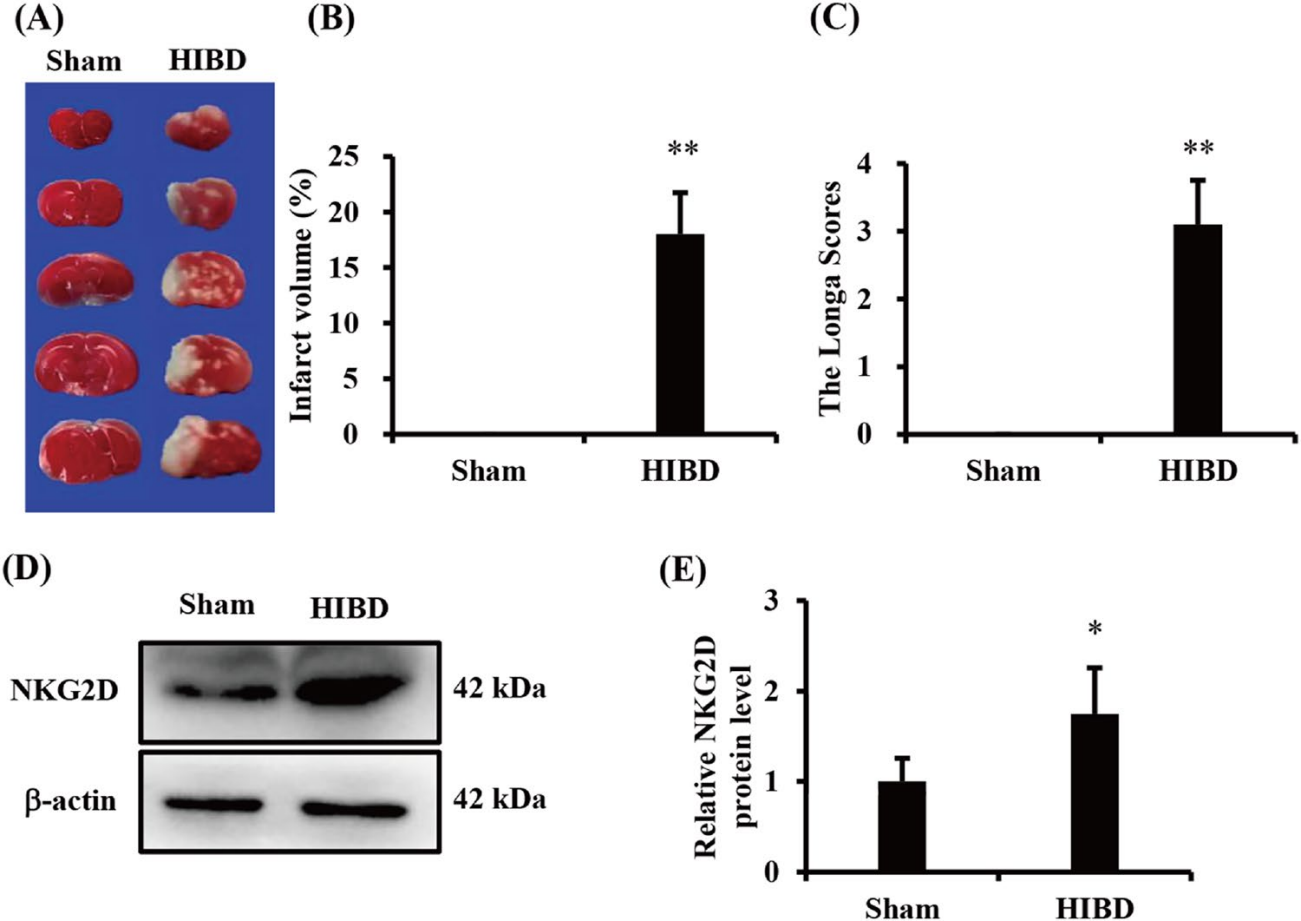
NKG2D was induced by HIBD in newborn mice. (**A**) Representative photographs of the infarct volume detected by TTC staining(n = 5/group). (**B**) Cerebral infarct volume statistics. (**C**) The Zea-Longa scores. (**D**) Representative immunoblot of NKG2D expression in the brain in neonatal mice (n = 6/group). (**E**) Quantitative analysis of NKG2D in brain in neonatal mice. The level of NKG2D was normalized to β-actin. The data are expressed as the mean ± SEM. *p < 0.05 and **p < 0.01 (two-tailed Student’s t-test for comparisons between two groups).

To investigate changes in NKG2D expression in hypoxia and ischemia brain, we established a neonatal mouse model of HIBD using permanent ligation of the left common carotid artery combined with hypoxia. We used TTC staining and the Zea-Longa score to detect cerebral infarct volume and neurological function, respectively. The results showed that hypoxia and ischemia significantly increased the cerebral infarct volume and neurobehavioral scores in mice (Fig. 1A–C), indicating that the HIBD model was successfully established. Moreover, protein levels of NKG2D in hypoxia and ischemia brain significantly increased (Fig. 1D and E). These findings suggest that NKG2D participates in neonatal HIBD.

### NKG2D knockdown attenuated HIBD in neonatal mice

**Figure 2:**
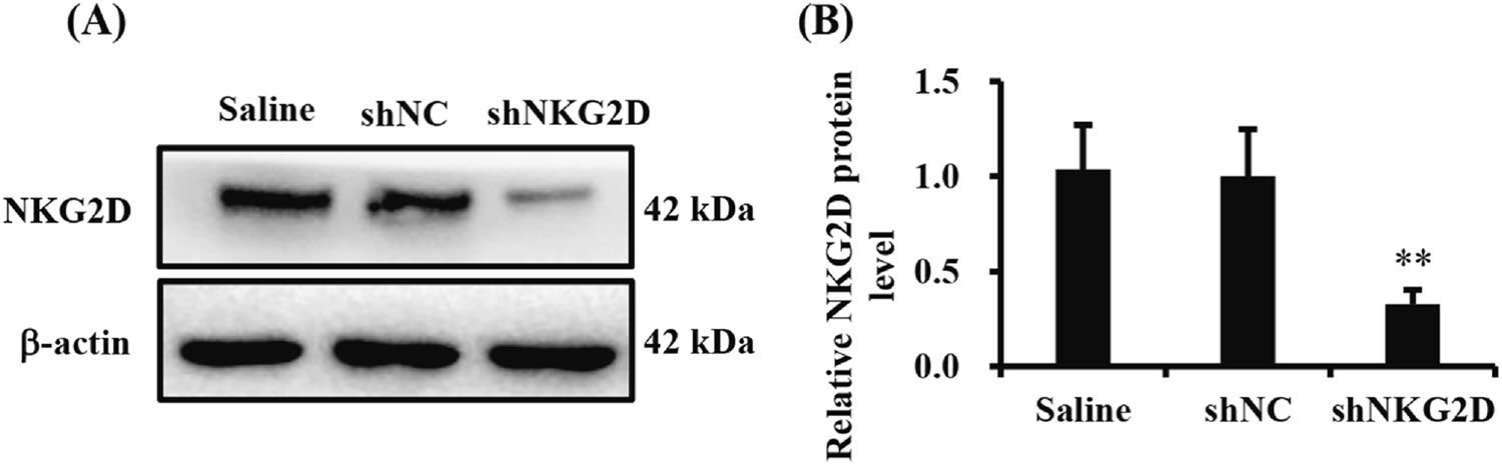
shNKG2D decreased the protein expression of NKG2D in the brain in mice. (**A**) Representative immunoblot of NKG2D in brain in mice. (**B**) Quantitative analysis of the ratio of NKG2D to β-actin. The data was expressed as mean ± SEM (n = 5/group). **p < 0.01 (one-way ANOVA for comparisons between three groups).

**Figure 3:**
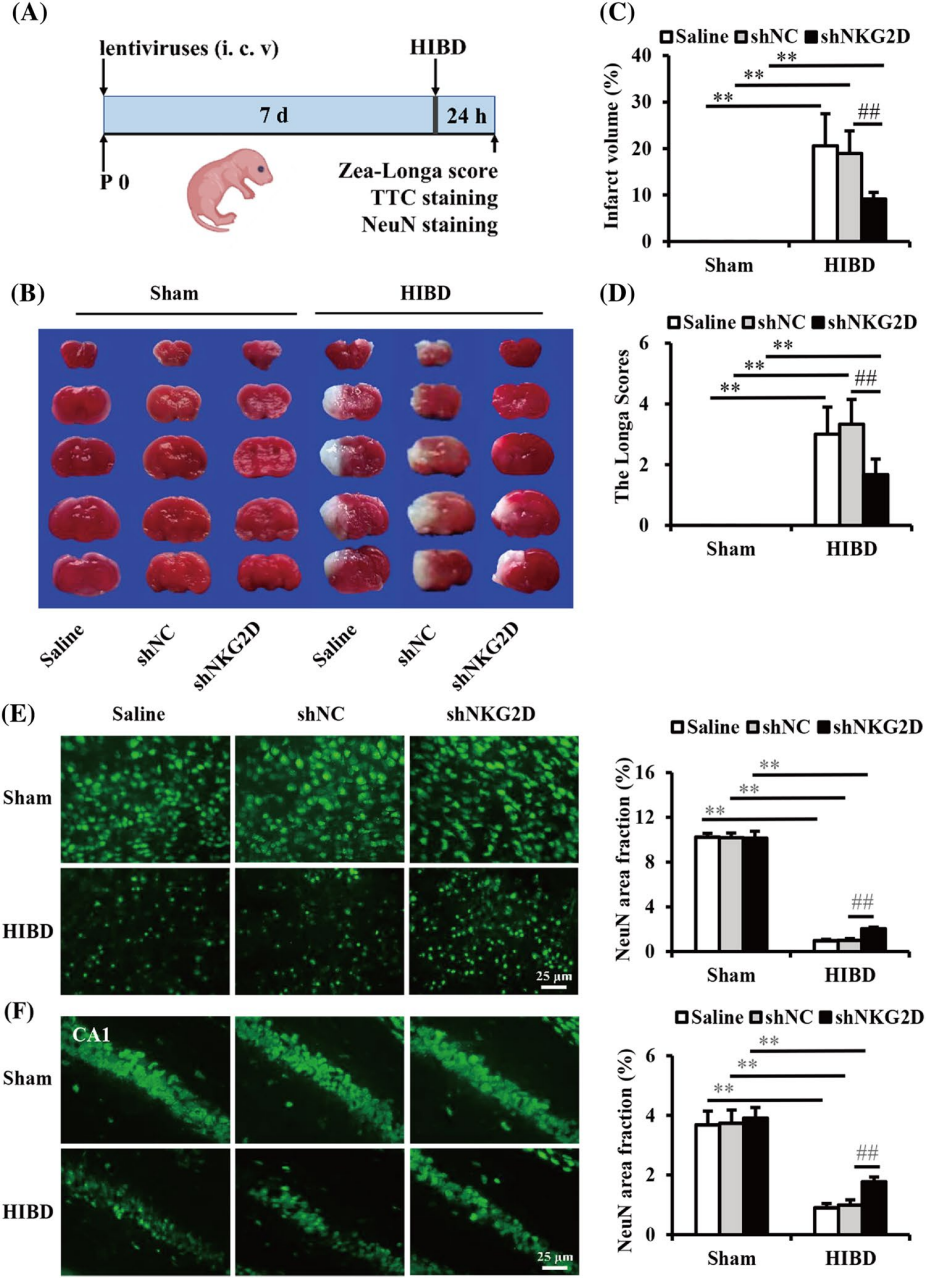
NKG2D knockdown improved HIBD in neonatal mice. (**A**) Experimental schedule of in vivo study. (**B**) Representative photographs of the infarct volume detected by TTC staining. (**C**) Cerebral infarct volume statistics. (**D**) Zea-Longa scores. (E, F) Representative images and analysis of NeuN-positive neurons in the cortex and hippocampal CA1 subregions. The data are expressed as the mean ± SEM (n = 6/group). **p < 0.01 and ##*p* < 0.01 (two-tailed Student’s t-test for comparisons between two groups).

We successfully downregulated NKG2D expression in brain in mice by using a stereotaxic technique and lentiviral system, and the knockdown efficiency was subsequently confirmed with western blot (Fig. 2A,B). To investigate the effect of NKG2D knockdown on HIBD in neonatal mice, either shNKG2D or shNC was intracerebroventricularly (i.c.v.) injected into the left lateral ventricles in 1-day-old mouse pups (Fig. 3A). Seven days after lentivirus administration, the neonatal mice were subjected to HIBD. Protective effects of NKG2D knockdown were evaluated 24 h after HIBD. TTC staining revealed that the infarct size, which increased after HIBD, was significantly decreased by shNKG2D administration (Fig. 3B,C). We also found that NKG2D knockdown significantly decreased Zea-Longa scores in the HIBD group (Fig. 3D). Neurons are the basic units of structure and function in the central nervous system. Numerous studies have shown that neuronal damage in the cerebral cortex and hippocampus CA1 subregions was particularly pronounced in neonatal HIBD mice^25^. As shown in Fig. 3E and F, compared with the Sham group, the number of NeuN-positive neurons in the cerebral cortex and hippocampal CA1 area decreased significantly in HIBD mice, and NKG2D knockdown alleviated HIBD-induced neuronal loss. These results showed that NKG2D knockdown effectively alleviated HIBD in neonatal mice.

### NKG2D knockdown alleviated neuroinflammation in neonatal mice with HIBD

**Figure 4:**
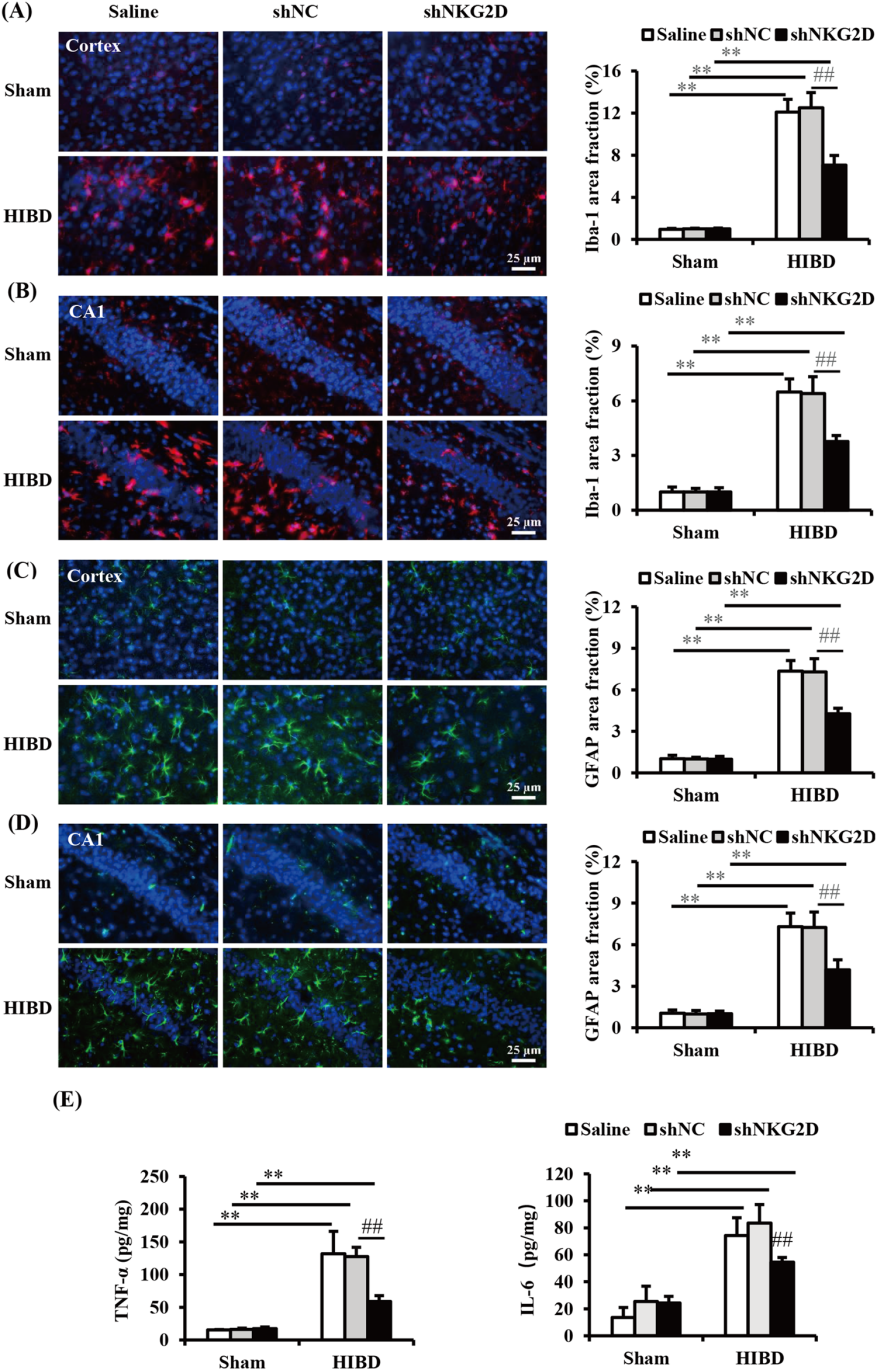
NKG2D knockdown alleviated neuroinflammation in neonatal HIBD mice. (**A**, **B**) Representative images and analysis of Iba-1-positive microglia in the cortex and hippocampal CA1 subregions. (**C**, **D**) Representative images and analysis of GFAP-positive astrocytes in the cortex and hippocampal CA1 subregions. (**E**) Expression levels of TNF-α and IL-6 were measured by ELISA. The data are expressed as the mean ± SEM (n = 6/group). **p < 0.01 and ##*p* < 0.01 (two-tailed Student’s t-test for comparisons between two groups).

In addition to primary brain damage that is caused by hypoxia and ischemia, overactivation of the inflammatory response can trigger secondary brain damage in neonatal HIBD mice. To further explore the role of NKG2D in neuroinflammation in neonatal HIBD mice, we detected the activation of microglia and astrocytes and the release of proinflammatory mediators in the brain of neonatal mice. Iba-1 (Ionized calcium-binding adaptor molecule 1, a marker of microglia) and GFAP (glial fibrillary acidic protein, a marker of astrocytes) were detected by immunofluorescent staining. The expressions of Iba-1 and GFAP significantly increased in the cortex and hippocampal CA1 area in HIBD mice (Fig. 4A–D), and TNF-α and IL-6 significantly increased in the brain in HIBD mice (Fig. 4E). However, NKG2D knockdown inhibited the overactivation of glial cells and expression of proinflammatory mediators (Fig. 4A–E).

**Figure 5:**
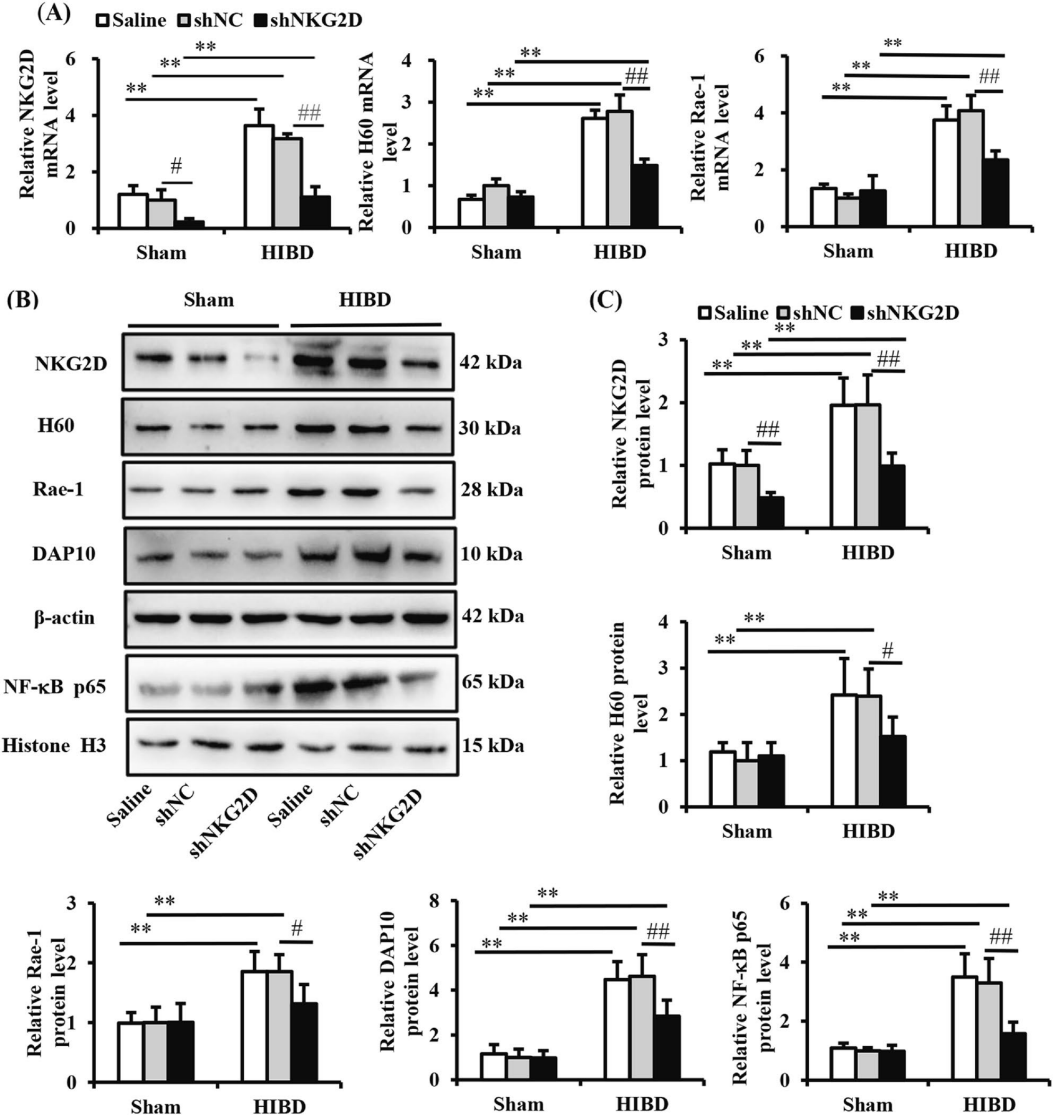
NKG2D knockdown inhibited the NF-κB pathway in the brain in neonatal HIBD mice. (**A**) Quantification of mRNA levels of H60, Rae-1, and NKG2D in neonatal mice. (**B**) Representative immunoblots of NKG2D, H60, Rae-1, and DAP10 expression and translocation of NF-κB p65 in neonatal mice. (**C**–**G**) Quantitative analysis of NKG2D, H60, Rae-1, DAP10, and NF-κB p65. Levels of NKG2D, H60, Rae-1, and DAP10 were normalized to β-actin, and the level of NF-κB p65 was normalized to histone H3. The data are expressed as the mean ± SEM (n = 6/group). **p < 0.01; #*p* < 0.05, ##*p* < 0.01 (two-tailed Student’s t-test for comparisons between two groups).

To confirm the mechanism of NKG2D in neuroinflammation induced by hypoxia and ischemia, we examined the expression of NKG2D, H60, Rae-1, DAP10, and the nuclear translocation of NF-κB p65, respectively. As shown in Fig. 5, levels of NKG2D, H60, Rae-1 and DAP10 as well as the nuclear translocation of NF-κB p65 significantly increased in the ischemic brain in HIBD mice. Compared with the shNC/HIBD group, the expression of H60, Rae-1 and DAP10 as well as the nuclear translocation of NF-κB p65 significantly decreased in the shNKG2D/HIBD group. These results indicated that NKG2D knockdown attenuated hypoxia and ischemia-induced neuroinflammation by inhibiting the NKG2D/NKG2DLs axis and NF-κB nuclear translocation in the brain in HIBD mice.

## Discussion

Neuroinflammation has been demonstrated to be one of the major pathogenic causes of secondary brain damage, affecting the prognosis of hypoxic-ischemic newborns^26^. The present study found that hypoxia-ischemia significantly increased the expression of NKG2D in the brain tissues of neonatal mice, while NKG2D knockdown by shRNA lentivirus i.c.v. administration significantly attenuated brain infarct volume, activation of the NKG2D/NKG2DLs axis and NF-κB, and neuroinflammation after cerebral hypoxia-ischemia in neonatal mice. These findings provide evidence that the NKG2D signaling pathway contributes to HIBD in neonatal mice, suggesting a potential treatment target for HIBD.

Activation of the NKG2D receptor is one of the most direct mechanisms by which the immune system recognizes stress cells, which plays an important role in initiating and maintaining inflammation. In chronic inflammatory diseases, such as deformable arthritis, diabetes mellitus type 1, and atherosclerosis, the expression of NKG2DLs is usually upregulated, and these ligands subsequently activate lymphocytes in the innate and adaptive immune systems through NKG2D receptors^27,28^. Based on the regulatory role of NKG2D/NKG2DLs in inflammation, NKG2D antibodies effectively block the interaction between NKG2D and its ligands with high targeting specificity^27–29^. They are often used as an intervention in NKG2D-dependent inflammatory diseases. Some studies reported that anti-NKG2D antibodies can delay NK cell-mediated allergic airway inflammation, block CD4+T cell-mediated colitis, improve collagen-induced arthritis, and alleviate NKT cell-mediated acute hepatitis^30–33^. However, these therapeutic antibodies have shortcomings, such as a short half-life and limited administration routes. To avoid these problems that are associated with anti-NKG2D antibodies, we used RNA interference (RNAi) to block the binding of NKG2D to NKG2DLs. RNAi is a gene silencing technique that is induced by double-stranded RNA (dsRNA), and it is one of the most advanced and rapidly developing frontiers in gene therapy^34^. The dsRNA is processed into short-interfering RNAs (siRNAs), which guide mRNA degradation in a sequence-specific manner and effectively trigger the silencing of a specific gene after transcription^34,35^. Compared with gene knockout, RNAi-based gene silencing technology has speed and economic advantages. Moreover, with the application of viral vectors, such as lentiviruses and adenoviruses, and the popularization of antibiotic screening and fluorescence sorting, RNAi achieves effective and stable gene silencing in various biological systems. Thus, RNAi is widely used to study gene function and in drug development^36^. In the present study, NKG2D knock-down with lentiviruses using RNAi technology was used to explore the role of NKG2D in neonatal HIBD mice.

In the developing brain, glial cells are the main innate immune cells, accounting for the immune modulation in the brain. They are involved in important actions in the brain, including immune surveillance, the maintenance of environmental stability, and synaptic pruning^12,37^. Cerebral glial activation has been demonstrated to be involved in various CNS diseases such as HIBD^38^. Brain hypoxia-ischemia induces the death of a large number of neurons, resulting in glial activation, production of proinflammatory mediators, and neuroinflammatory response^39^. After destruction of the blood–brain barrier due to HIBD, activated glial cells may release vast proinflammatory mediators and matrix metalloproteinases that can recruit peripheral immune cells (granulocytes and monocytes, etc.) to infiltrate into the brain parenchyma, further aggravating neuroinflammation and brain damage^38^. As an important immune receptor, NKG2D may combine with various ligands, and recruit p85 and Grb2 through the adaptor protein DAP10, leading to activation of the PI3K-Akt, Grb2-Vav1-SOS1, and NF-κB signaling pathways and production of pro-inflammatory mediators^16^. Studies have reported the role of NKG2D-mediated signaling pathway in tumor immunity and inflammation, including tumor immune monitoring and immune evasion^40^. In recent, the potential role of the NKG2D signaling pathway in other inflammatory diseases has gradually attracted attention.

The classic Rice-Vannucci method is widely used to simulate neonatal HIBD because of significant infarction and white matter damage in the cerebral cortex and hippocampus that is caused by additional hypoxic processing after permanent ischemia^21,41^. In the present study, we permanently ligated the left common carotid artery combined with hypoxia in neonatal mice to establish a mice model of neonatal HIBD and explore the role of NKG2D in HIBD. Notably, we found for the first time that both mRNA and protein levels of NKG2D were greatly increased in the developing brain of neonatal mice after HIBD. NKG2D knockdown by using lentivirus exerted a significant neuroprotective effect against HIBD in neonatal mice, as indicated by decreases in the infarcted volume and the neurobehavioral deficits, and an increase in NeuN-positive neuronal cells. In addition, NKG2DLs are known to be seldomly expressed in normal cells under the physiological condition, but NKG2DLs expression could be significantly upregulated in the immunopathological conditions such as malignant or infected^18^. In the present study, we observed significant increases in both mRNA and protein levels of two NKG2DLs, H60 and Rae-1, in the brain of neonatal mice after HIBD. Interestingly, we found that NKG2D knockdown by using lentivirus mediated significant decreases in NKG2DLs (H60 and Rae-1), which was probably attributed to the attenuation of HIBD through the neuroprotective effect of NKG2D knockdown. Studies have indicated wide differences in the expression profile of various NKG2DLs in various cells. In response to cell stress, for example, H60 was induced on T cells, macrophages, and dendritic cells, whereas Rae-1 were mainly induced on dendritic cells^31^. To further reveal the potential role of NKG2DLs as the damage-associated molecular patterns in HIBD, therefore, the next research is need to explore the expression profiles of different NKG2DLs in different cell types of brain.

In the present study, NKG2D knockdown significantly attenuated the activation of microglia and astrocytes after HIBD. Meanwhile, we observed the significant increases in the expression of adaptor molecule DAP10 of NKG2D, the nuclear translocation of NF-κB p65, and the levels of proinflammatory cytokines (TNF-α and IL-6) in the brain of neonatal mice with HIBD, which could be significantly decreased by NKG2D knockdown. These results demonstrated that NKG2D is involved in the neuroinflammation after HIBD in neonatal mice through induction DAP10 expression and NF-κB activation.

## Conclusion

In conclusion, the current findings show for the first time that the NKG2D/NKG2DLs axis is activated after cerebral hypoxia-ischemia, and NKG2D knockdown may exert anti-inflammatory and neuroprotective effects in the neonatal mice with HIBD through downregulation of NKG2D/NKG2DLs/DAP10/NF-κB signaling pathway. These results suggest that NKG2D may be a potential target for the treatment of neonatal HIBD.

### Supplementary Information

Supplementary Information.

Supplementary Information.

## Data Availability

The datasets used and/or analyzed during the present study are available from the corresponding author on reasonable request.

## Abbreviations

HIBD: Hypoxic-ischemic brain damage
NKG2D: Natural killer group 2 member D
NKG2DLs: NKG2D ligands
DAP10: DNAX activating protein 10
NF-κB: Transcription factor nuclear factor kappa-B
Rae-1: Mouse retinoic acid early transcript 1
H60: Histocompatibility antigen 60
TNF-α: Tumor necrosis factor-α
IL-6: Interleukin-6
NK: Natural killer
NKT: Natural killer T
MICA/B: MHC class-I-related chains A or B
ULBP1-6: UL16-binding proteins
CNS: Central nervous system
SDS-PAGE: Sodium dodecyl sulfate–polyacrylamide gel electrophoresis
PVDF: Polyvinylidene fluoride
ELISA: Enzyme-linked immunosorbent assay
SEM: Standard error of mean
ANOVA: Analysis of variance
Iba-1: Ionized calcium-binding adaptor molecule 1
GFAP: Glial fibrillary acidic protein
RNAi: RNA interference
dsRNA: Double-stranded RNA
siRNA: Short-interfering RNA
