# Supplementary material for: NKG2D knockdown improves hypoxic-ischemic brain damage by inhibiting neuroinflammation in neonatal mice
Source: Sci Rep. 2024 Jan 28;14:2326. doi: 10.1038/s41598-024-52780-3 (PMC10822867; doi:10.1038/s41598-024-52780-3)
Supplement: Supplementary file 1 — Supplementary Information. [file 41598_2024_52780_MOESM1_ESM.pdf]

Figure 1 NKG2D was induced by HIBD in newborn mice.

NKG2D

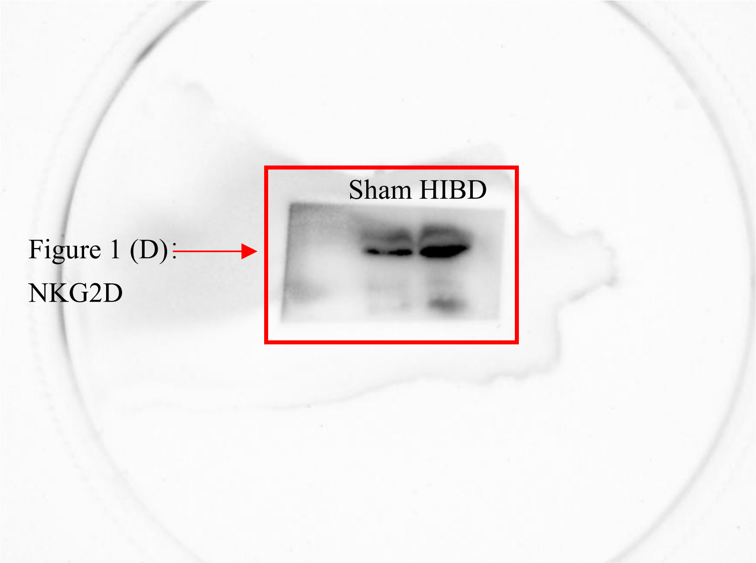

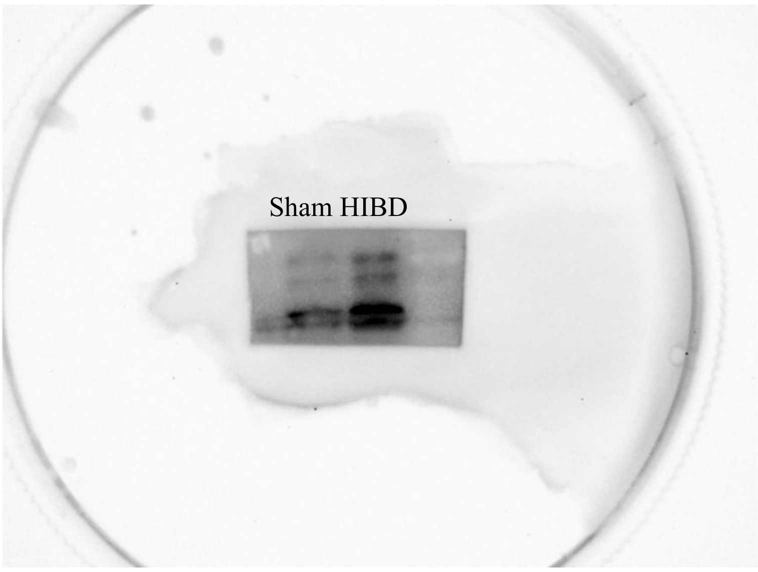

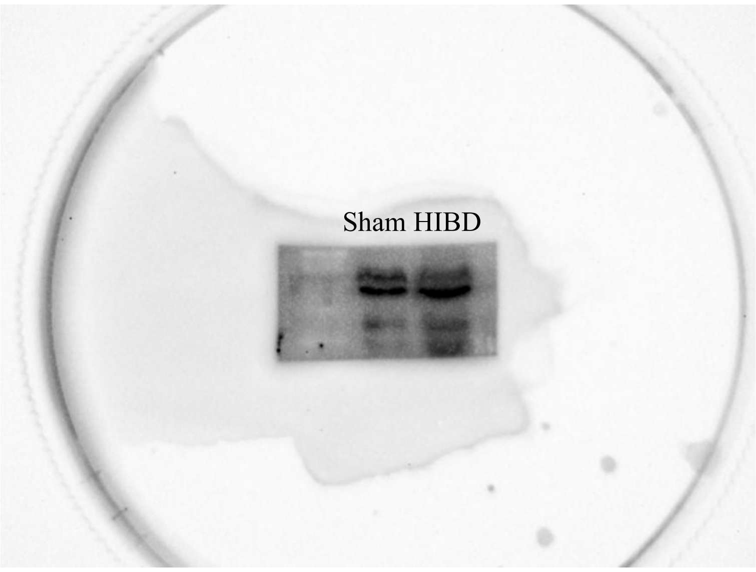
Sham HIBD

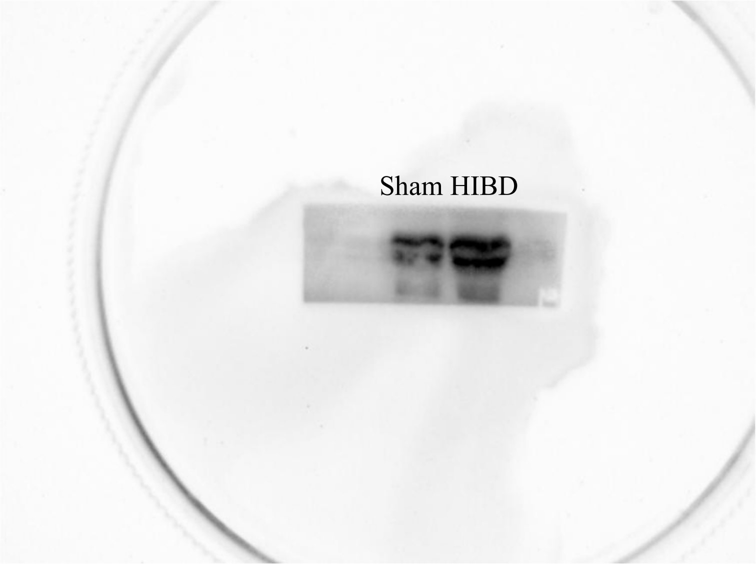
Sham HIBD

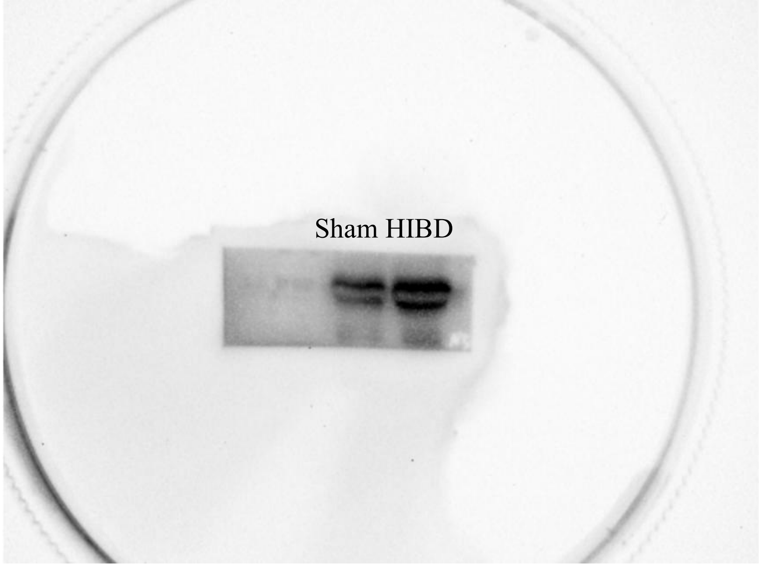
Sham HIBD

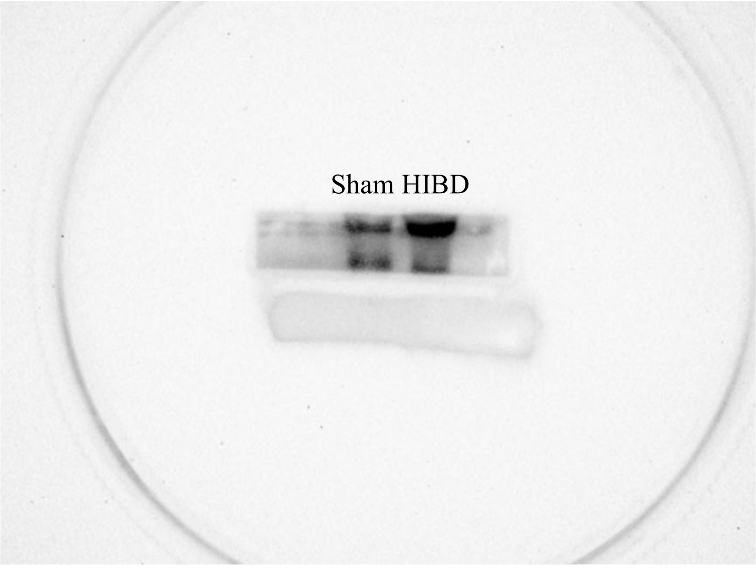

β-actin

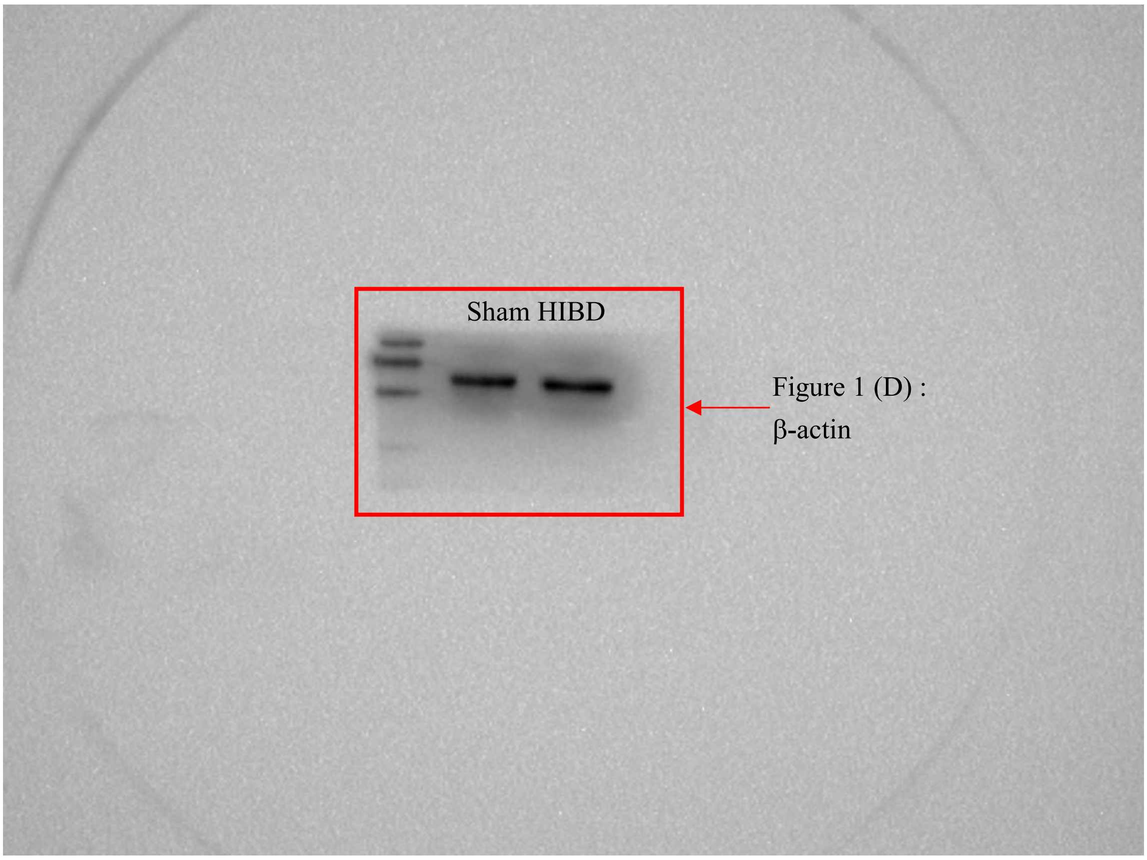

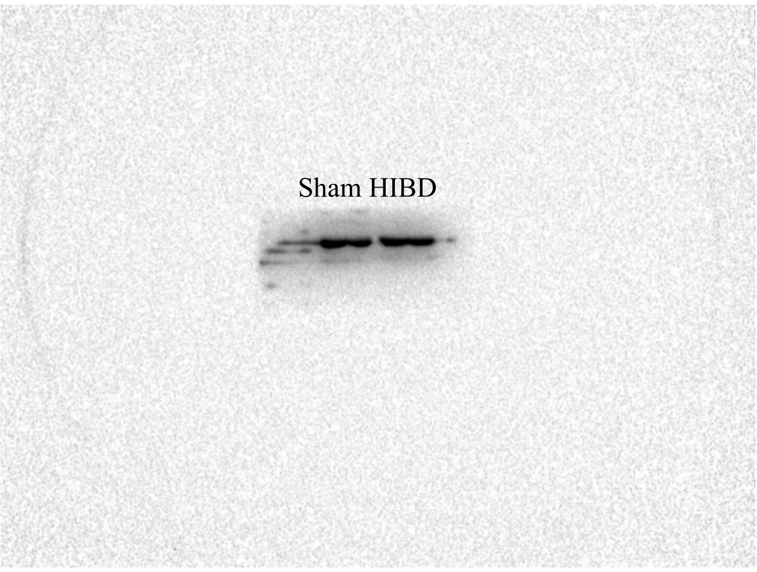
Sham HIBD

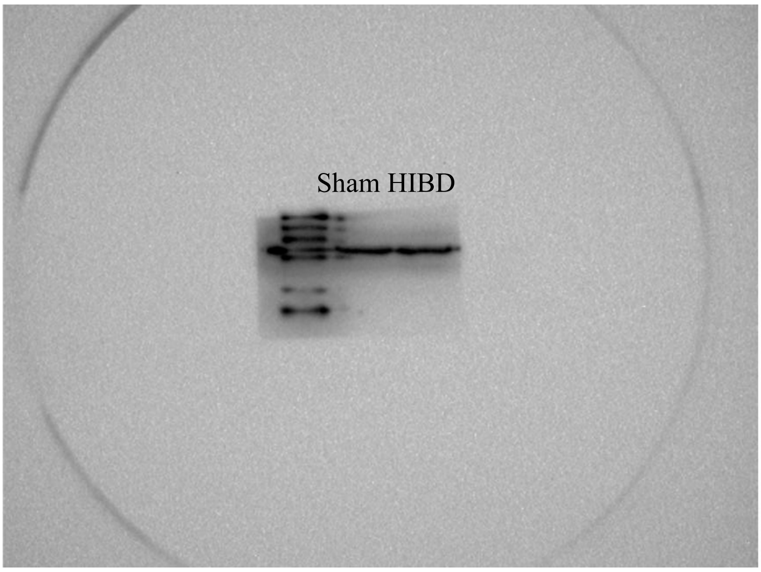
Sham HIBD

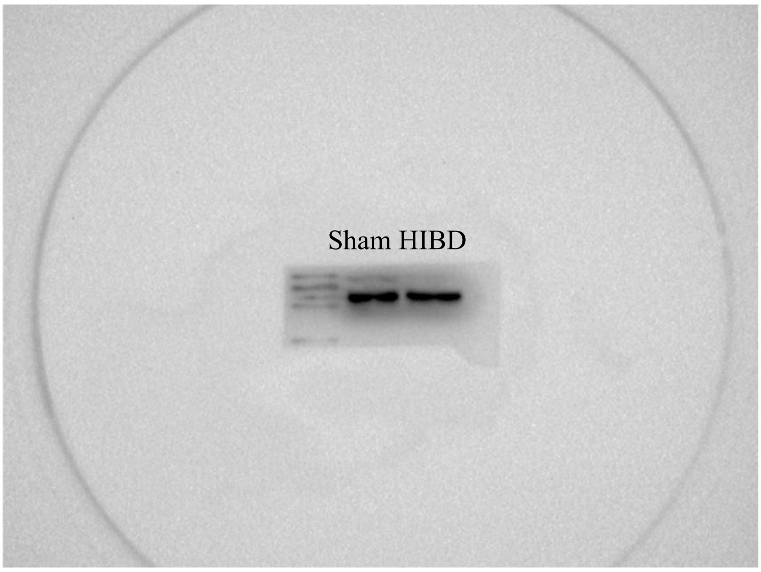
Sham HIBD

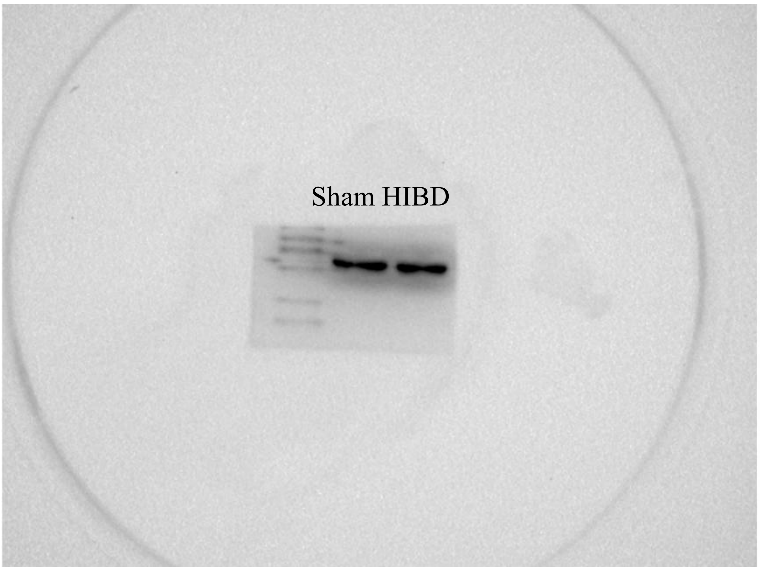
Sham HIBD

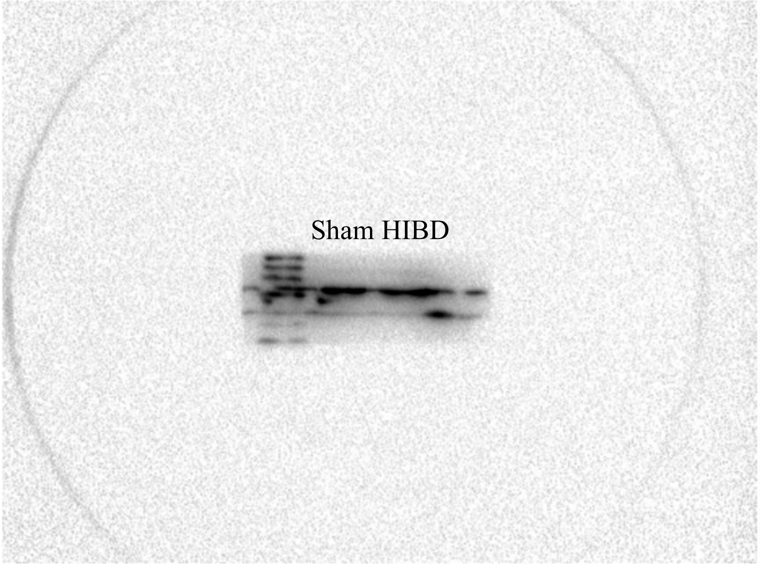
Sham HIBD

Figure 2. shNKG2D decreased the protein expression of NKG2D in the brain in mice.

NKG2D

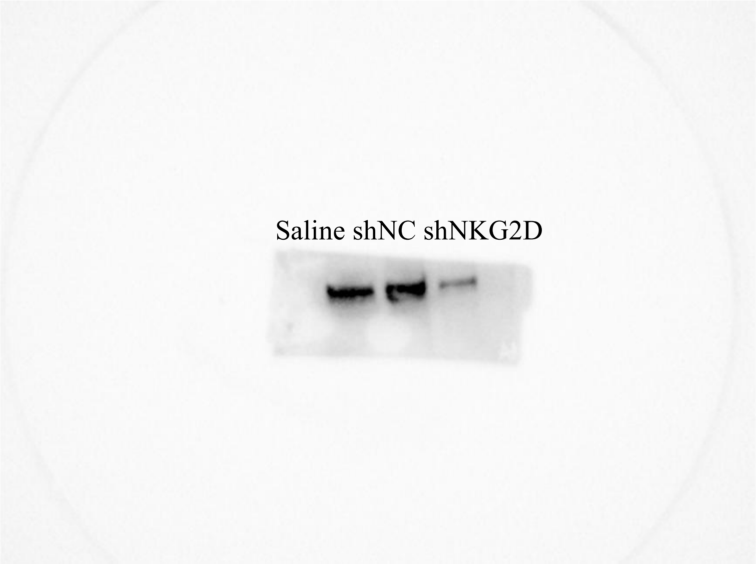

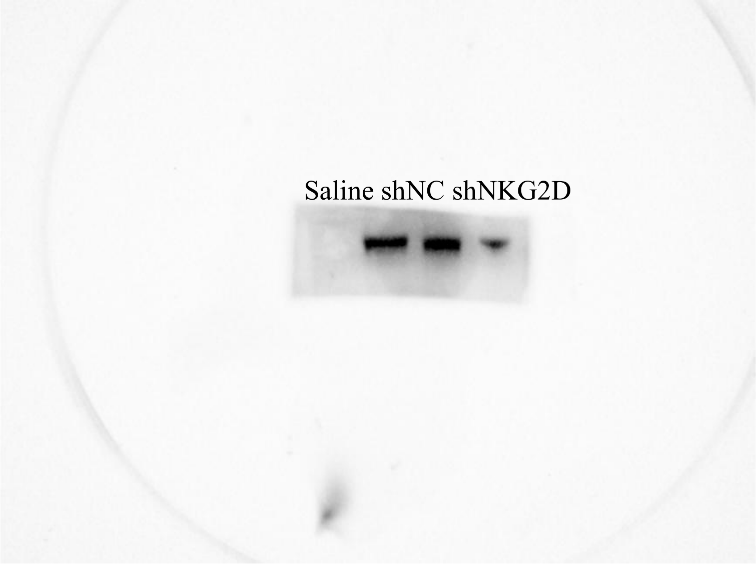

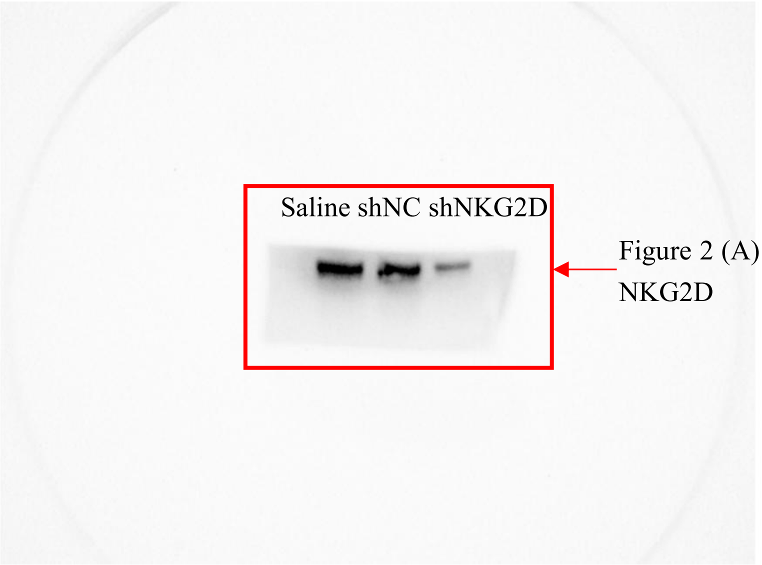

Figure 2 (A): NKG2D

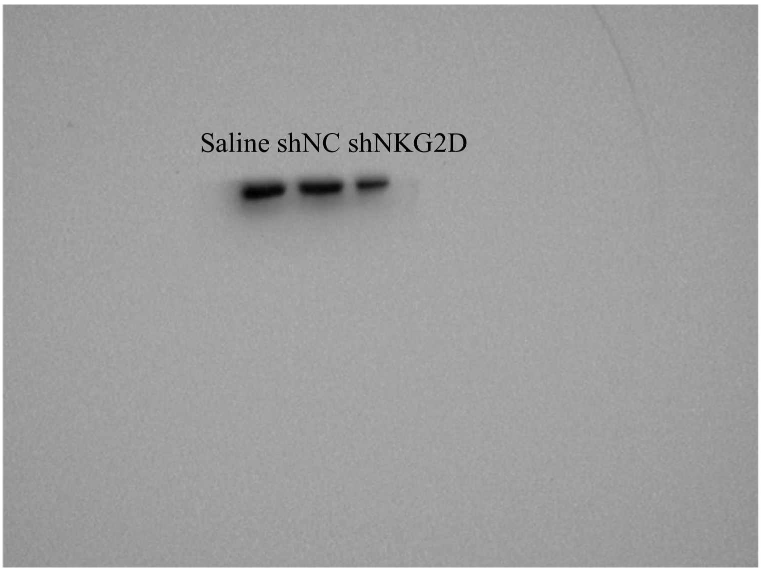

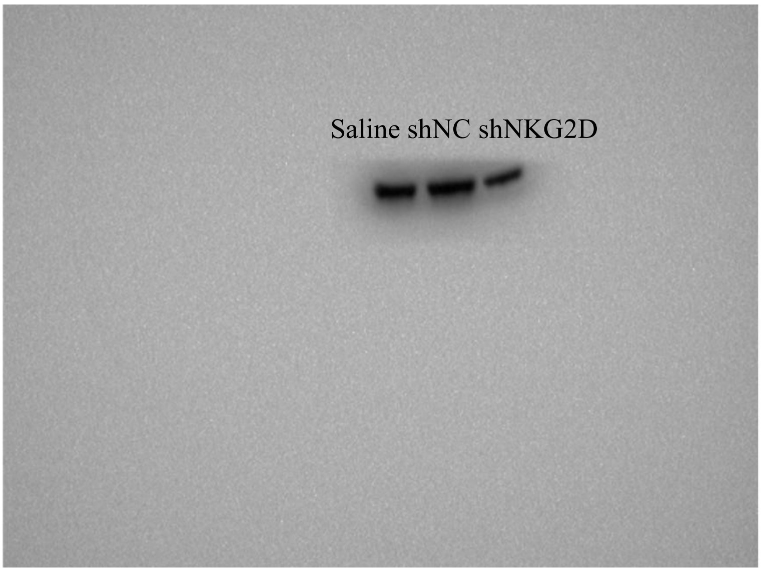

β-actin

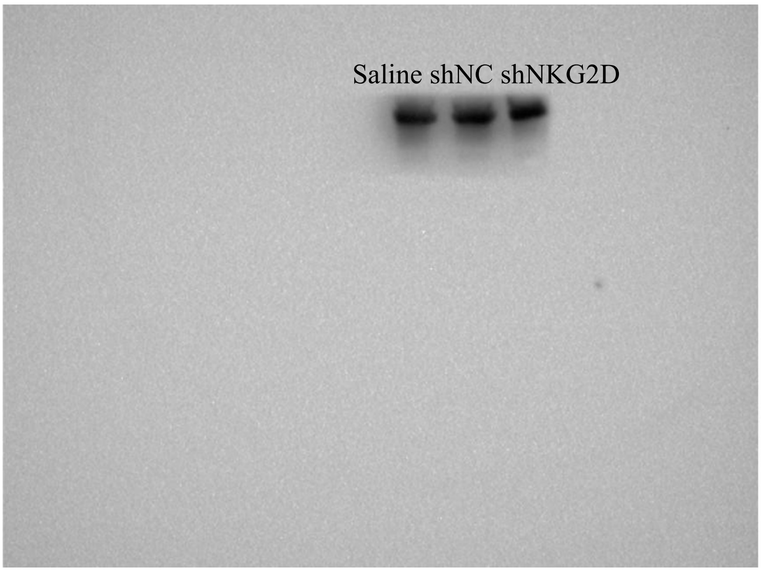

Saline shNC shNKG2D

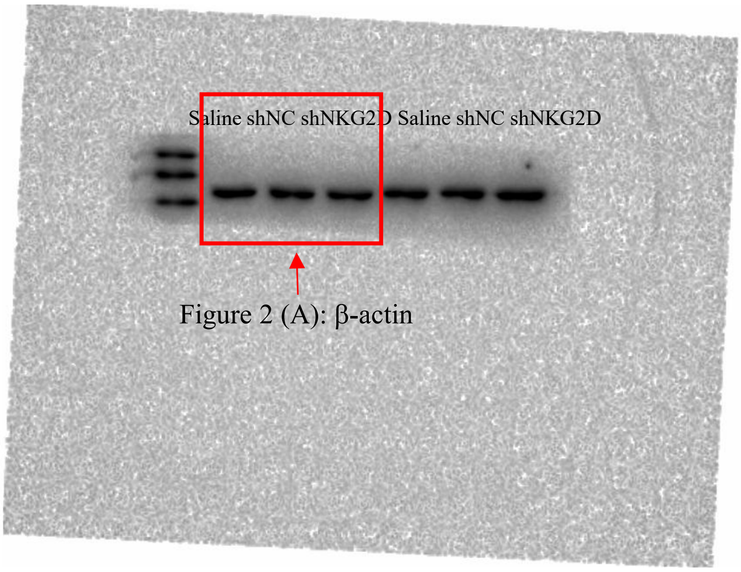

Saline shNC shNKG2D Saline shNC shNKG2D
Figure 2 (A): β-actin

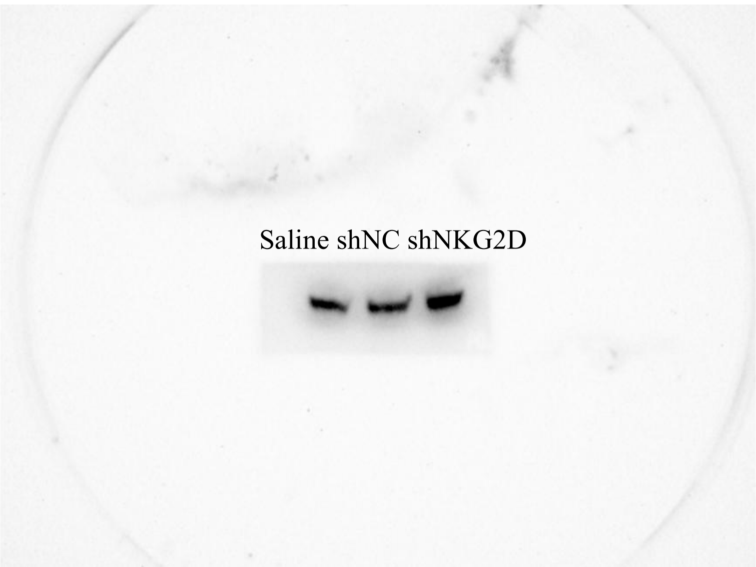

Saline shNC shNKG2D

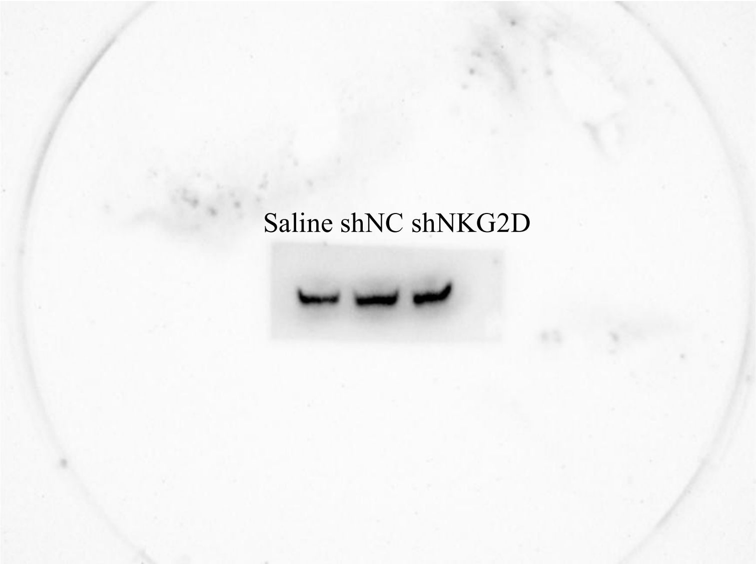
Saline shNC shNKG2D

Figure 5. NKG2D knockdown inhibited the NF-κB pathway in the brain in neonatal HIBD mice.

NKG2D

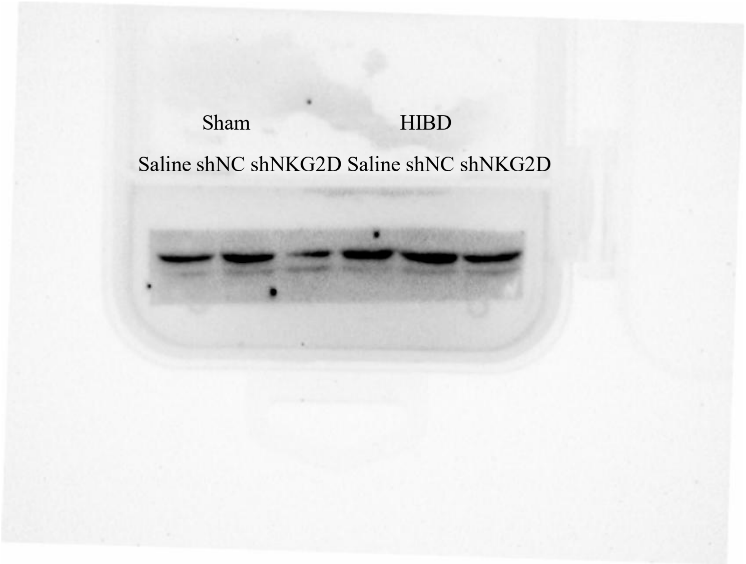

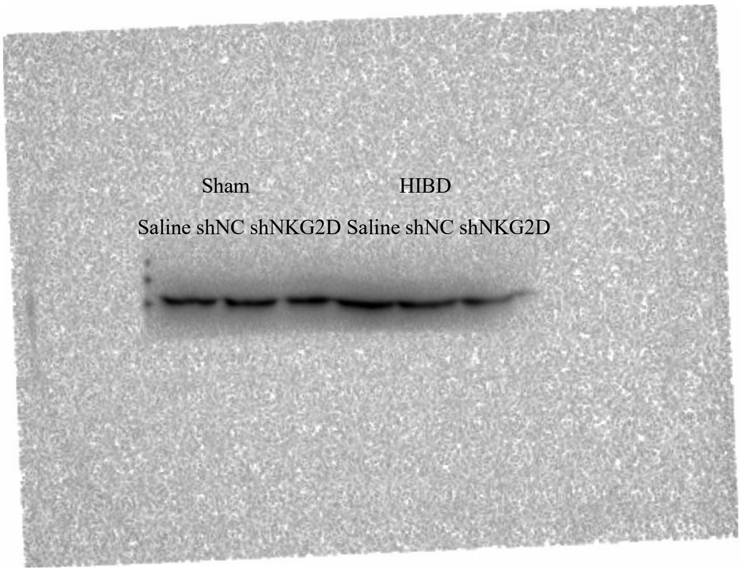

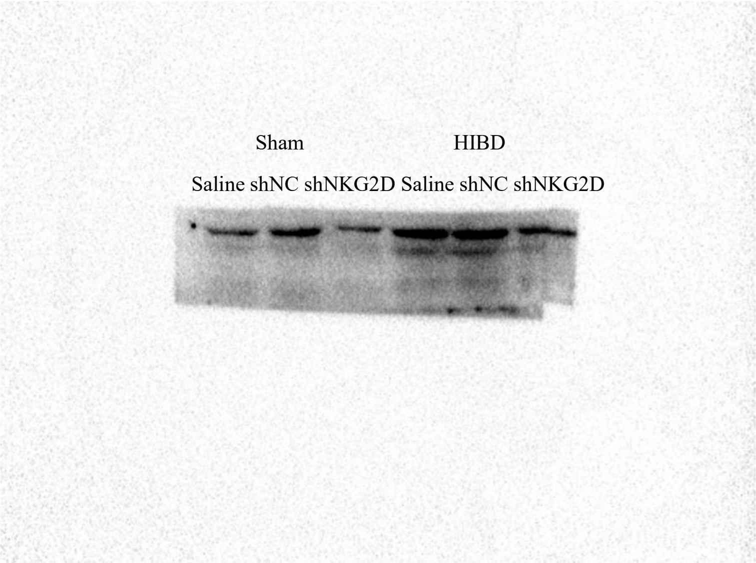

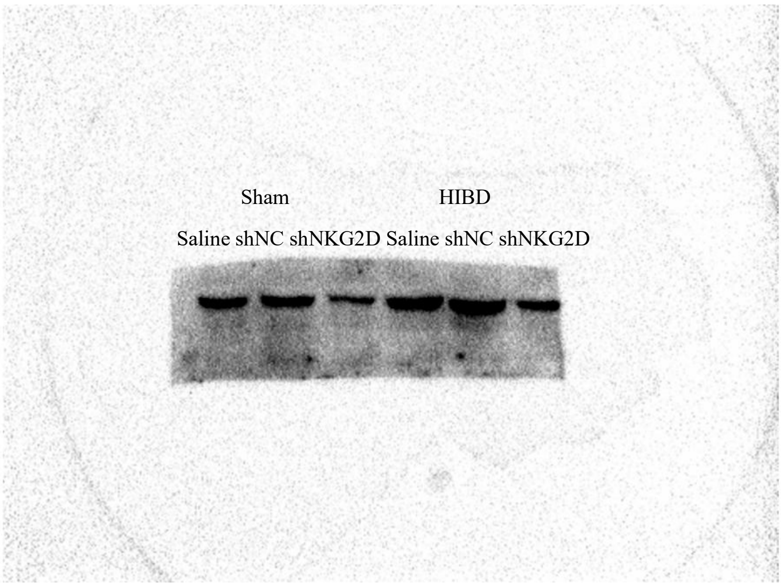

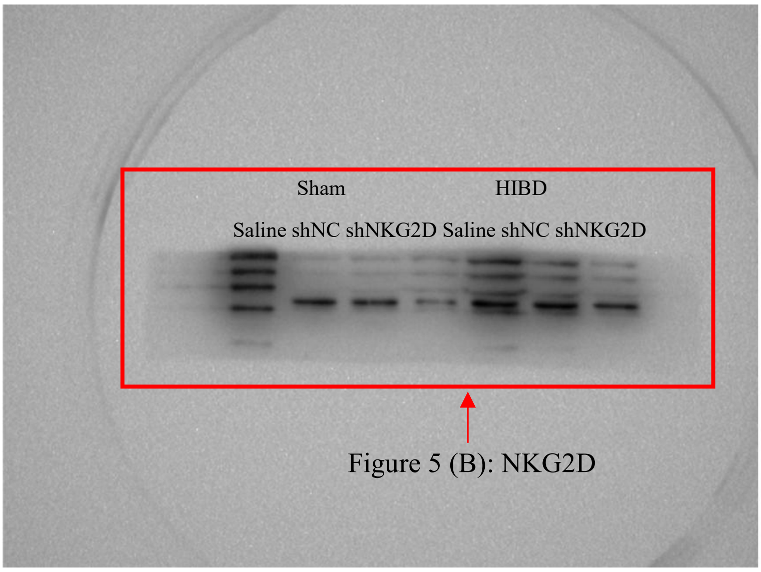

Figure 5 (B): NKG2D

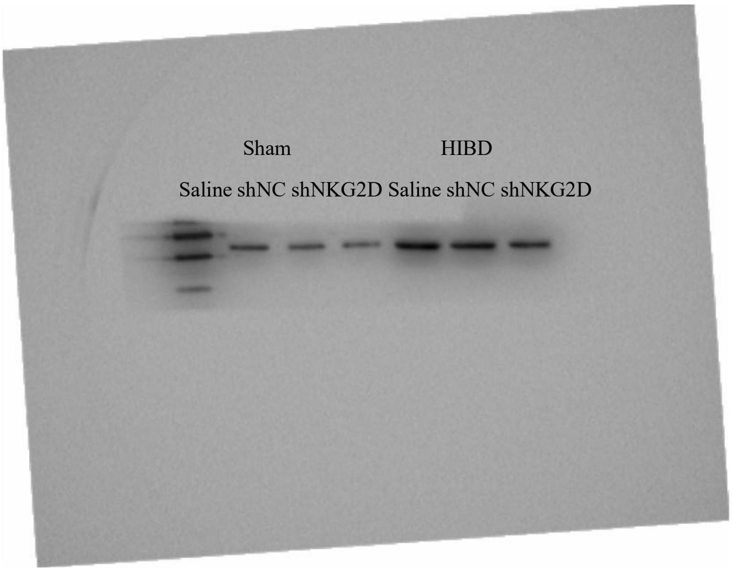
Sham
HIBD
Saline shNC shNKG2D Saline shNC shNKG2D

H60

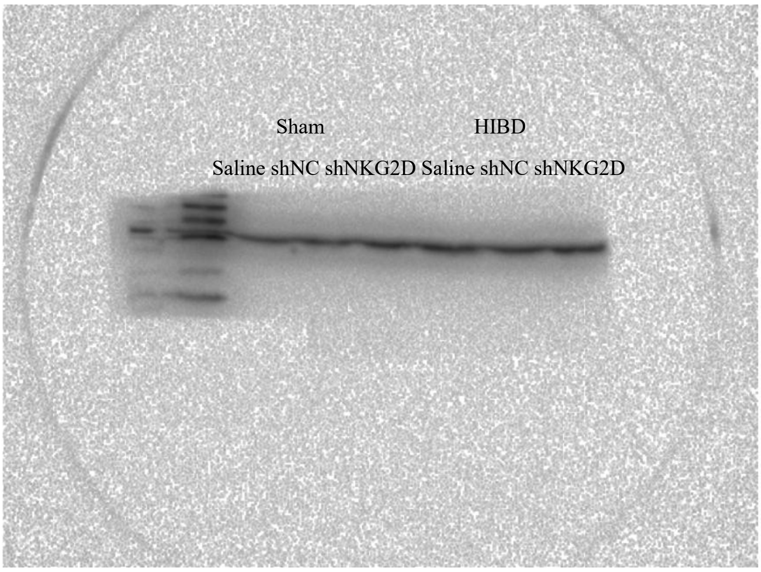
Sham
HIBD
Saline shNC shNKG2D Saline shNC shNKG2D

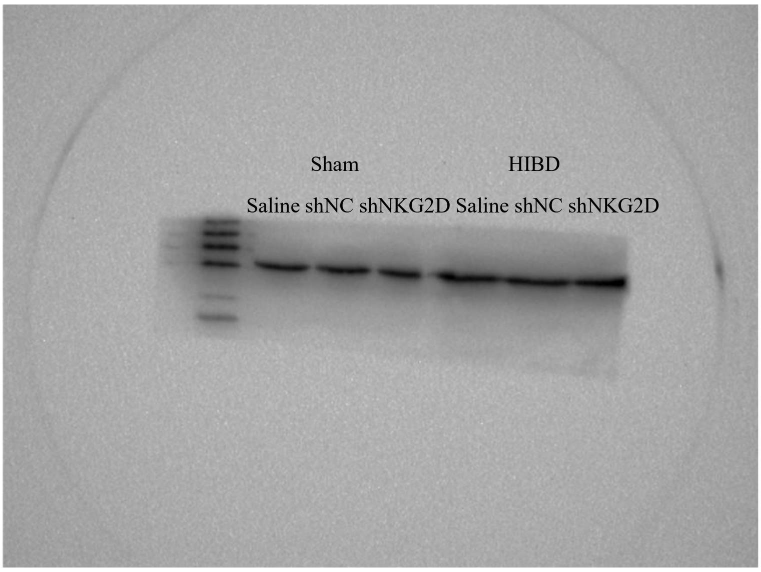
Sham
HIBD
Saline shNC shNKG2D Saline shNC shNKG2D

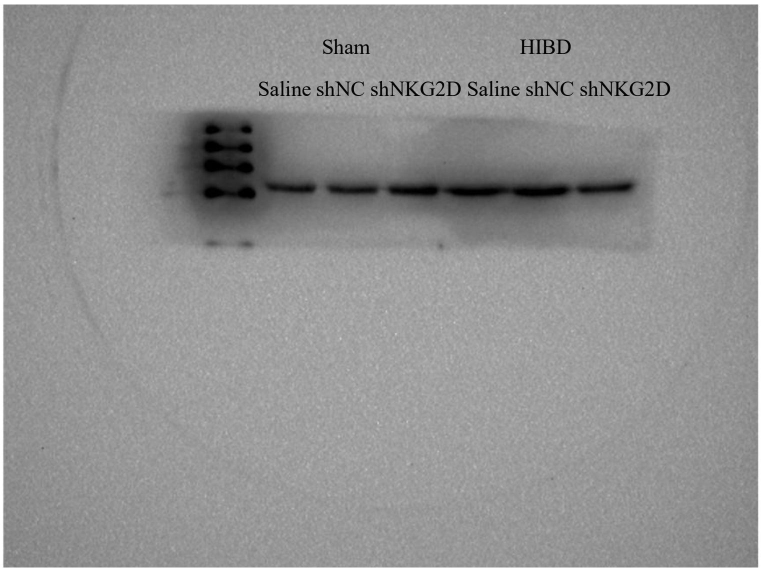
Sham
HIBD
Saline shNC shNKG2D Saline shNC shNKG2D

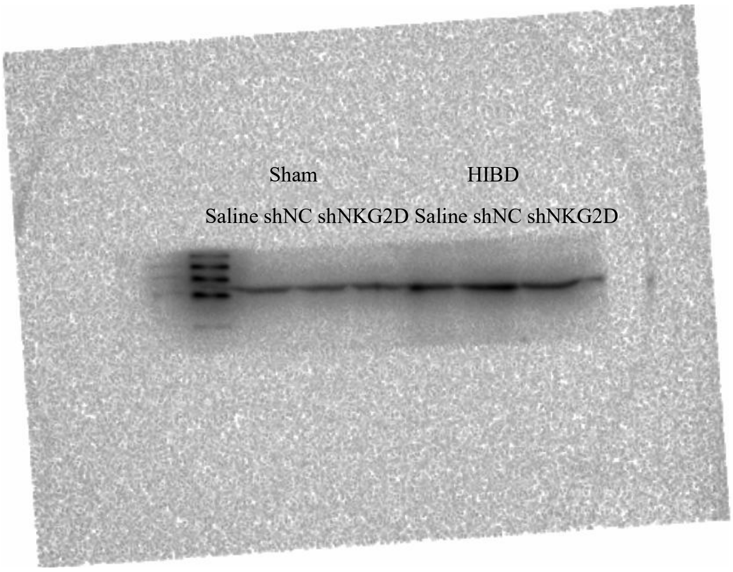

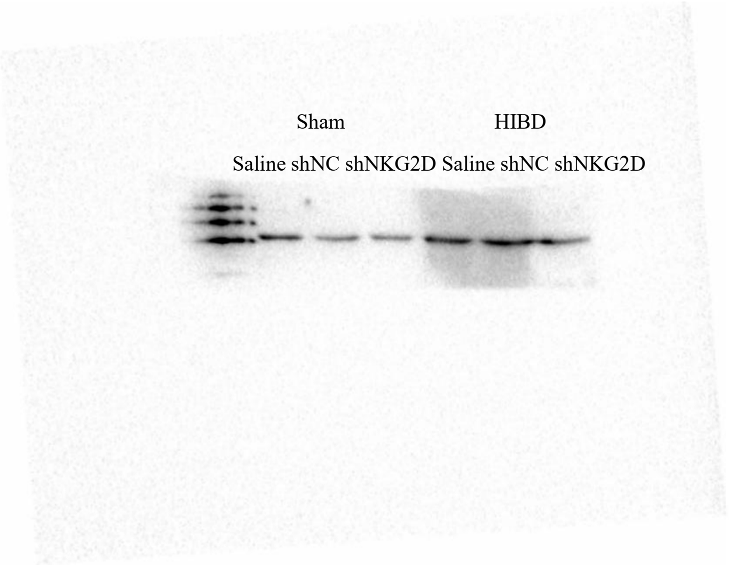

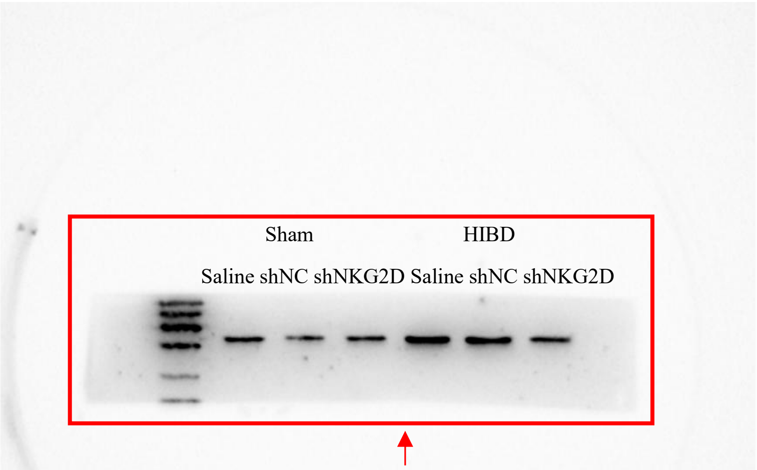

Figure 5 (B): H60

Rae-1

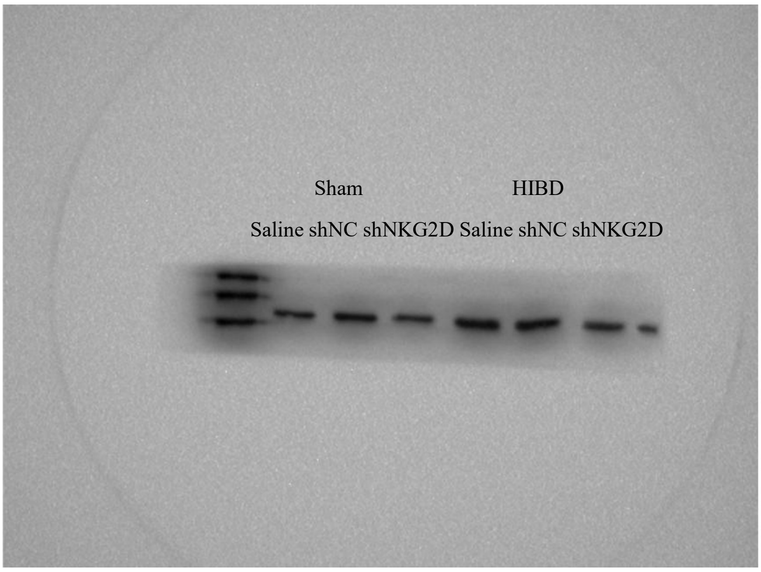
Sham
HIBD
Saline shNC shNKG2D Saline shNC shNKG2D

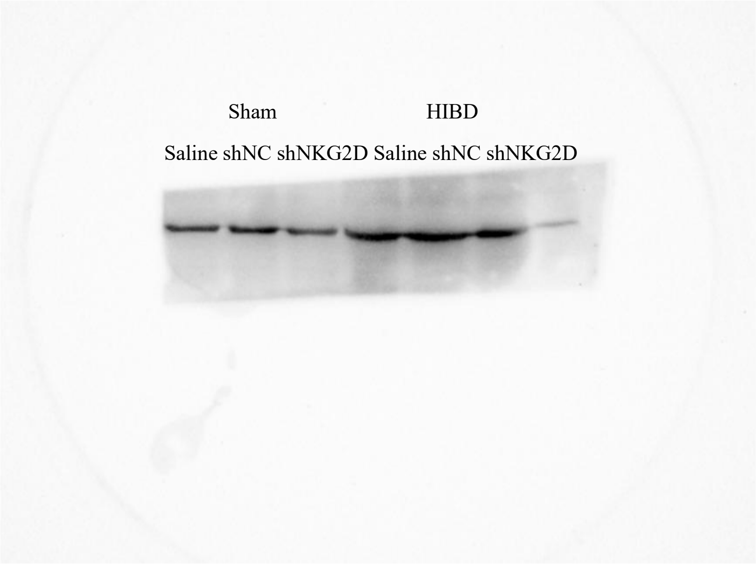
Sham
HIBD
Saline shNC shNKG2D Saline shNC shNKG2D

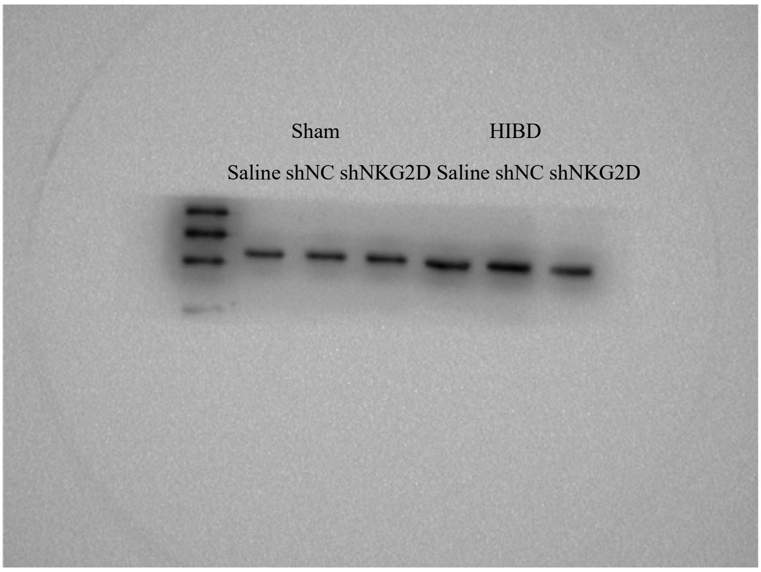
Sham
HIBD
Saline shNC shNKG2D Saline shNC shNKG2D

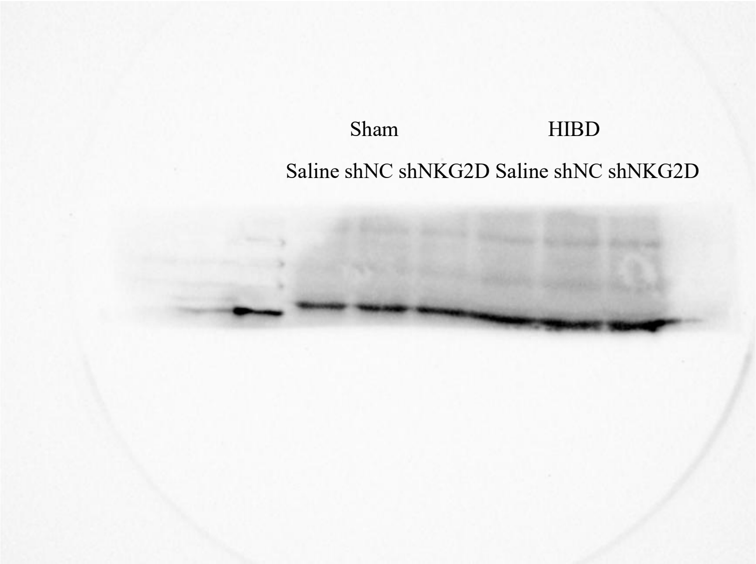

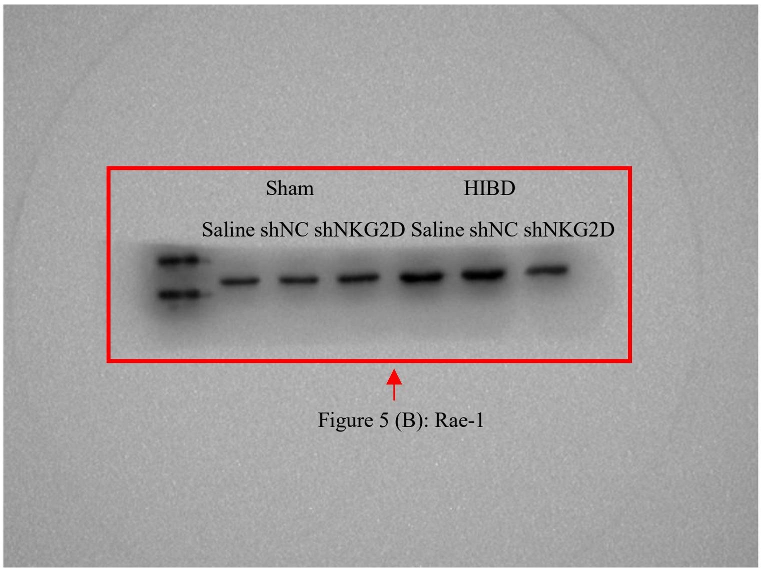

Figure 5 (B): Rae-1

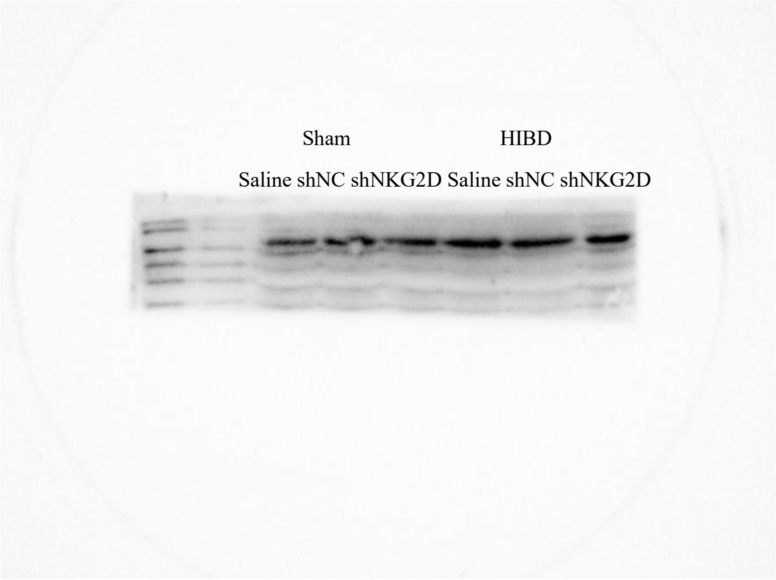

DAP10

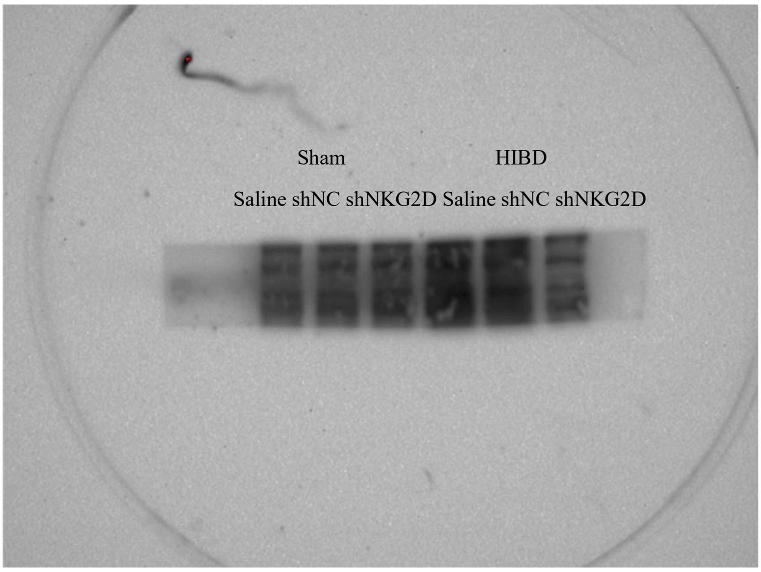
Sham
HIBD
Saline shNC shNKG2D Saline shNC shNKG2D

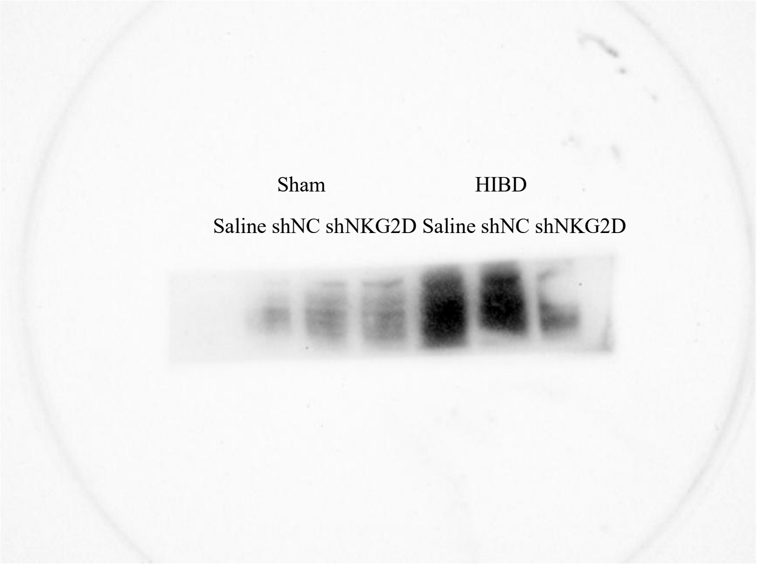
Sham
HIBD
Saline shNC shNKG2D Saline shNC shNKG2D

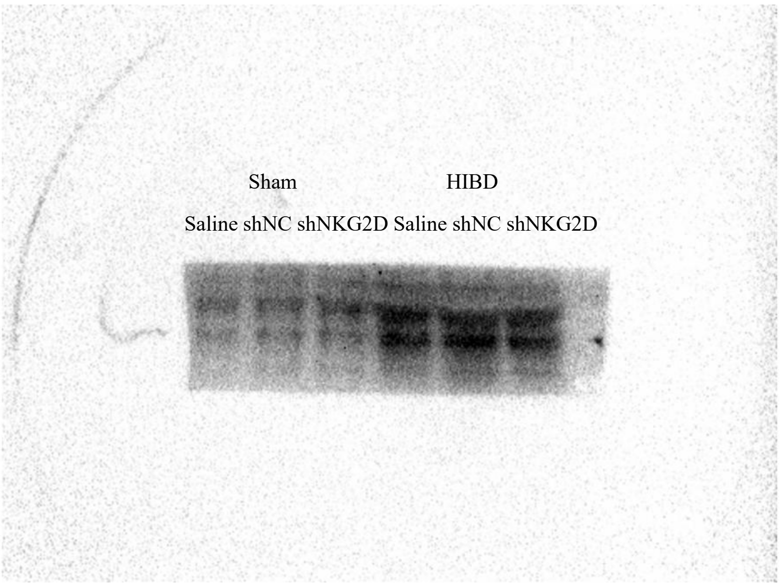
Sham
HIBD
Saline shNC shNKG2D Saline shNC shNKG2D

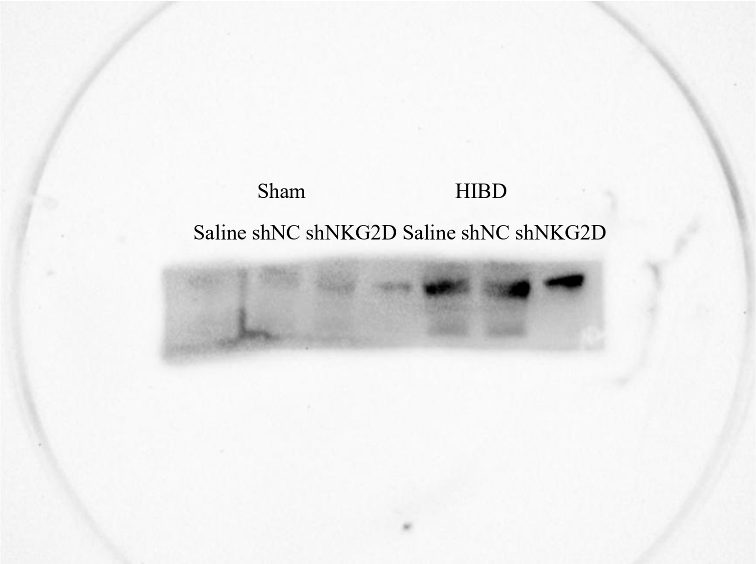

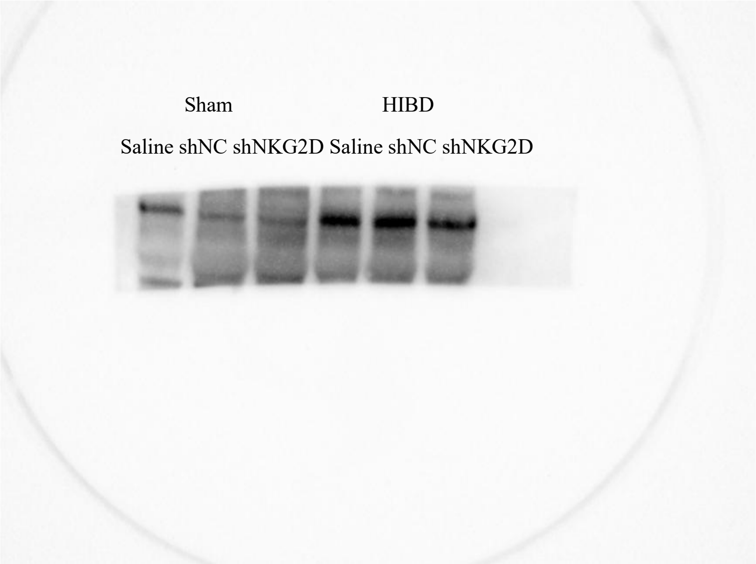

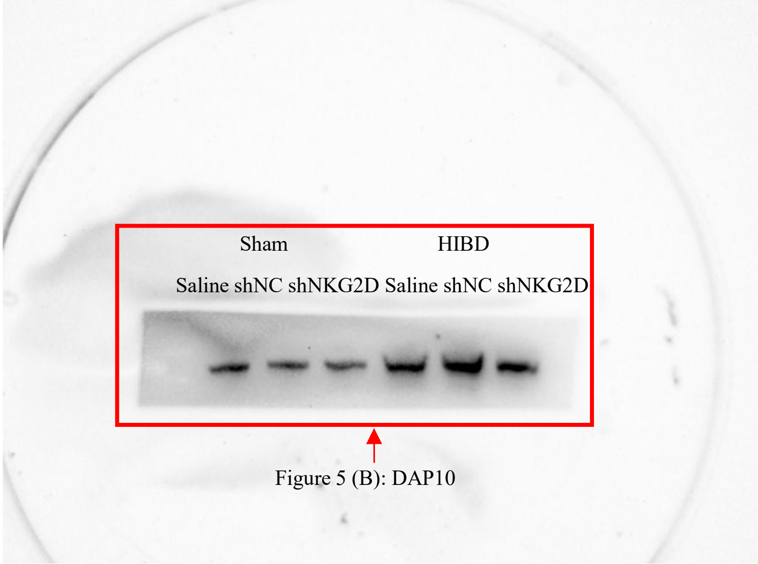

Figure 5 (B): DAP10

β-actin

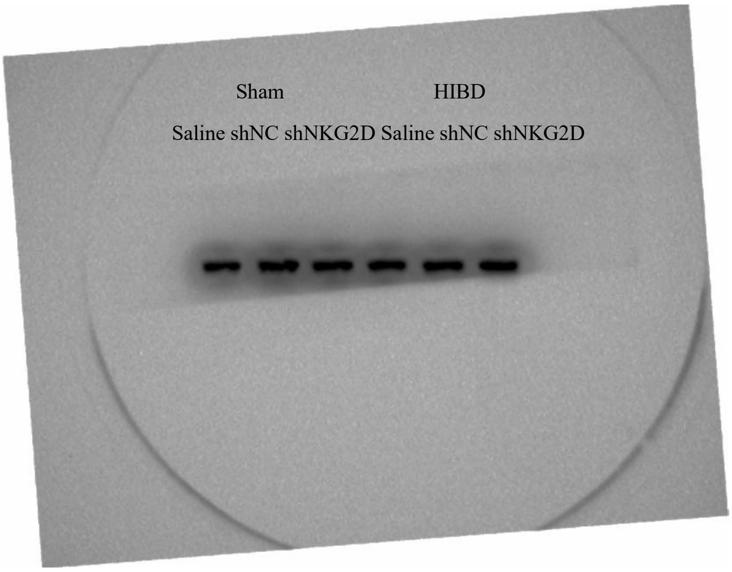
Sham HIBD
Saline shNC shNKG2D Saline shNC shNKG2D

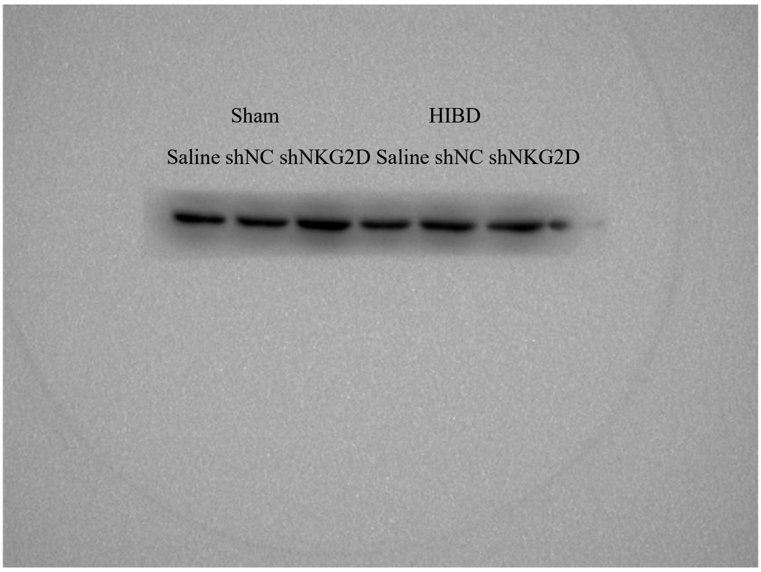
Sham HIBD
Saline shNC shNKG2D Saline shNC shNKG2D

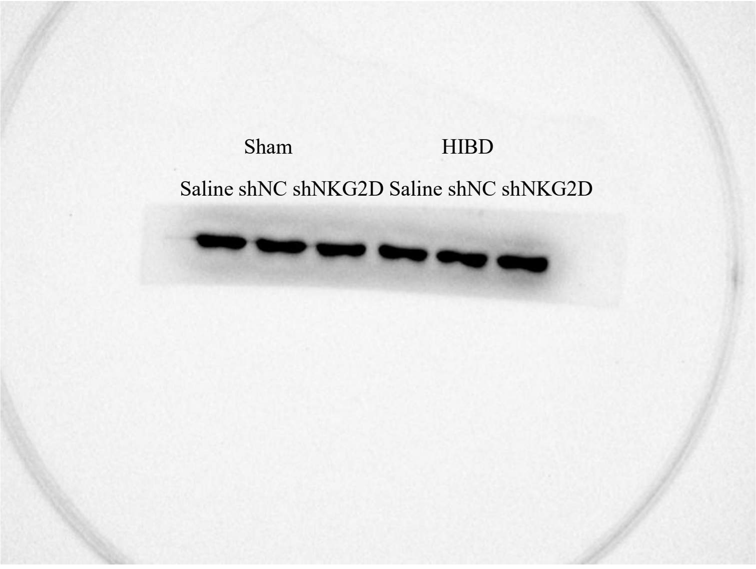
Sham HIBD
Saline shNC shNKG2D Saline shNC shNKG2D

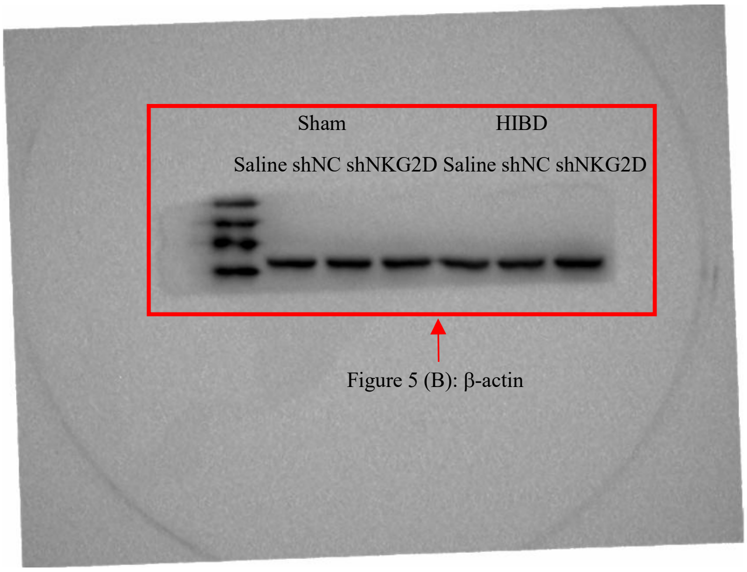

Figure 5 (B): β-actin

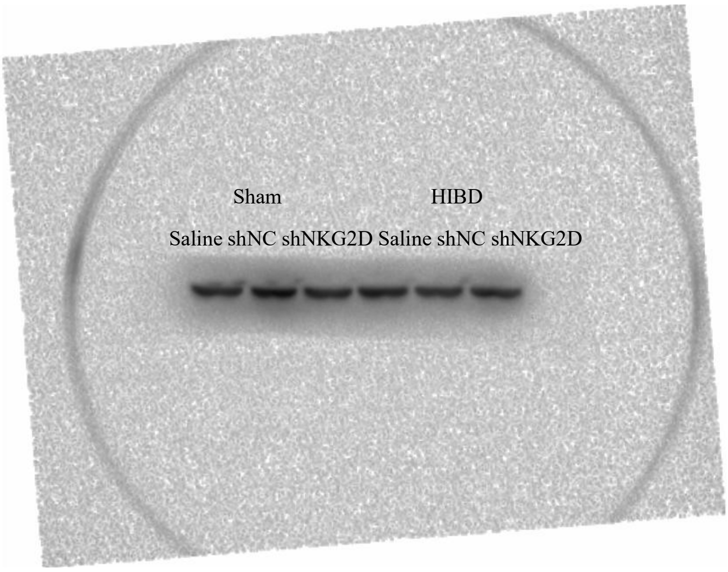

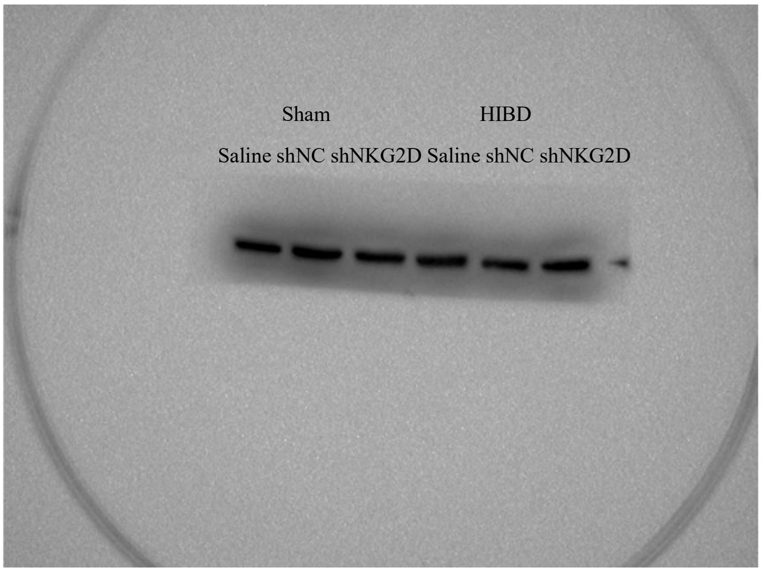

NF-κB p65

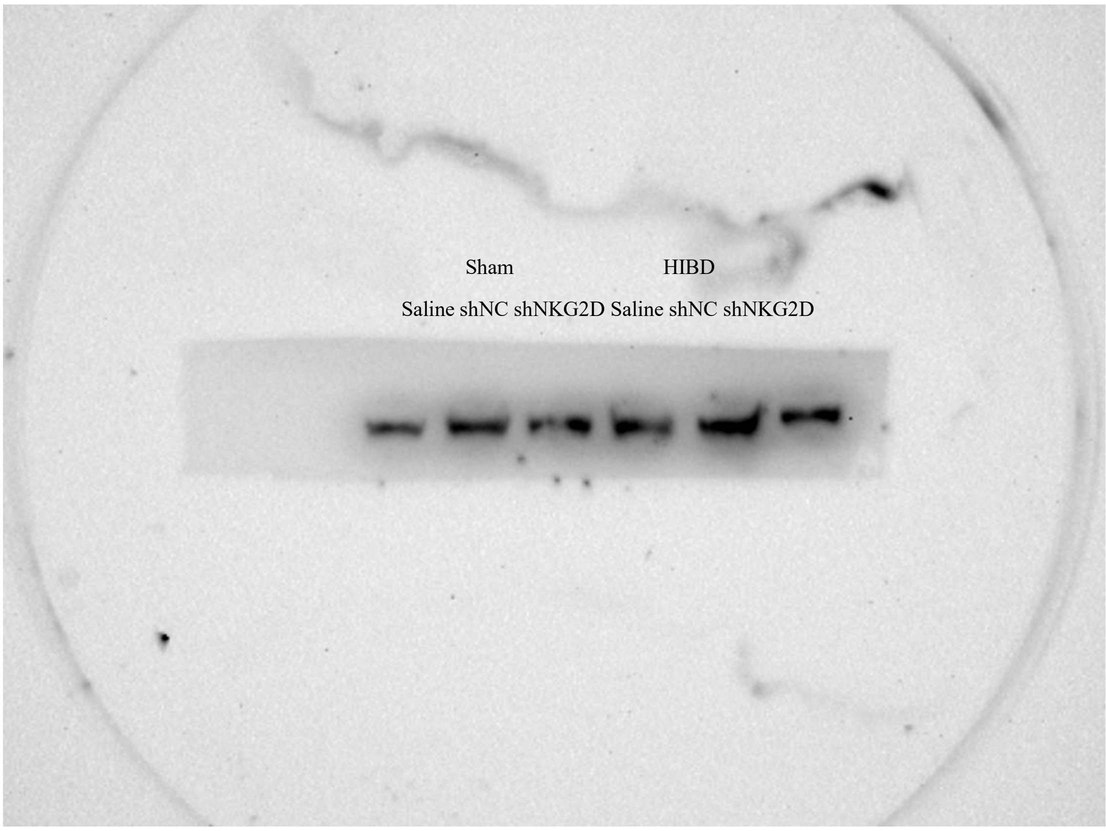

Sham
HIBD
Saline shNC shNKG2D Saline shNC shNKG2D

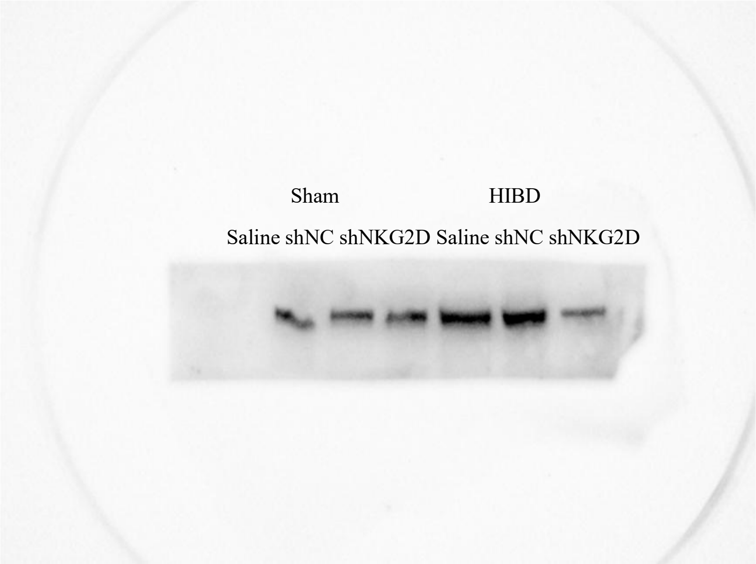

Sham
HIBD
Saline shNC shNKG2D Saline shNC shNKG2D

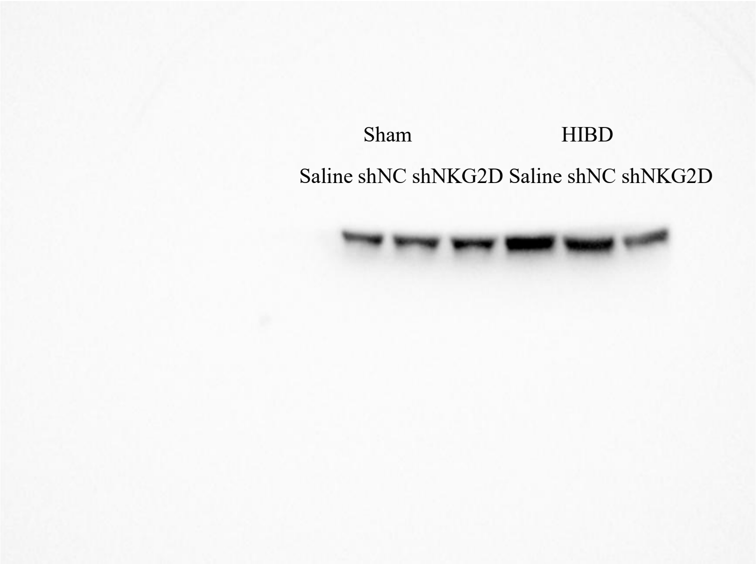

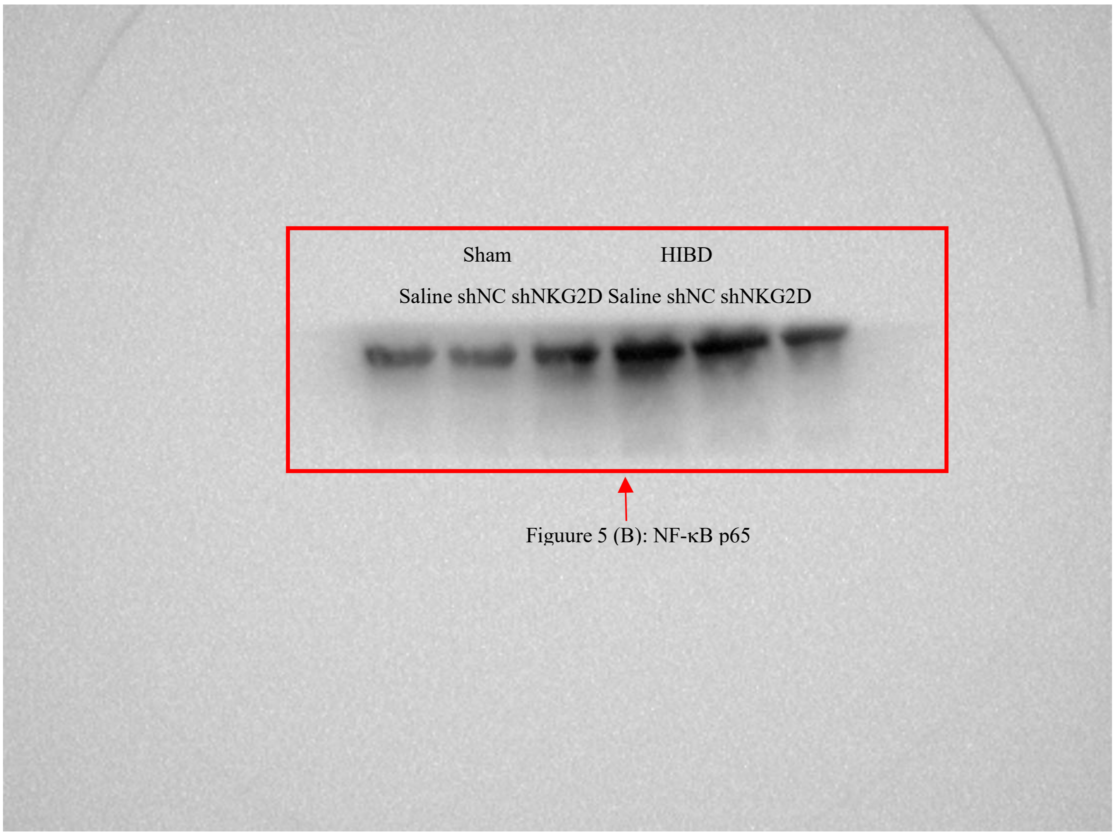

Figuure 5 (B): NF-κB p65

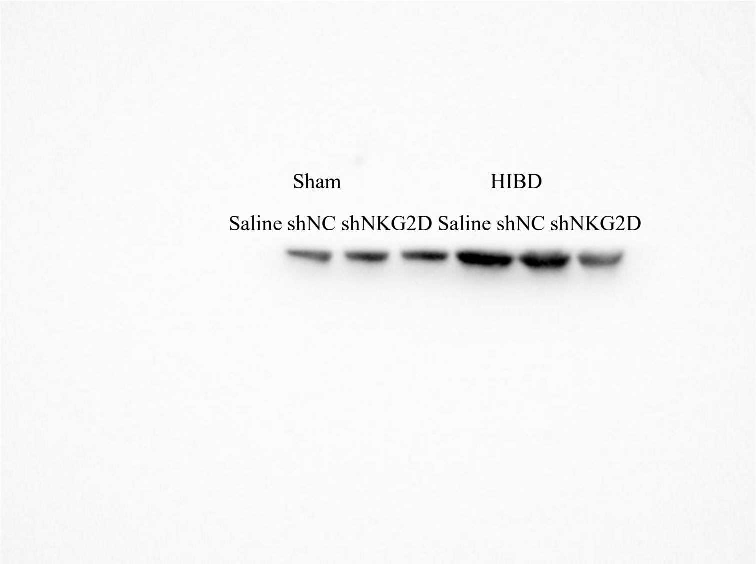
Sham
HIBD
Saline shNC shNKG2D Saline shNC shNKG2D

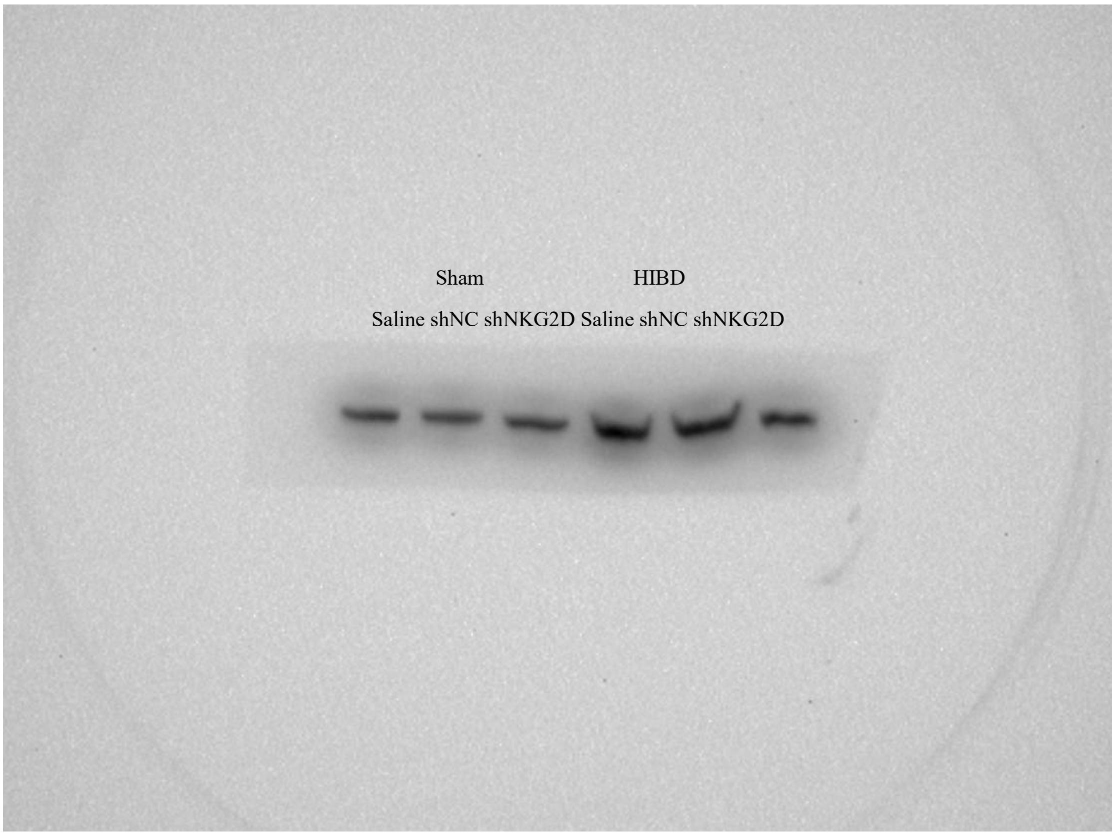
Sham
HIBD
Saline shNC shNKG2D Saline shNC shNKG2D

histone H3

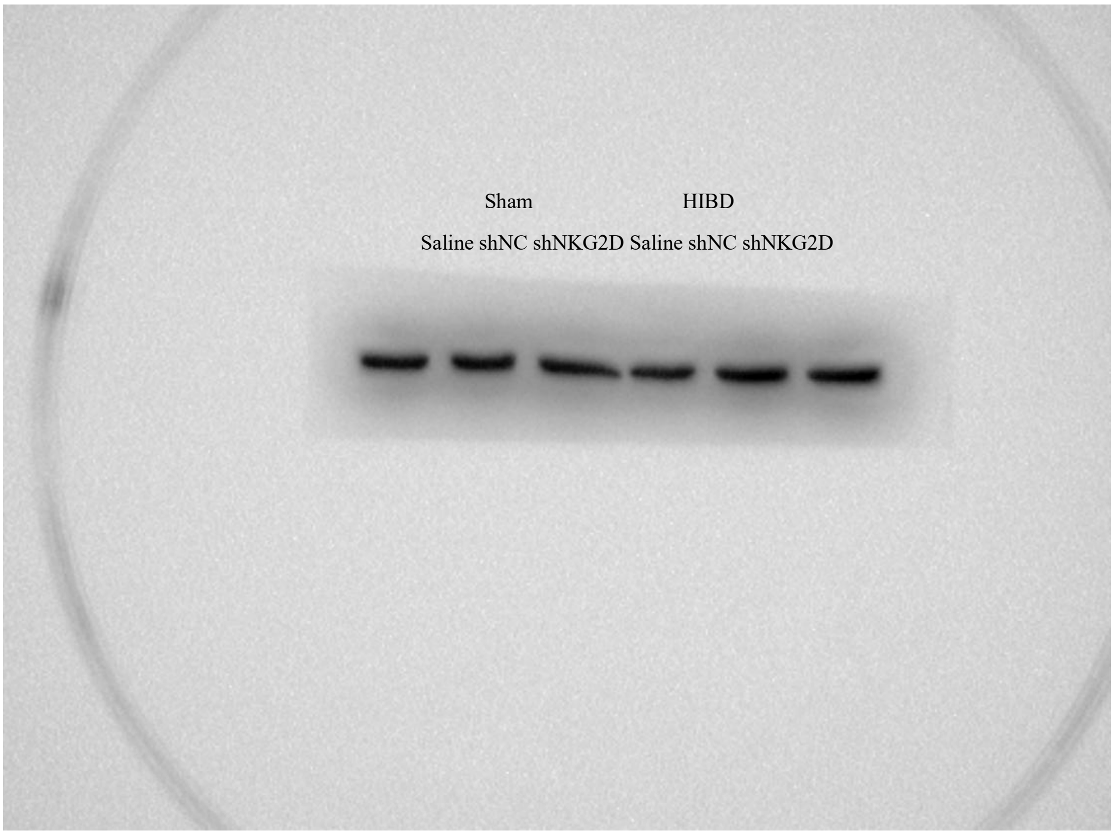
Sham
HIBD
Saline shNC shNKG2D Saline shNC shNKG2D

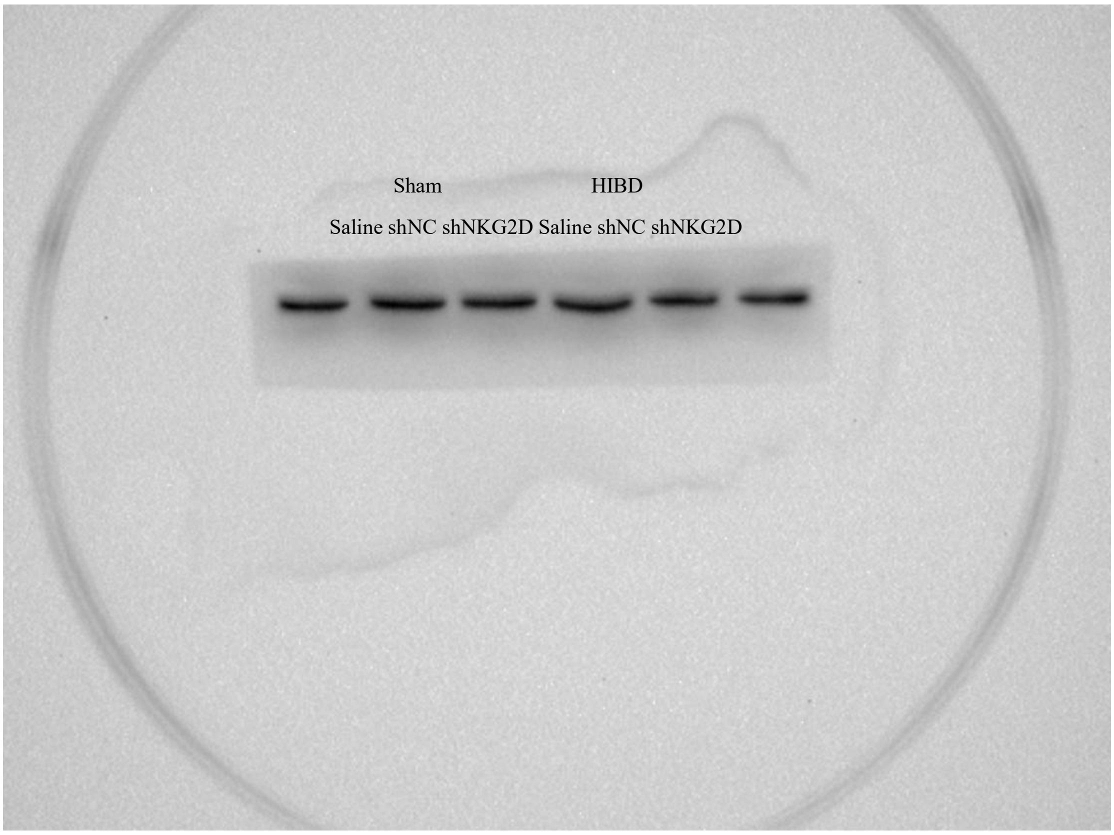
Sham
HIBD
Saline shNC shNKG2D Saline shNC shNKG2D

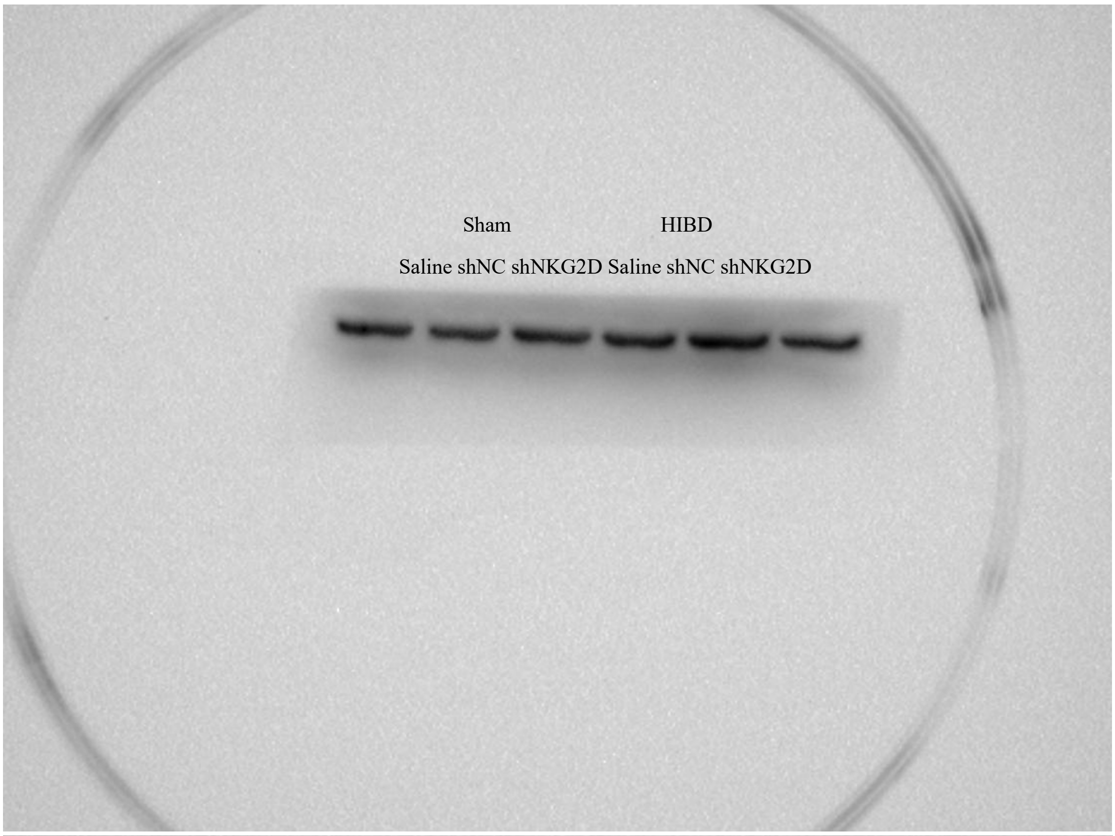
Sham
HIBD
Saline shNC shNKG2D Saline shNC shNKG2D

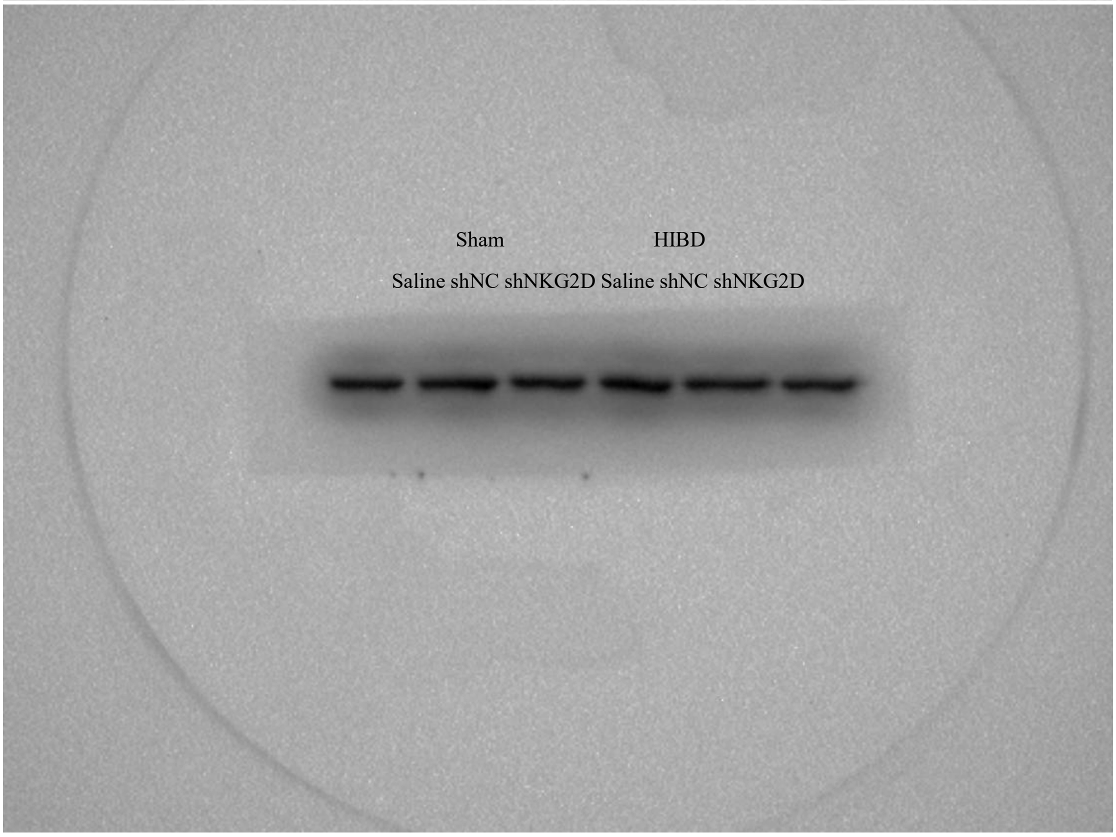
Sham
HIBD
Saline shNC shNKG2D Saline shNC shNKG2D

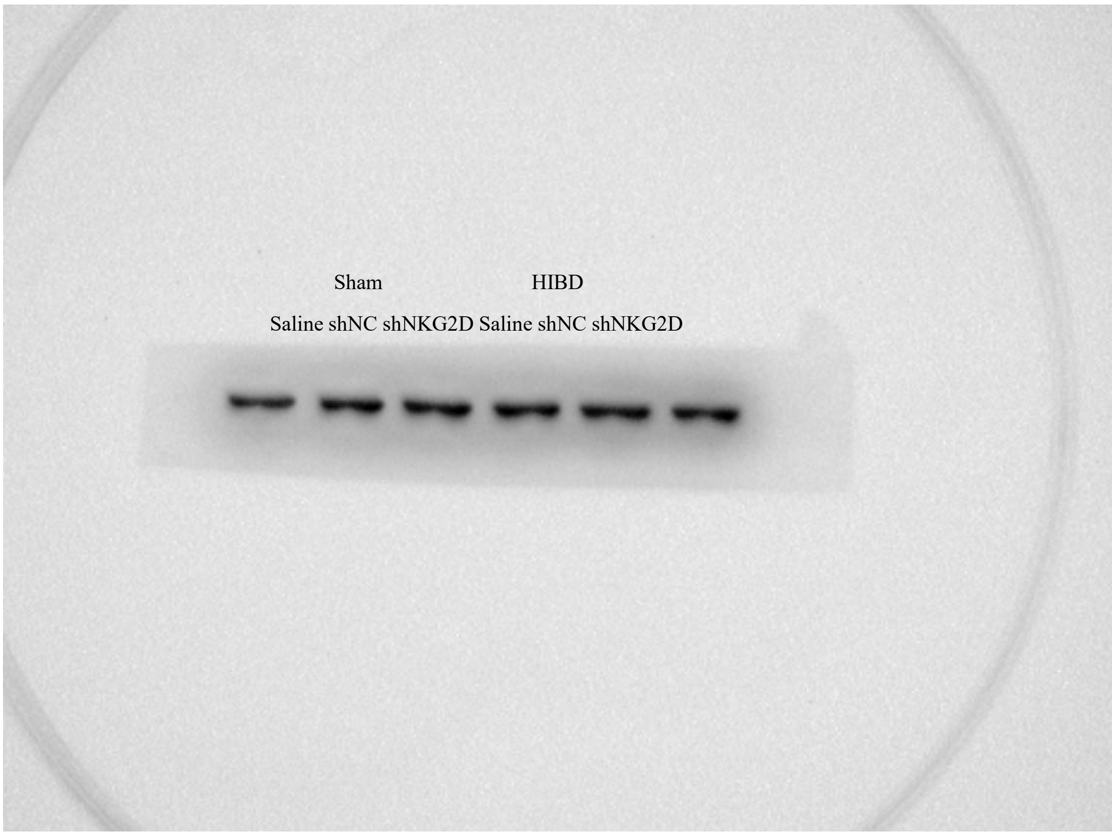

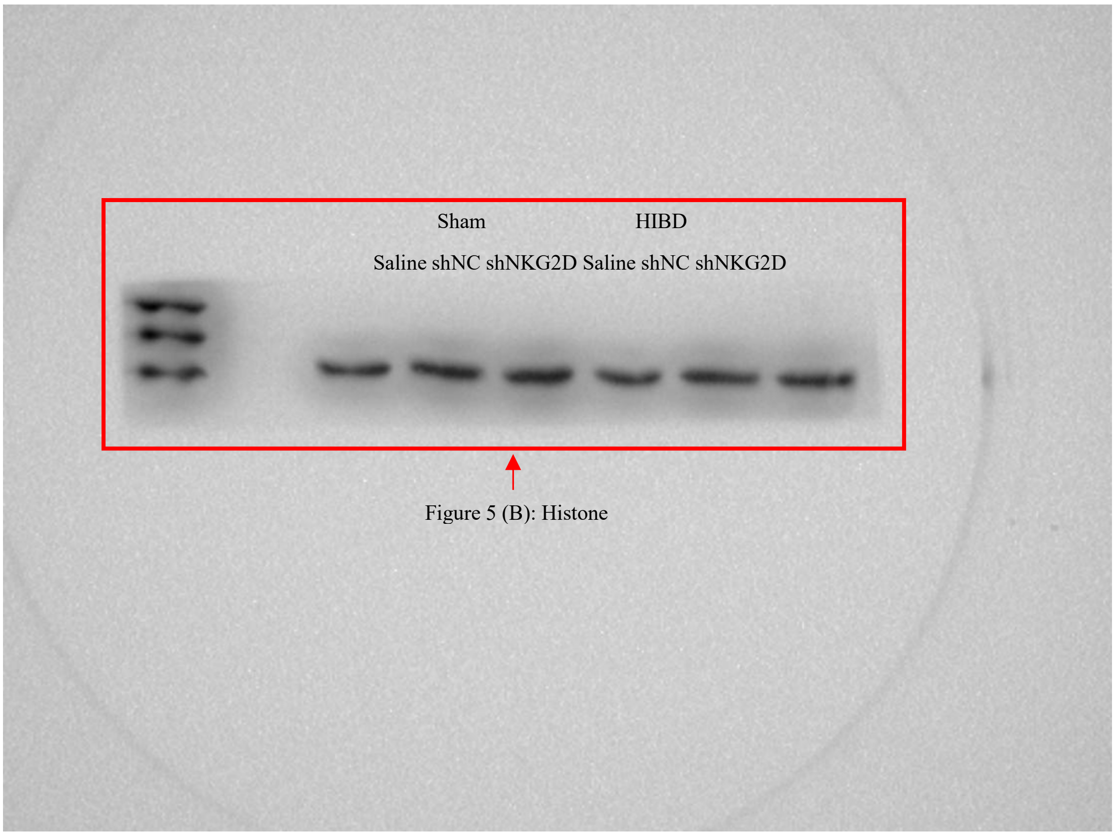

Figure 5 (B): Histone
