# Supplementary material for: NKG2D knockdown improves hypoxic-ischemic brain damage by inhibiting neuroinflammation in neonatal mice
Source: Sci Rep. 2024 Jan 28;14:2326. doi: 10.1038/s41598-024-52780-3 (PMC10822867; doi:10.1038/s41598-024-52780-3)
Supplement: Supplementary file 2 — Supplementary Information. [file 41598_2024_52780_MOESM2_ESM.pdf]

Figure 1. NKG2D was induced by HIBD in newborn mice. (D) Representative immunoblot of NKG2D expression in the brain in neonatal mice.

NKG2D

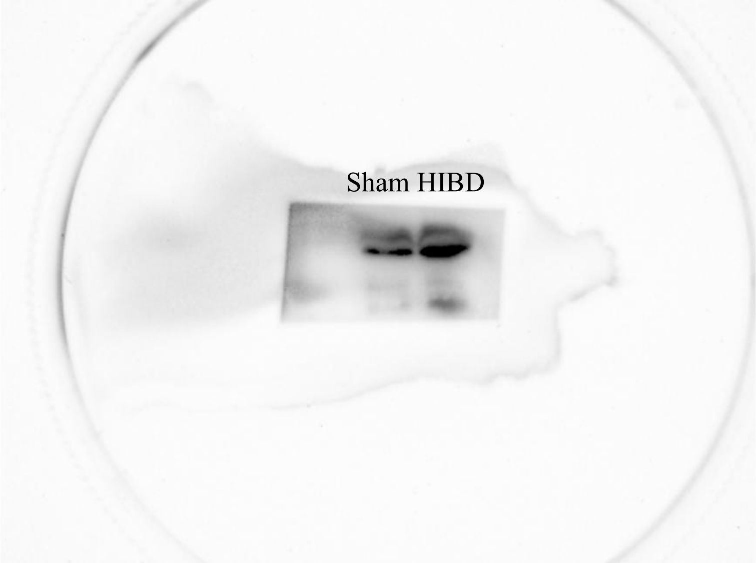

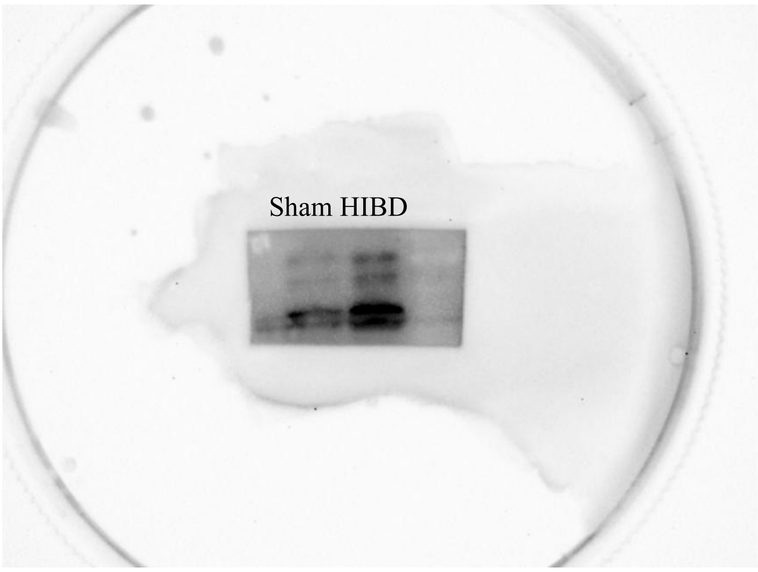

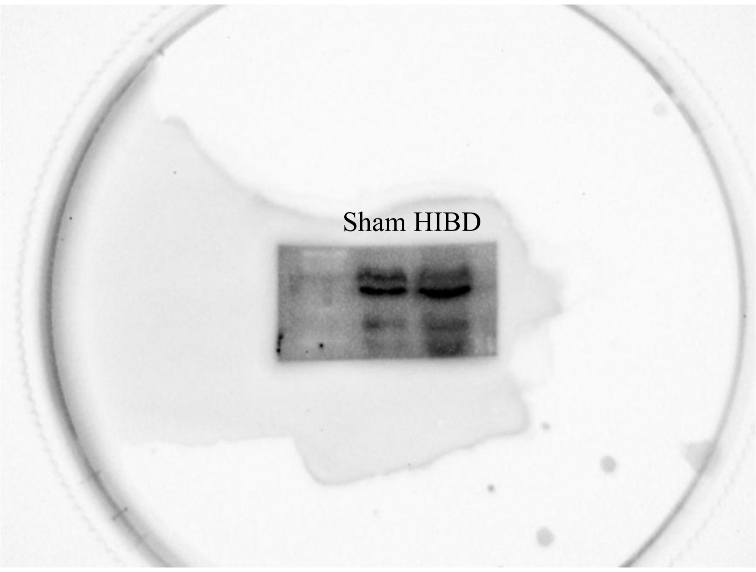
Sham HIBD

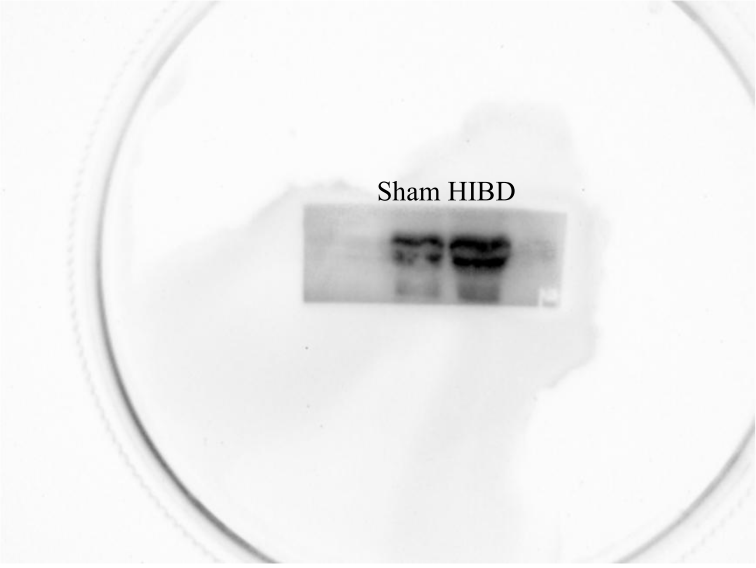
Sham HIBD

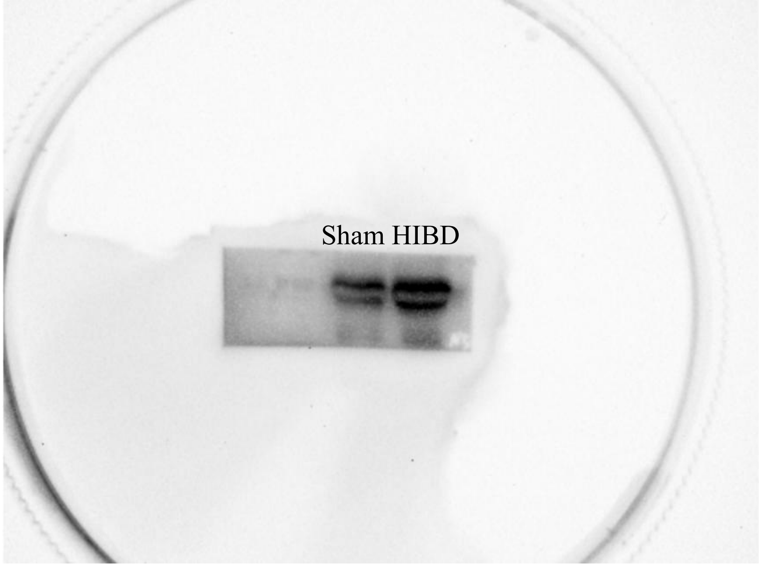
Sham HIBD

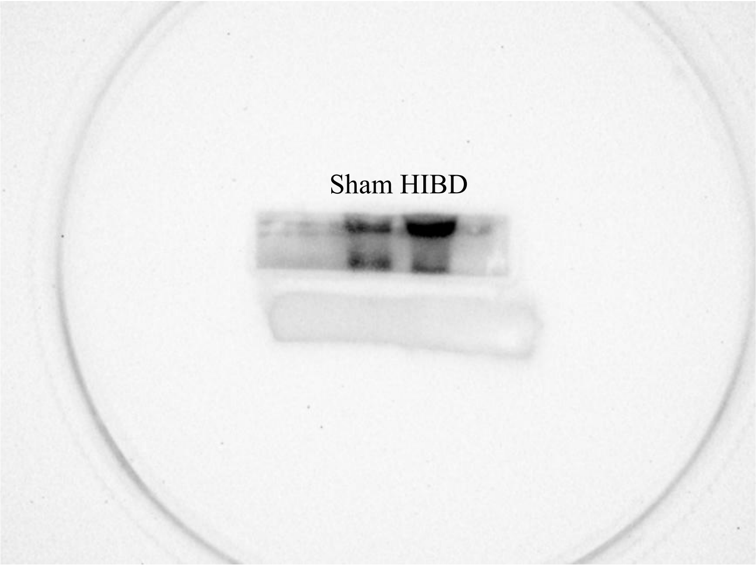

β-actin

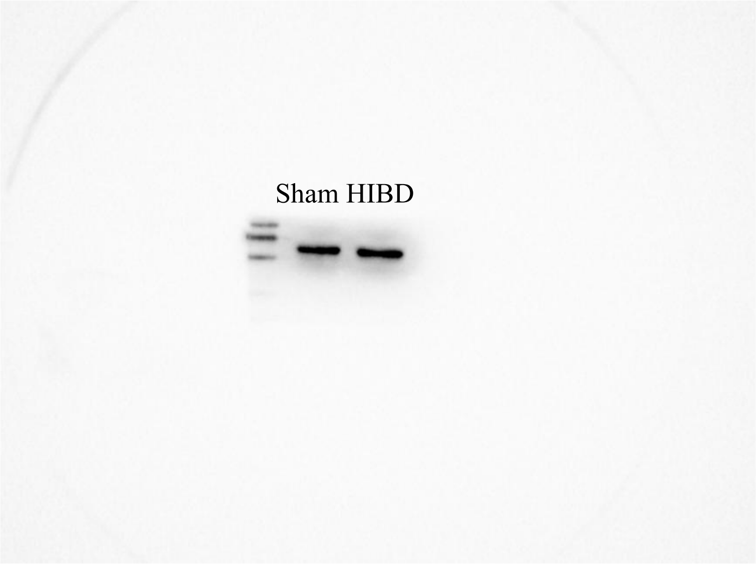

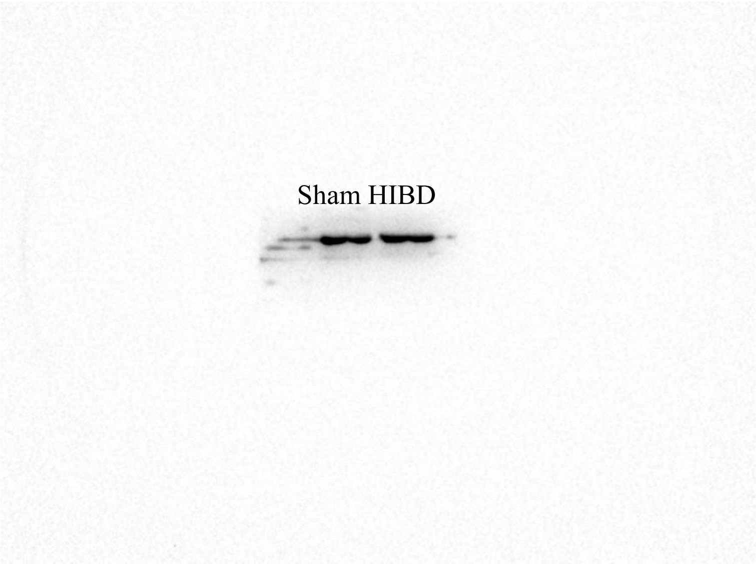

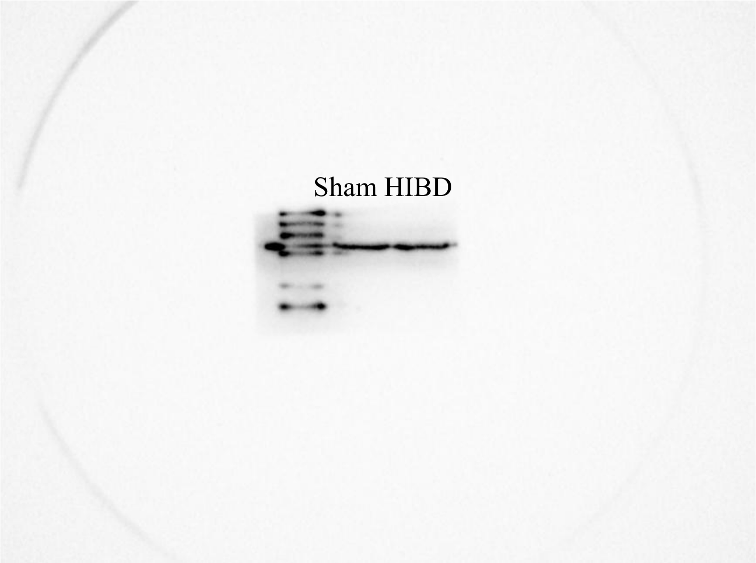
Sham HIBD

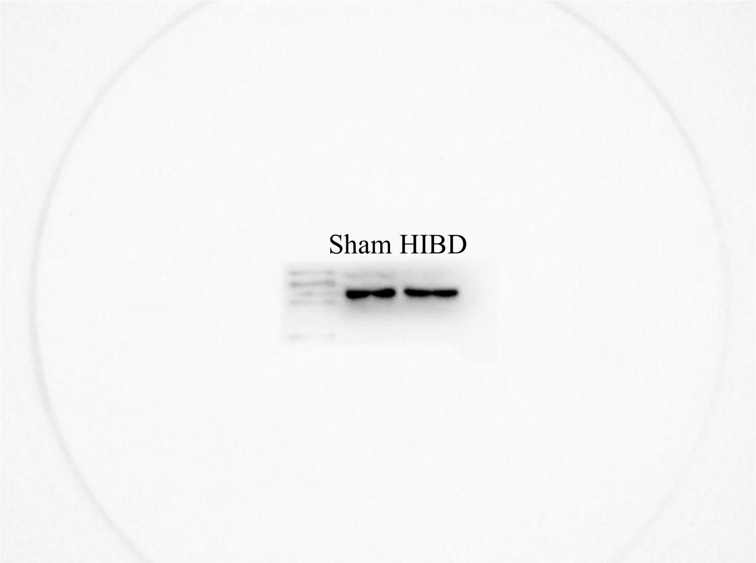
Sham HIBD

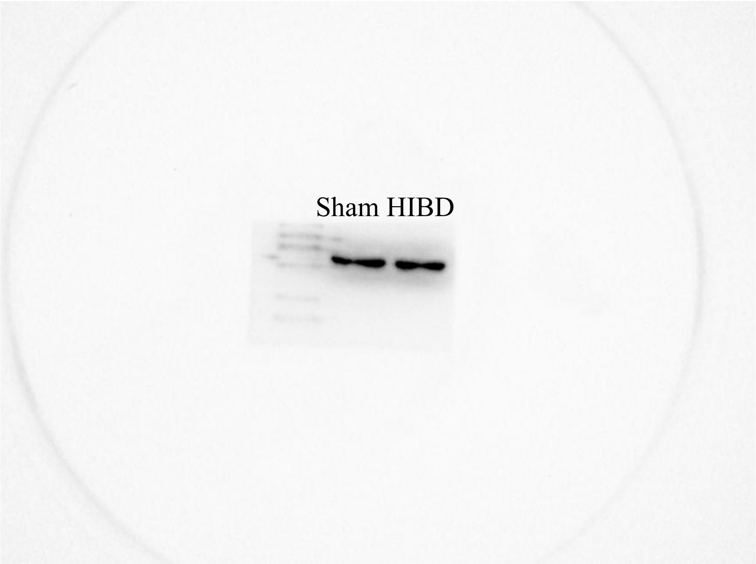
Sham HIBD

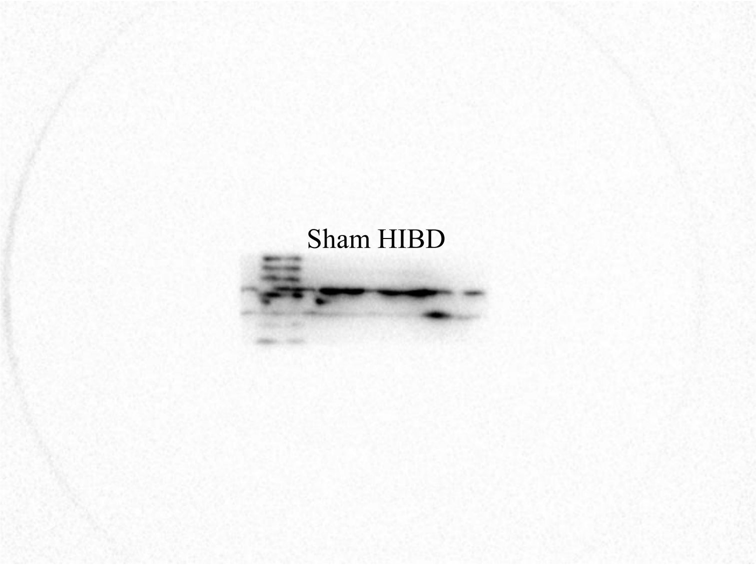
Sham HIBD

Figure 2. shNKG2D decreased the protein expression of NKG2D in the brain in mice. (A) Representative immunoblot of NKG2D in brain in mice.

NKG2D

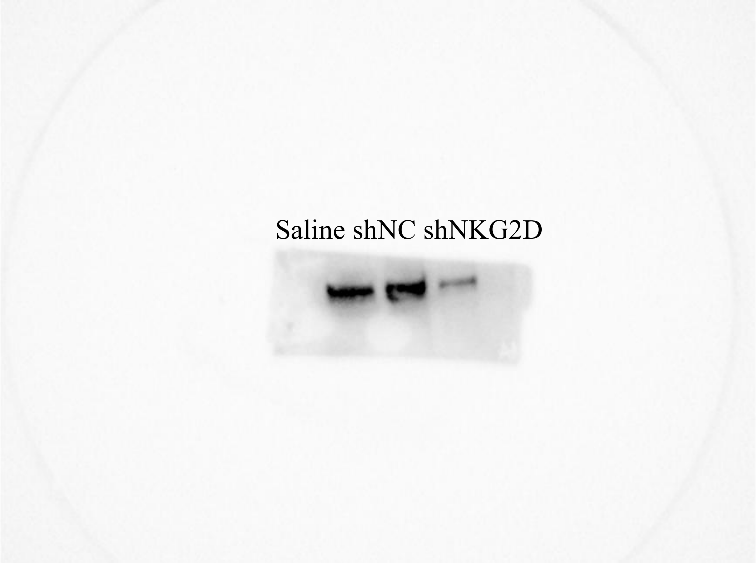

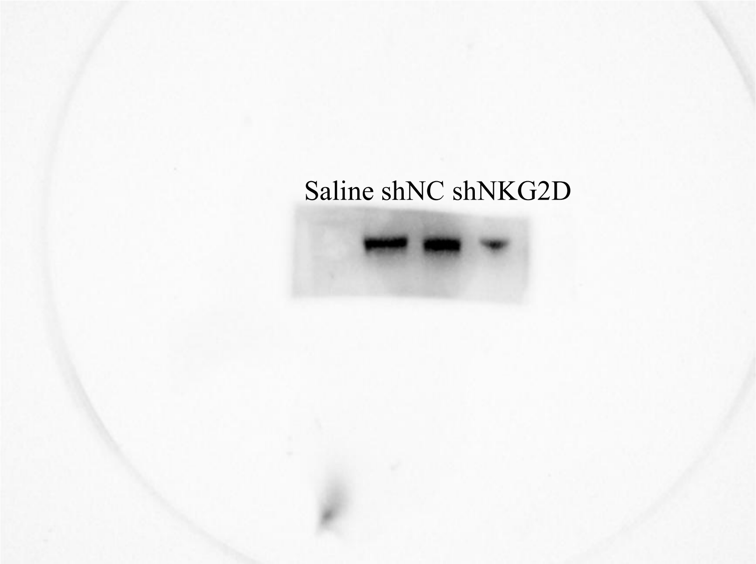

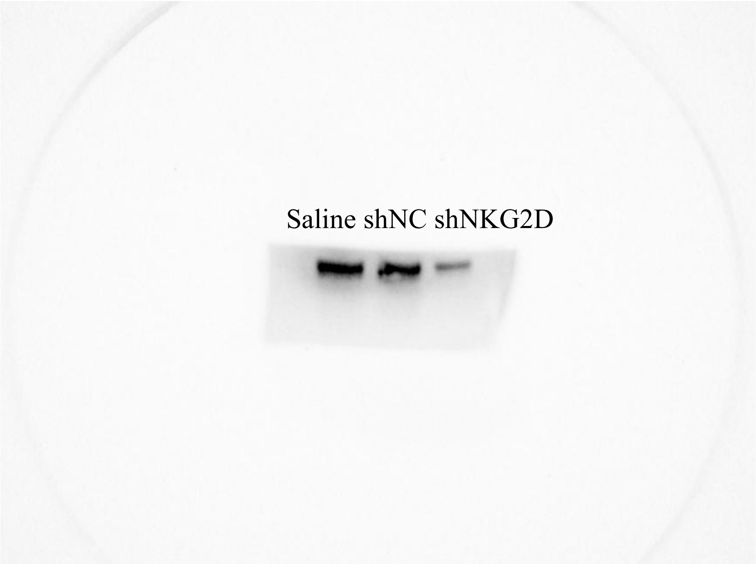
Saline shNC shNKG2D

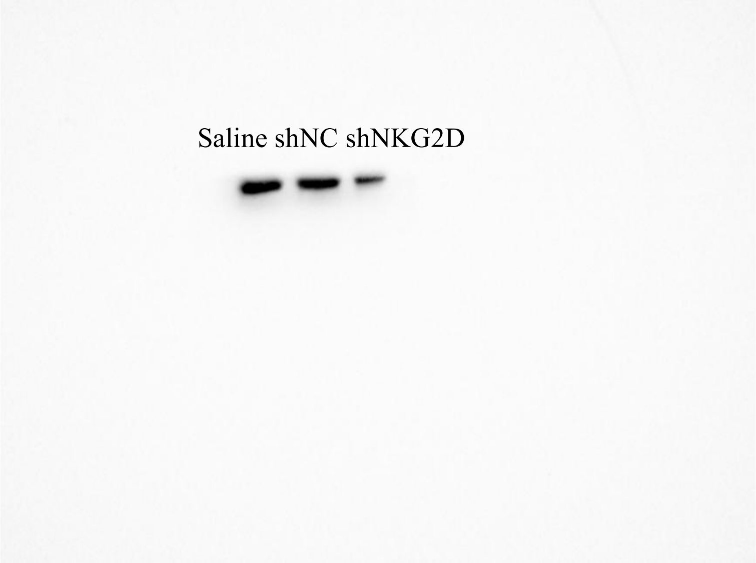
Saline shNC shNKG2D

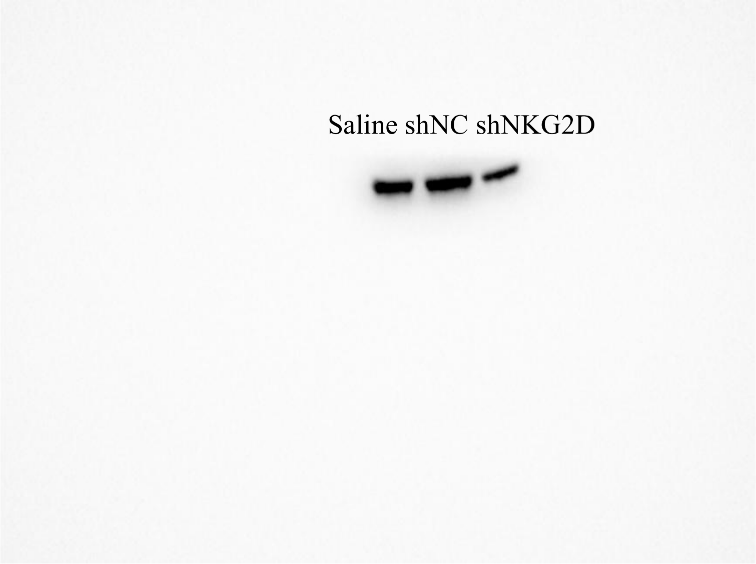
Saline shNC shNKG2D

β-actin

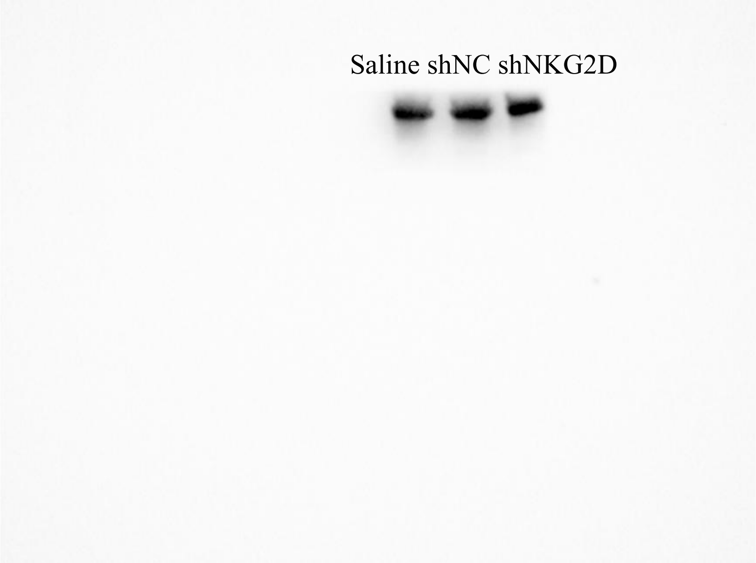
Saline shNC shNKG2D

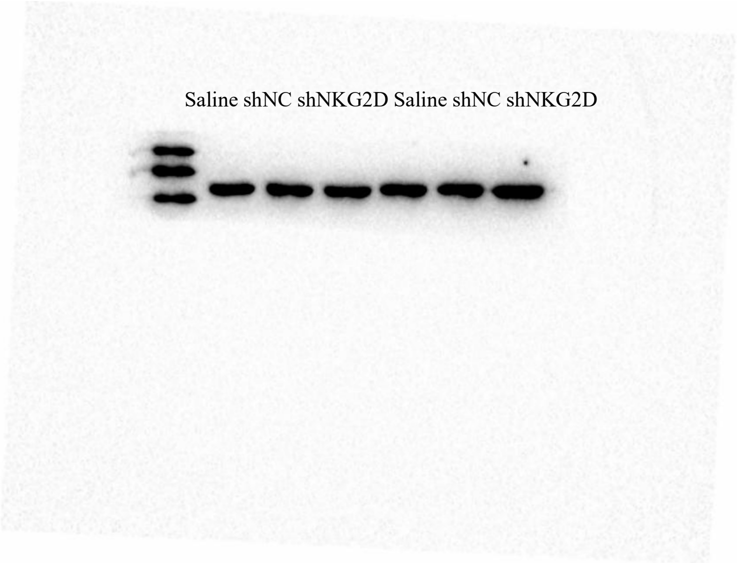
Saline shNC shNKG2D Saline shNC shNKG2D

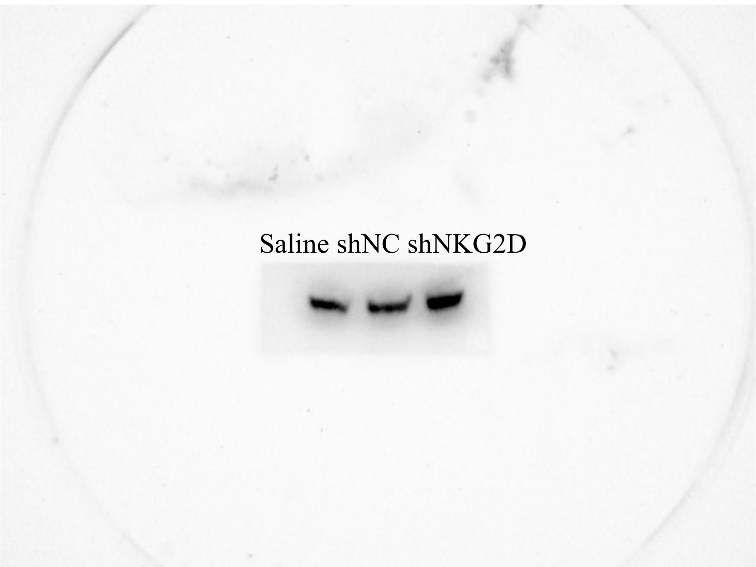
Saline shNC shNKG2D

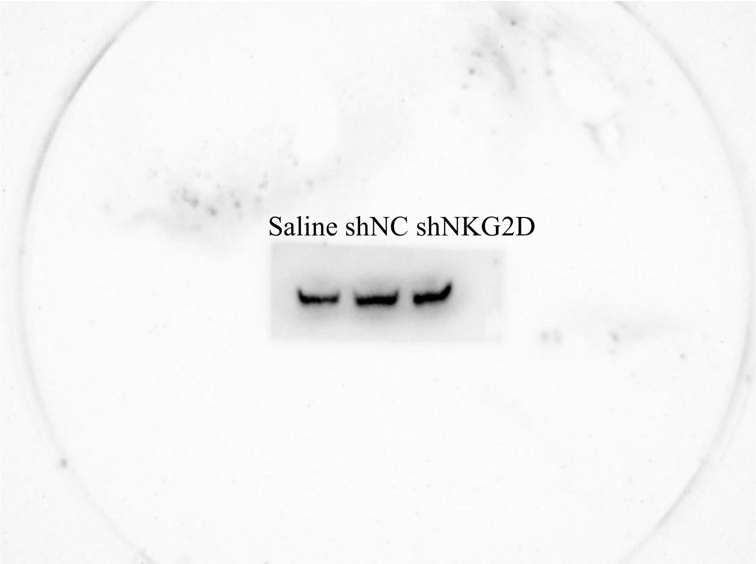
Saline shNC shNKG2D

Figure 5. NKG2D knockdown inhibited the NF-B pathway in the brain in neonatal HIBD mice. (B) Representative immunoblots of NKG2D, H60, Rae-1, and DAP10 expression and translocation of NF-B p65 in neonatal mice.

NKG2D

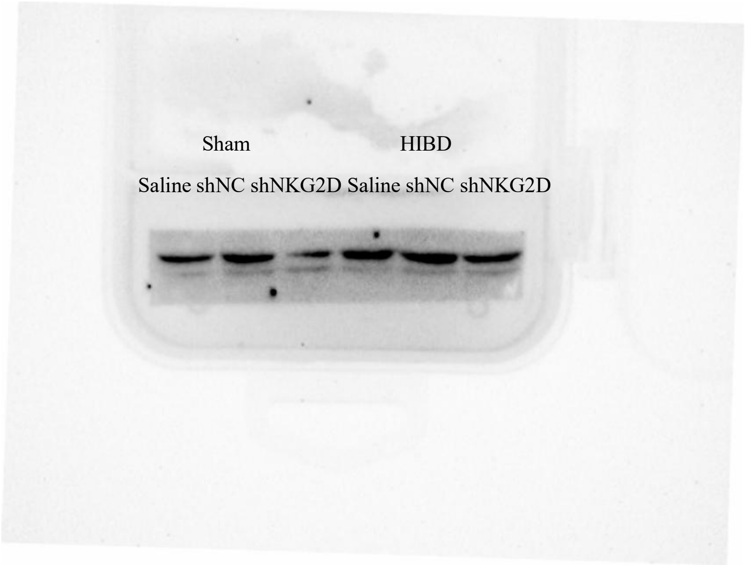

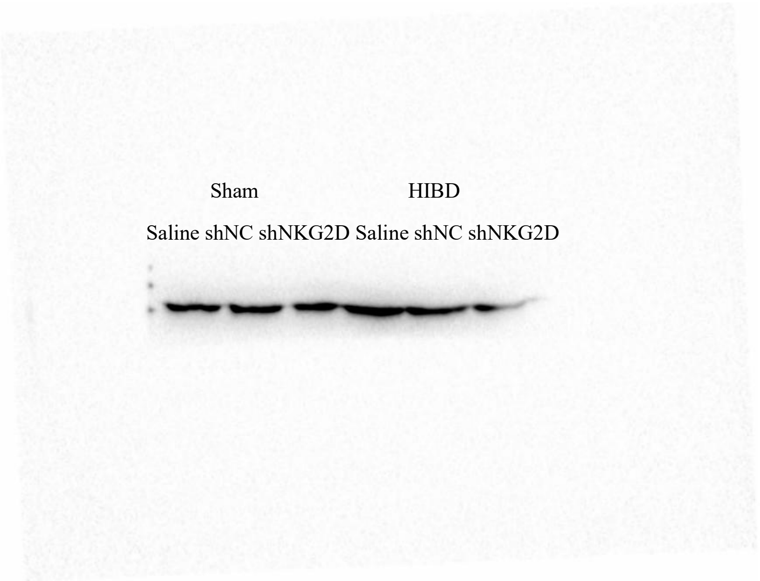

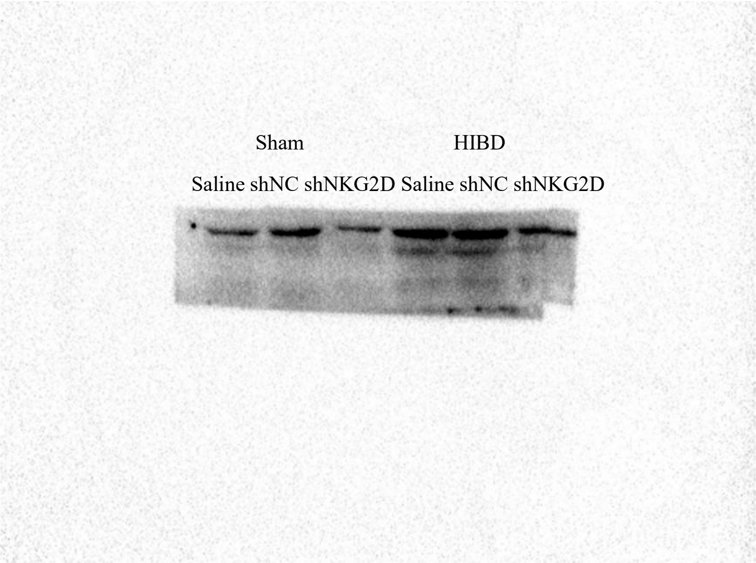
Sham
HIBD
Saline shNC shNKG2D Saline shNC shNKG2D

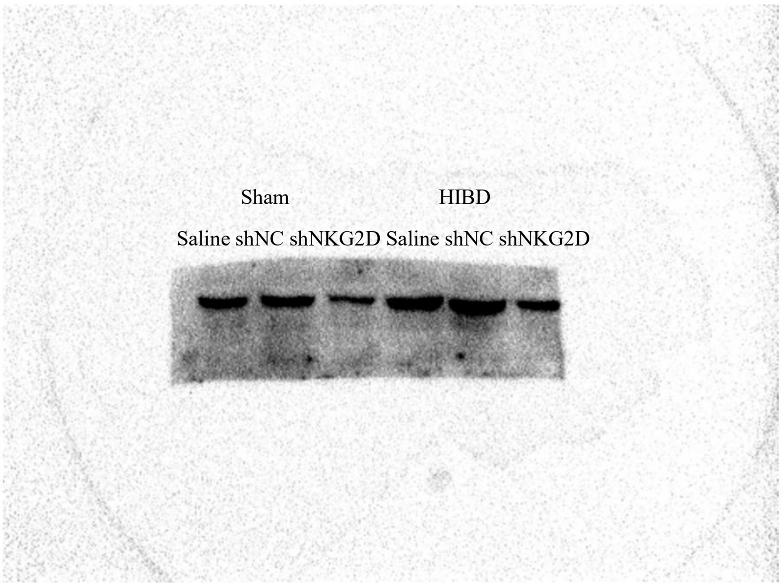
Sham
HIBD
Saline shNC shNKG2D Saline shNC shNKG2D

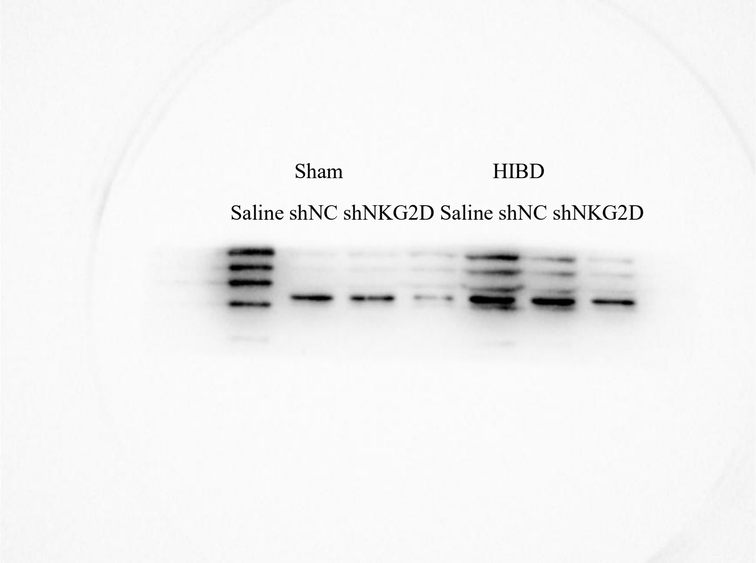
Sham
HIBD
Saline shNC shNKG2D Saline shNC shNKG2D

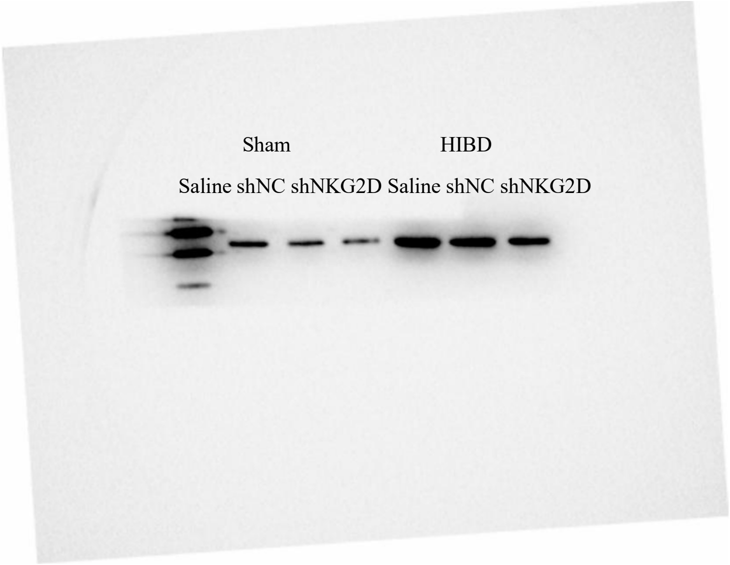
Sham
HIBD
Saline shNC shNKG2D Saline shNC shNKG2D

H60

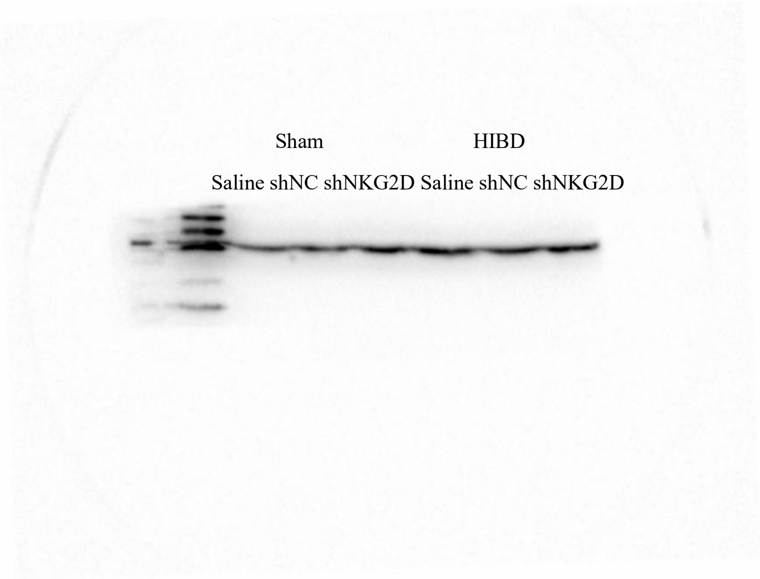

Sham
HIBD
Saline shNC shNKG2D Saline shNC shNKG2D

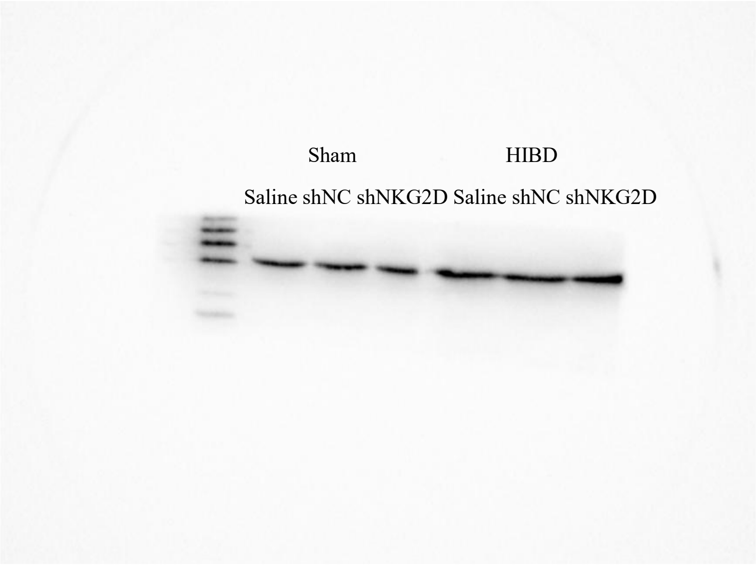

Sham
HIBD
Saline shNC shNKG2D Saline shNC shNKG2D

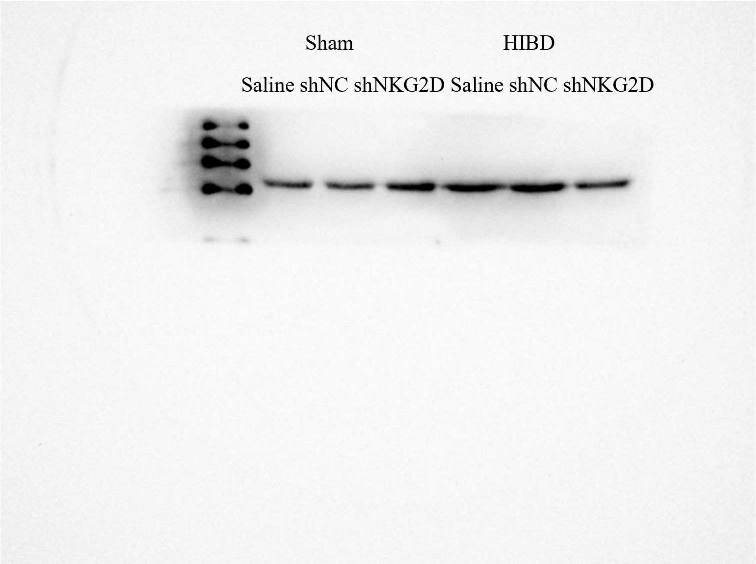

Sham
HIBD
Saline shNC shNKG2D Saline shNC shNKG2D

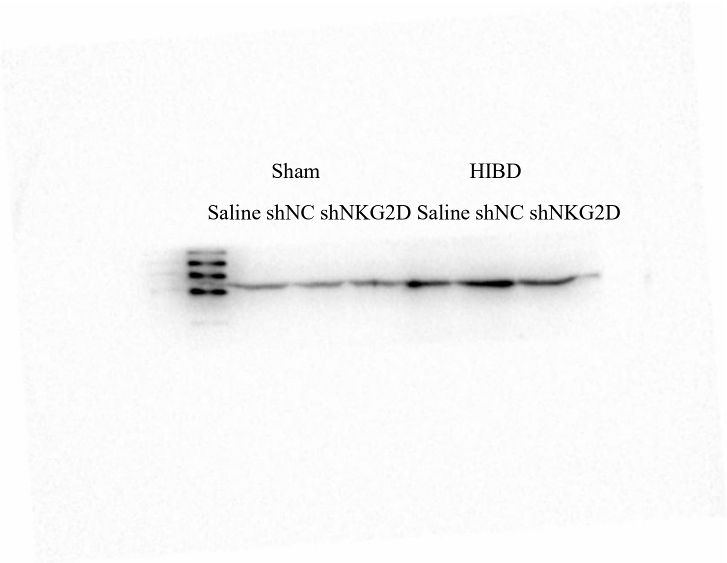
Sham
HIBD
Saline shNC shNKG2D Saline shNC shNKG2D

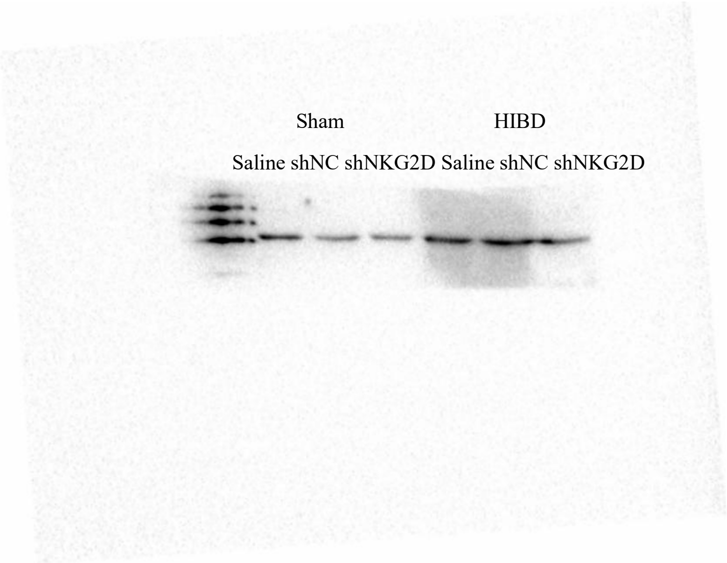
Sham
HIBD
Saline shNC shNKG2D Saline shNC shNKG2D

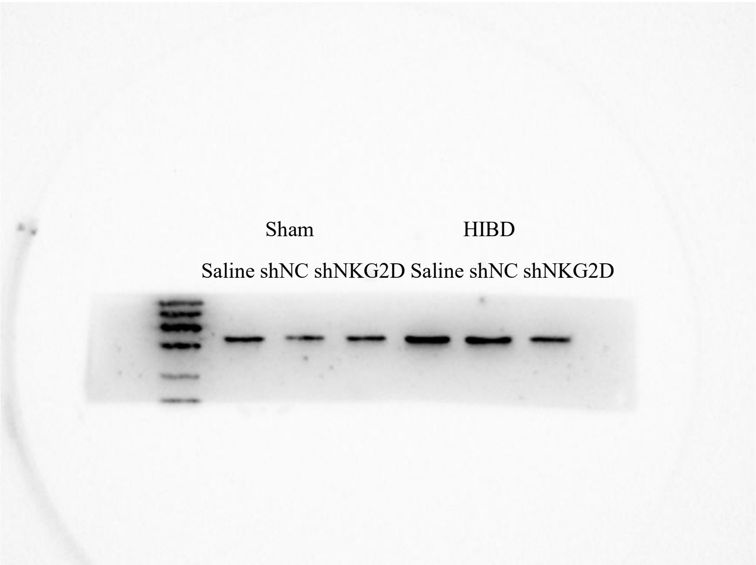
Sham
HIBD
Saline shNC shNKG2D Saline shNC shNKG2D

Rae-1

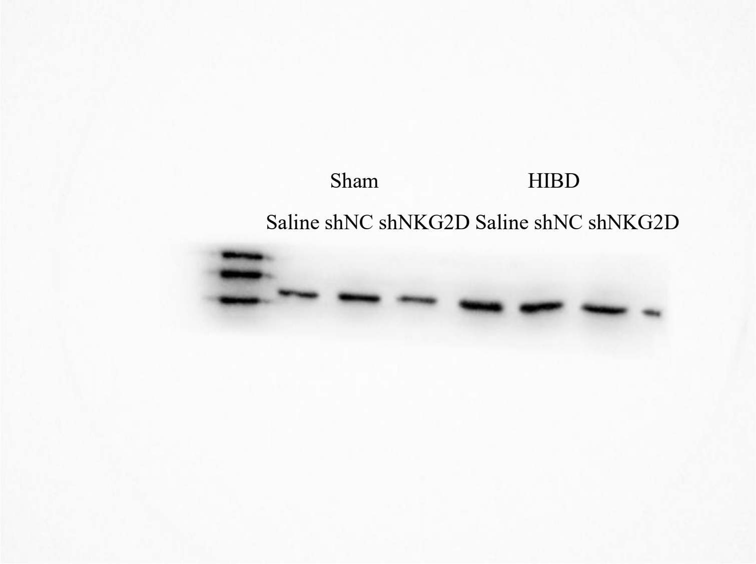
Sham
HIBD
Saline shNC shNKG2D Saline shNC shNKG2D

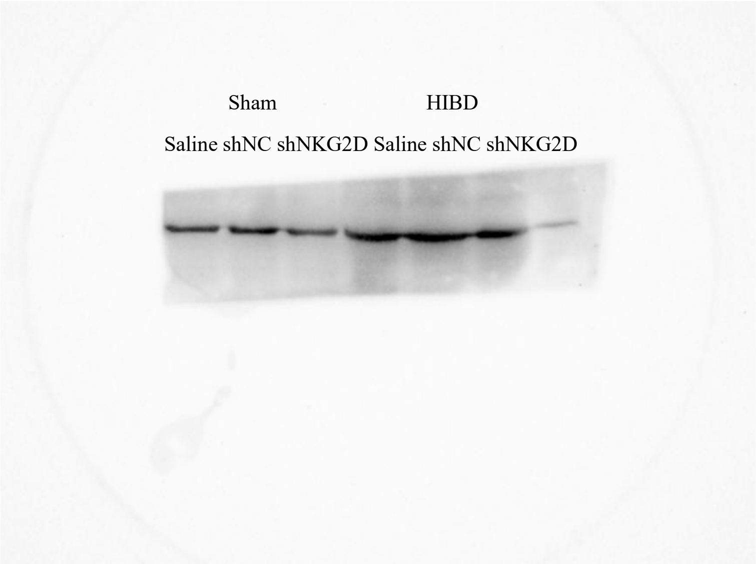
Sham
HIBD
Saline shNC shNKG2D Saline shNC shNKG2D

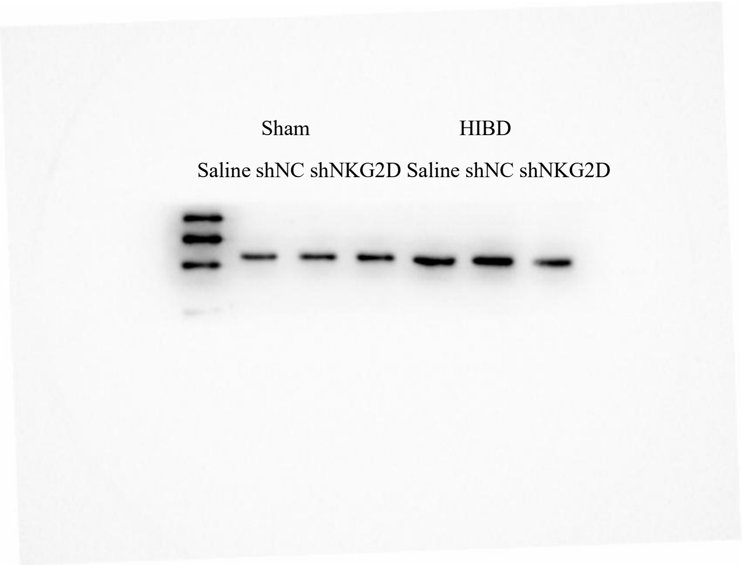
Sham
HIBD
Saline shNC shNKG2D Saline shNC shNKG2D

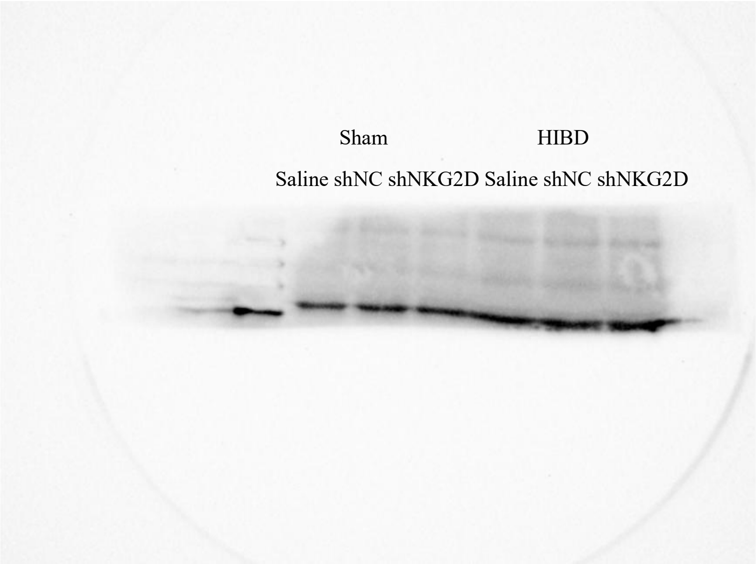
Sham
HIBD
Saline shNC shNKG2D Saline shNC shNKG2D

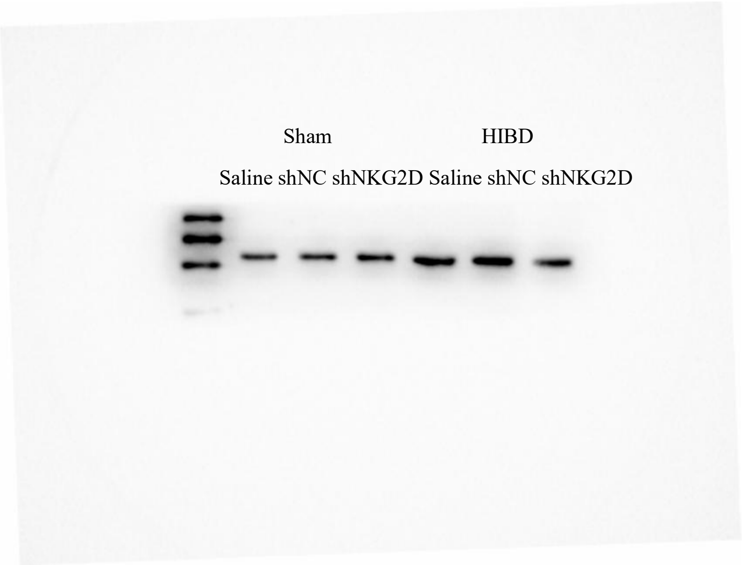
Sham
HIBD
Saline shNC shNKG2D Saline shNC shNKG2D

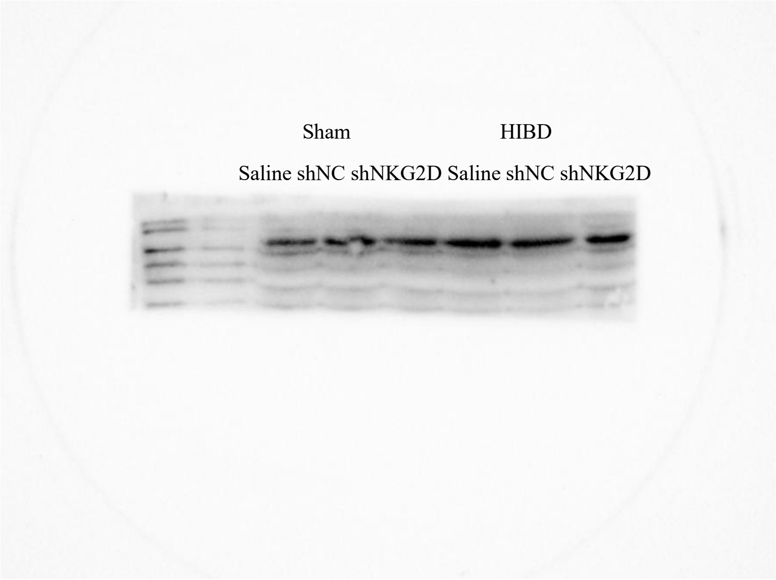
Sham
HIBD
Saline shNC shNKG2D Saline shNC shNKG2D

DAP10

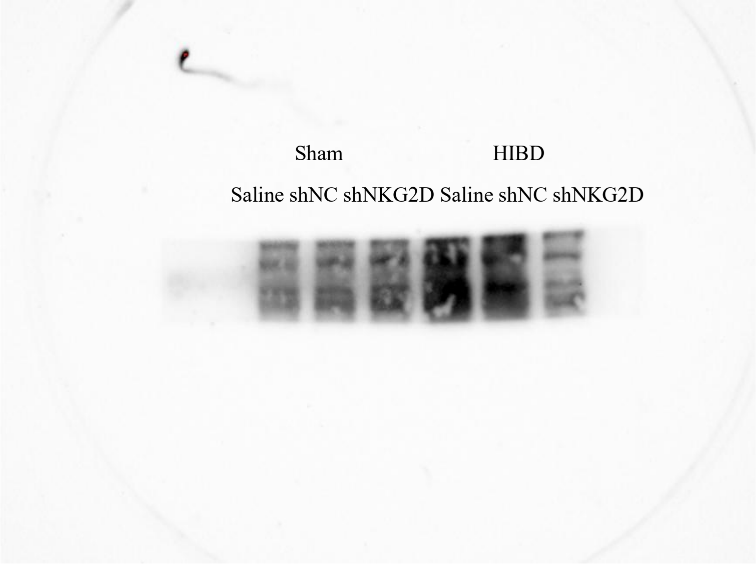
Sham
HIBD
Saline shNC shNKG2D Saline shNC shNKG2D

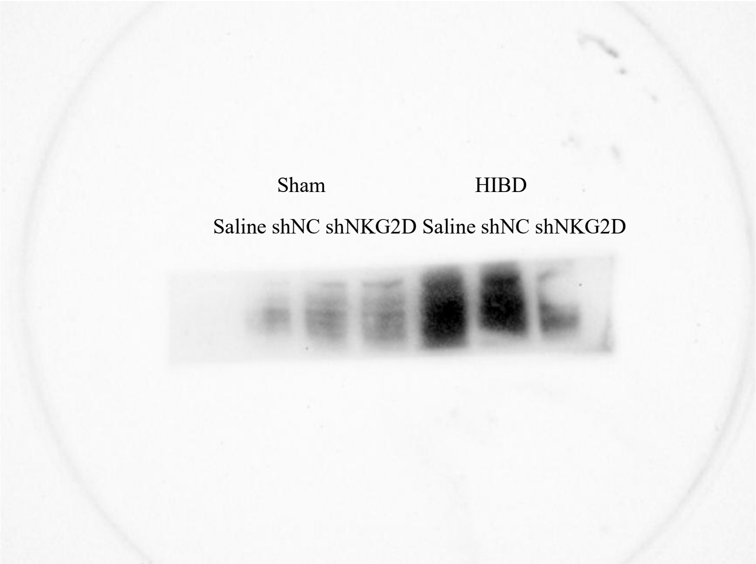
Sham
HIBD
Saline shNC shNKG2D Saline shNC shNKG2D

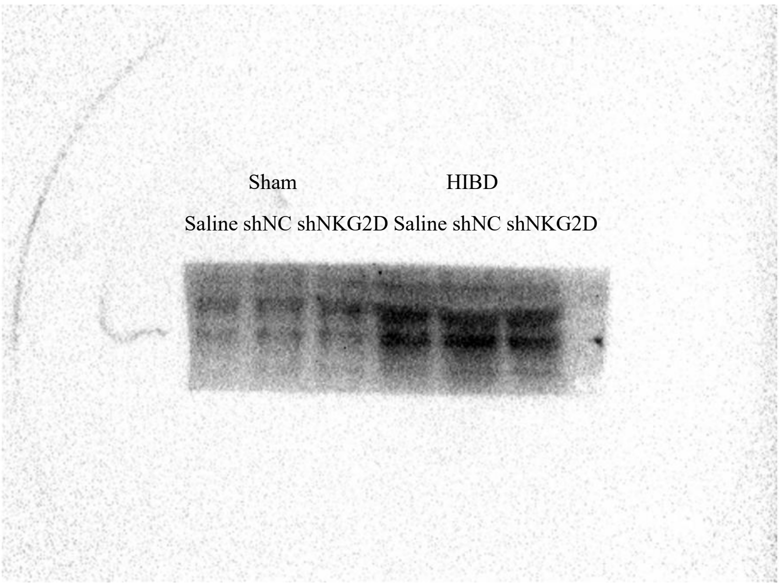
Sham
HIBD
Saline shNC shNKG2D Saline shNC shNKG2D

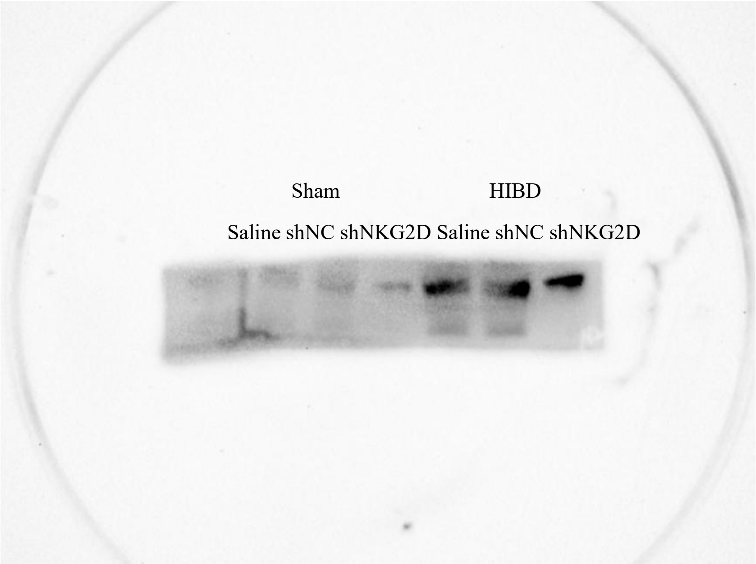
Sham
HIBD
Saline shNC shNKG2D Saline shNC shNKG2D

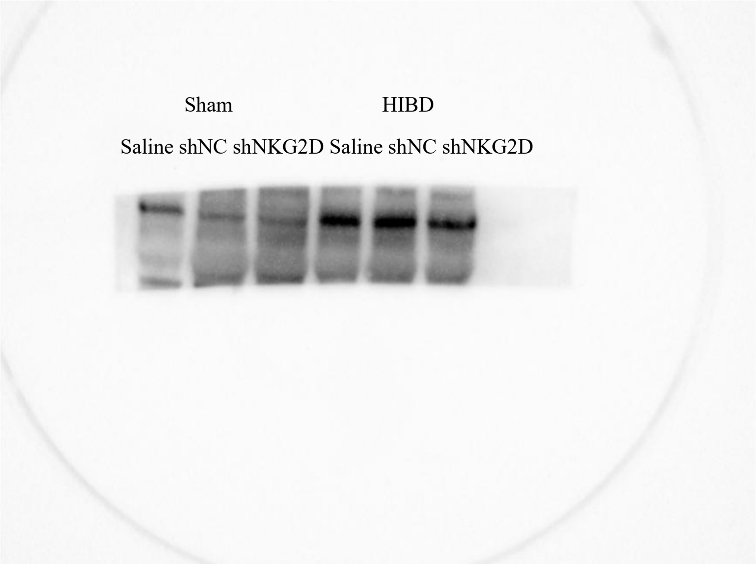
Sham
HIBD
Saline shNC shNKG2D Saline shNC shNKG2D

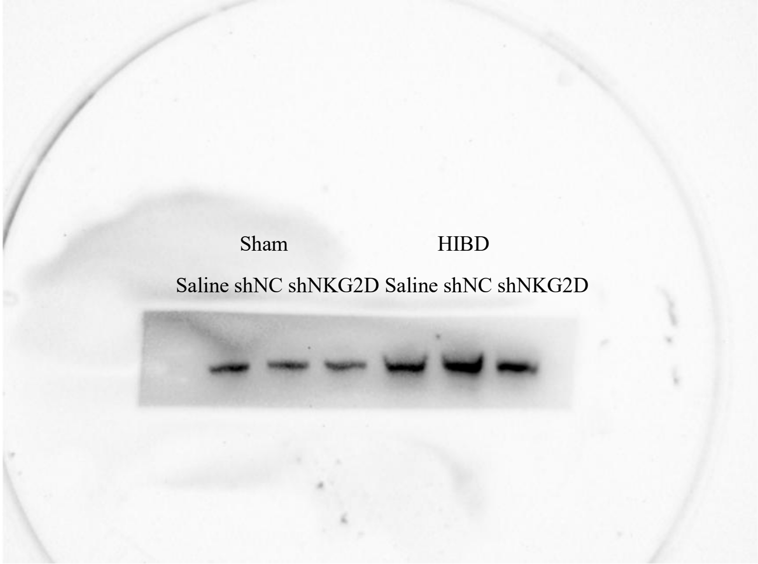
Sham
HIBD
Saline shNC shNKG2D Saline shNC shNKG2D

β-actin

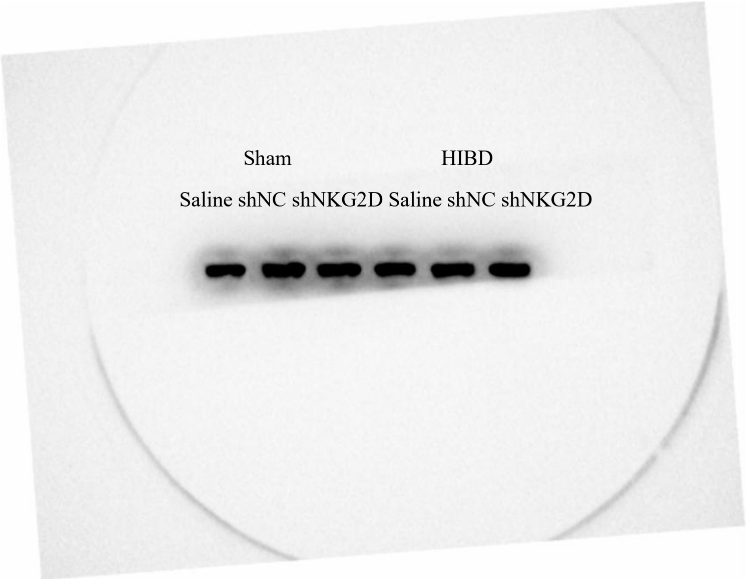
Sham HIBD
Saline shNC shNKG2D Saline shNC shNKG2D

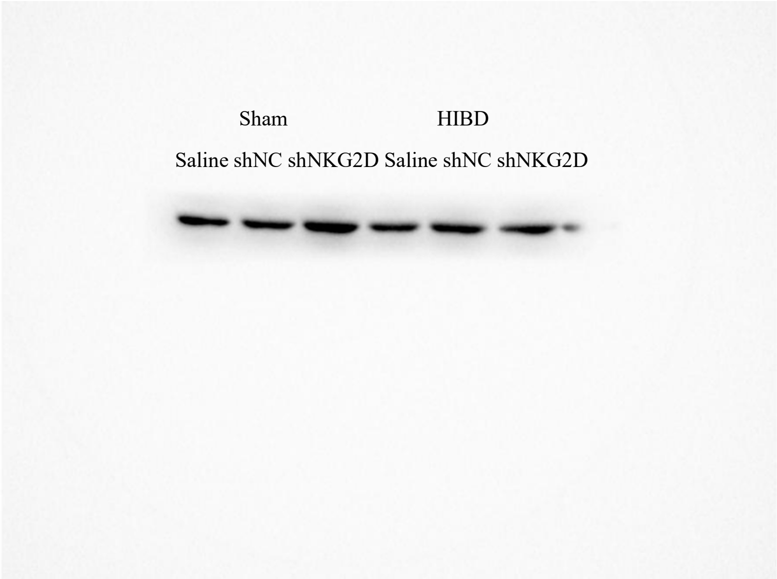
Sham HIBD
Saline shNC shNKG2D Saline shNC shNKG2D

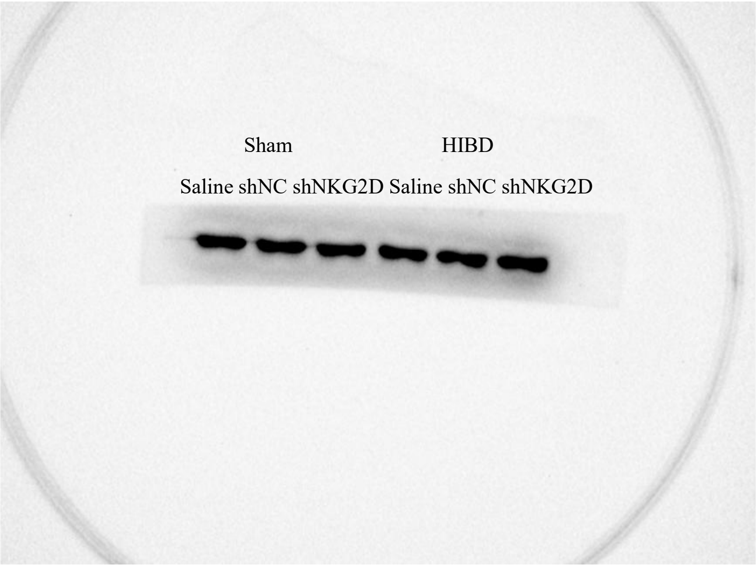
Sham HIBD
Saline shNC shNKG2D Saline shNC shNKG2D

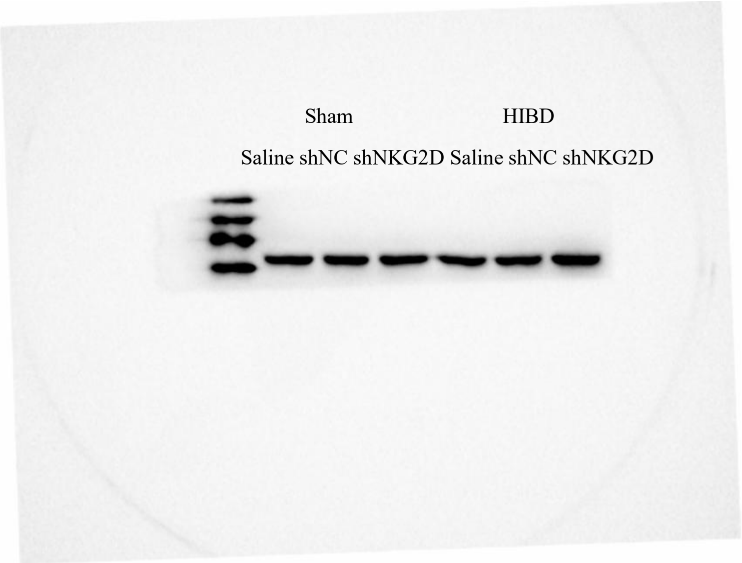
Sham
HIBD
Saline shNC shNKG2D Saline shNC shNKG2D

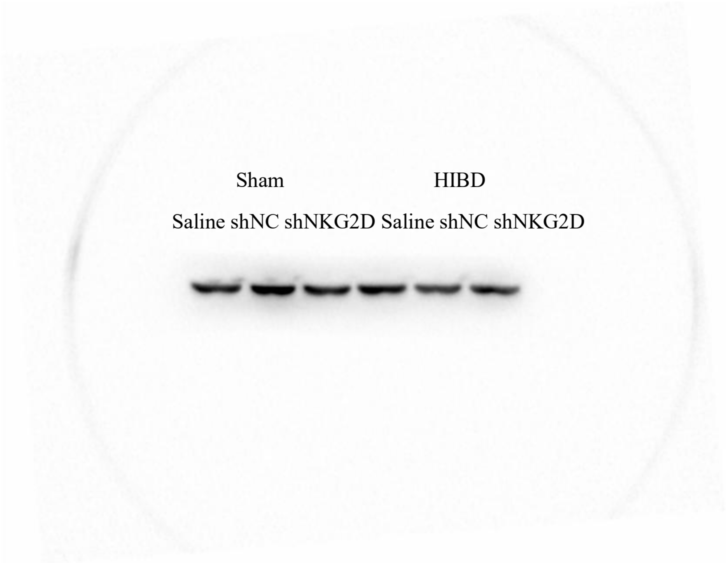
Sham
HIBD
Saline shNC shNKG2D Saline shNC shNKG2D

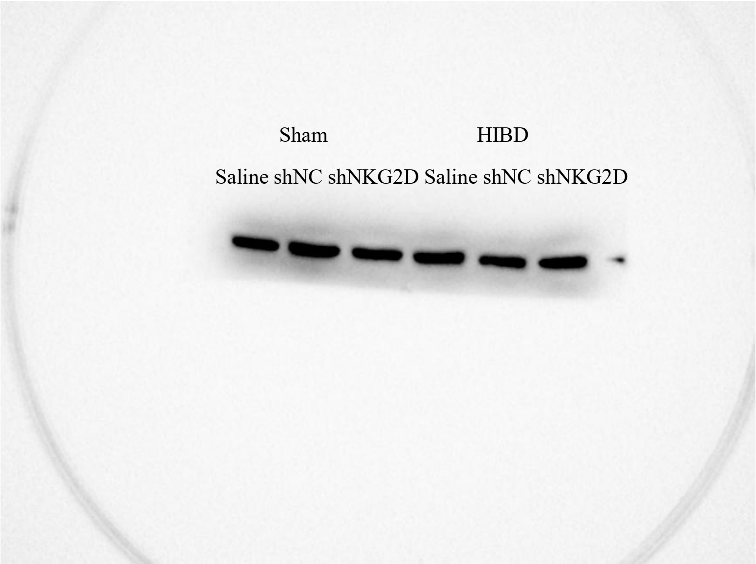
Sham
HIBD
Saline shNC shNKG2D Saline shNC shNKG2D

NF-κB p65

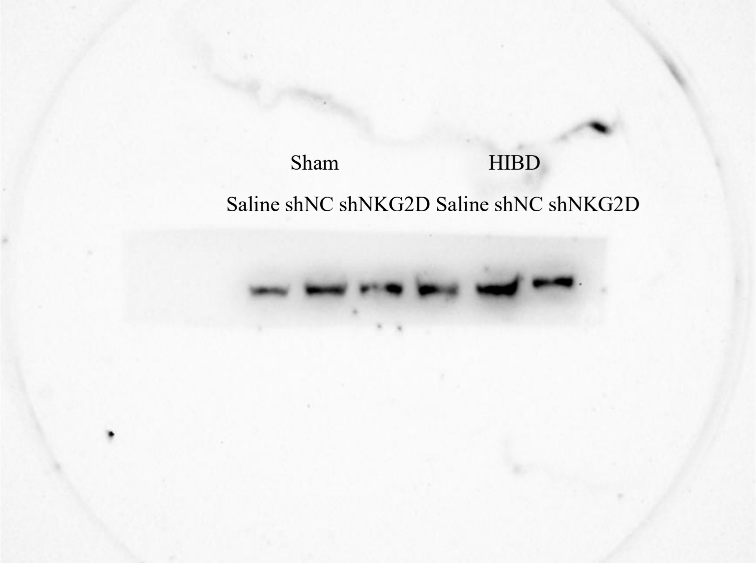
Sham
HIBD
Saline shNC shNKG2D Saline shNC shNKG2D

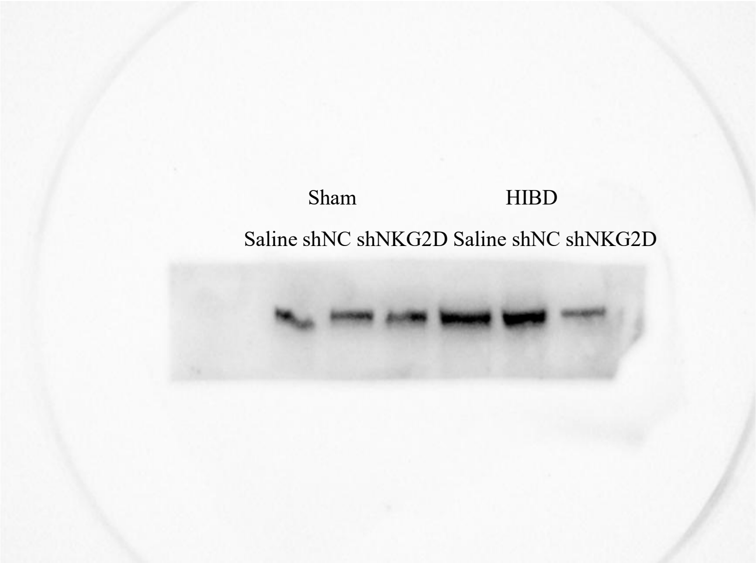
Sham
HIBD
Saline shNC shNKG2D Saline shNC shNKG2D

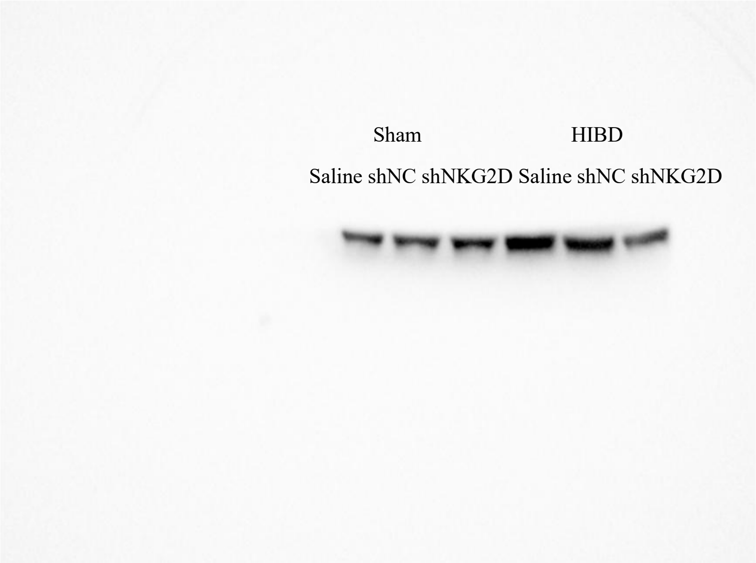
Sham
HIBD
Saline shNC shNKG2D Saline shNC shNKG2D

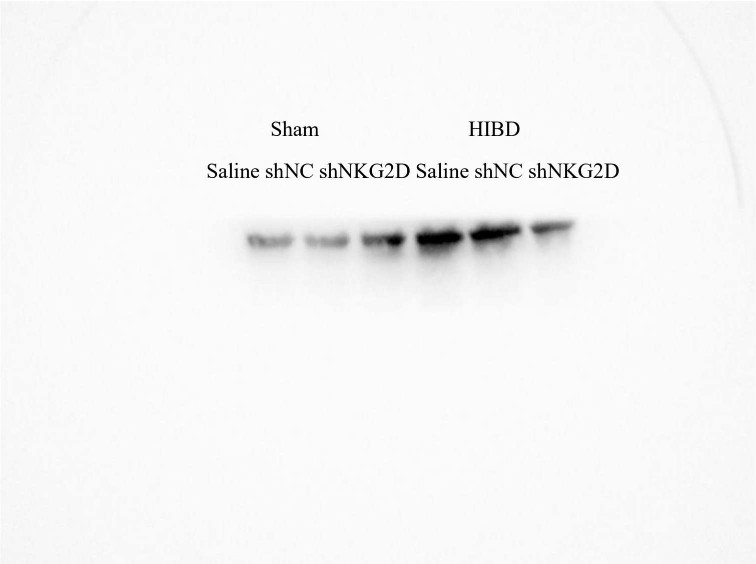
Sham
HIBD
Saline shNC shNKG2D Saline shNC shNKG2D

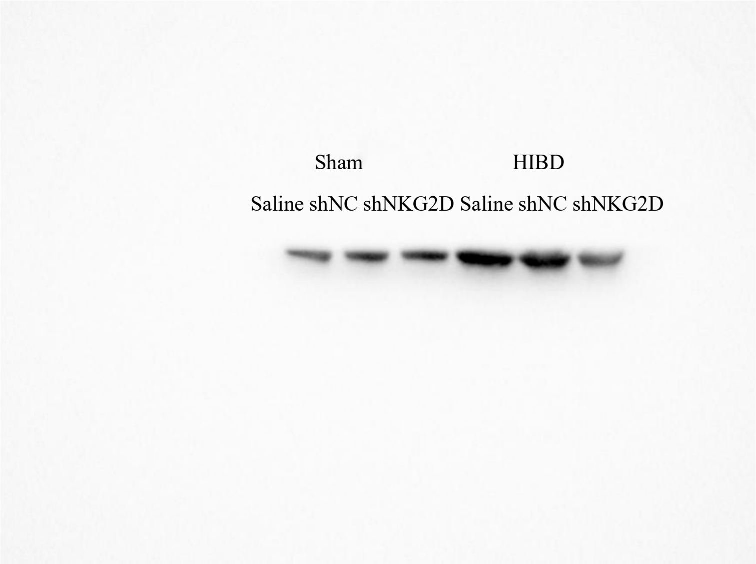
Sham
HIBD
Saline shNC shNKG2D Saline shNC shNKG2D

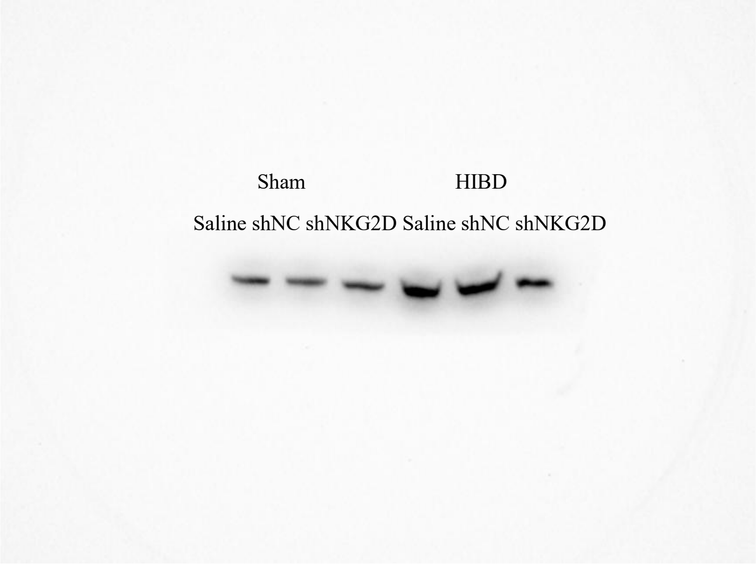
Sham
HIBD
Saline shNC shNKG2D Saline shNC shNKG2D

histone H3

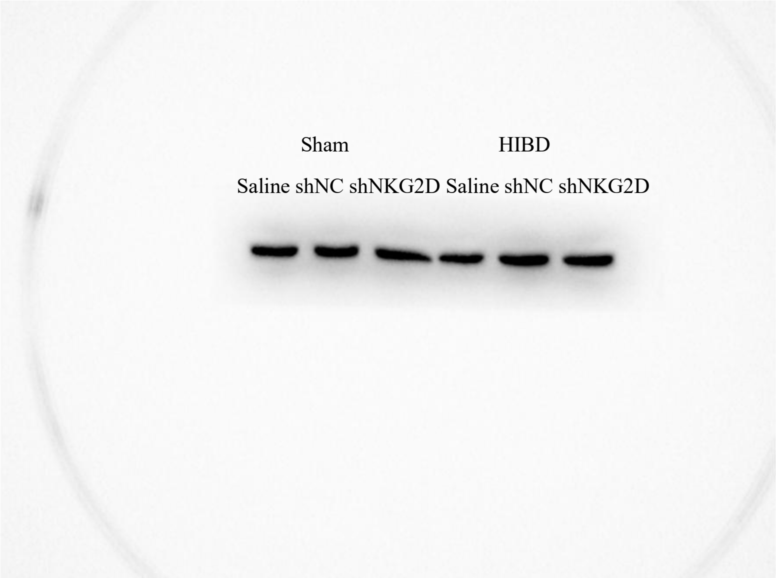
Sham
HIBD
Saline shNC shNKG2D Saline shNC shNKG2D

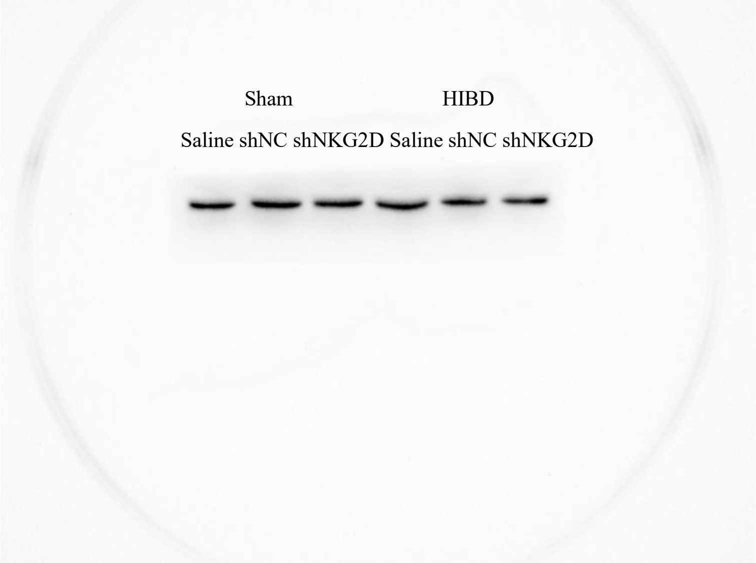
Sham
HIBD
Saline shNC shNKG2D Saline shNC shNKG2D

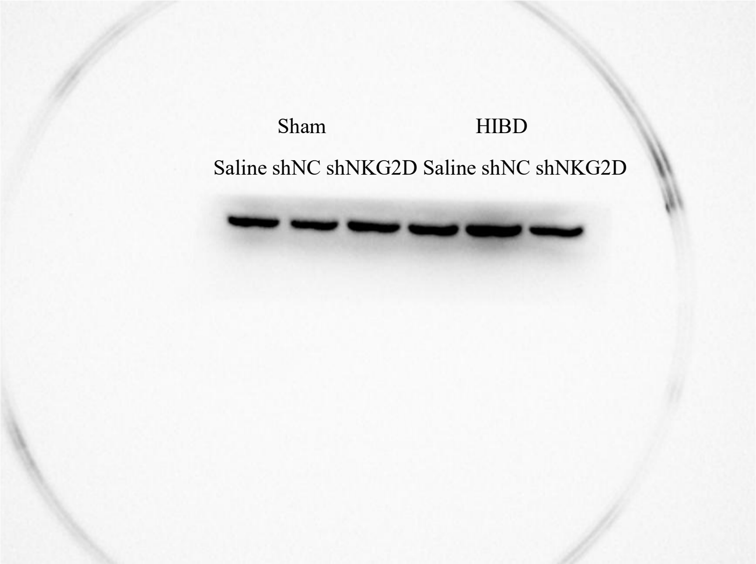
Sham
HIBD
Saline shNC shNKG2D Saline shNC shNKG2D

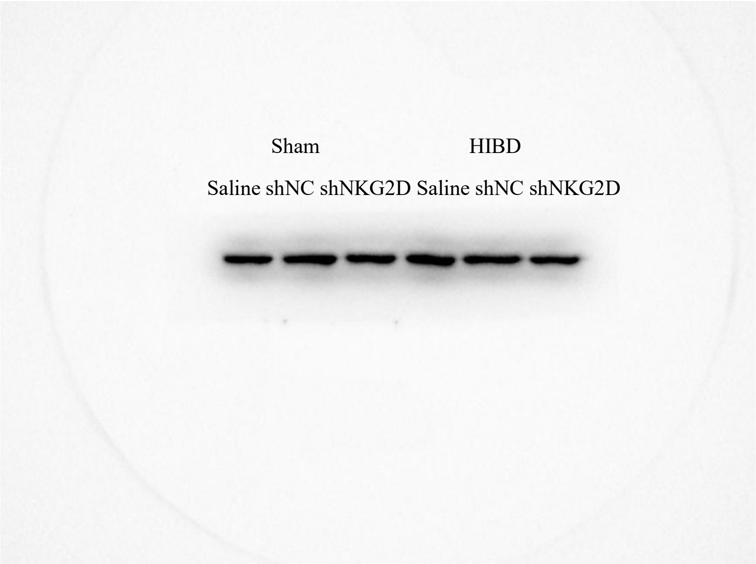
Sham
HIBD
Saline shNC shNKG2D Saline shNC shNKG2D

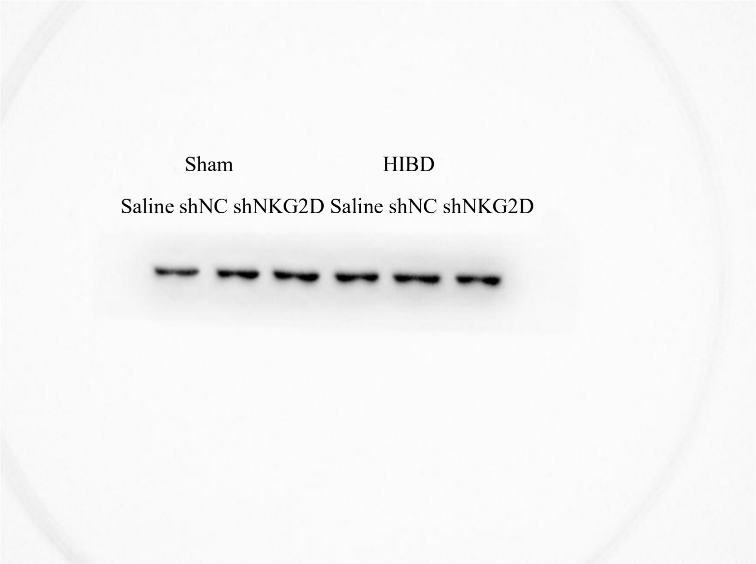
Sham
HIBD
Saline shNC shNKG2D Saline shNC shNKG2D

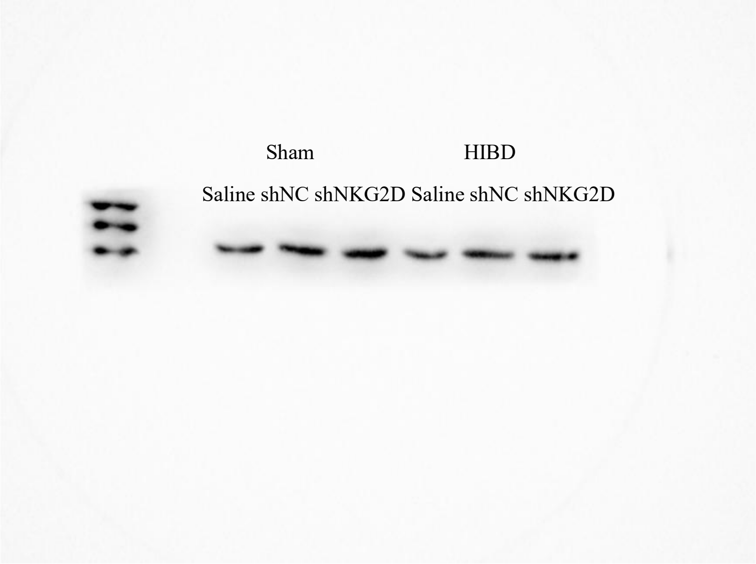
Sham
HIBD
Saline shNC shNKG2D Saline shNC shNKG2D
